# Sensing of Liver‐Derived Nicotinamide by Intestinal Group 2 Innate Lymphoid Cells Links Liver Cirrhosis and Ulcerative Colitis Susceptibility

**DOI:** 10.1002/advs.202404274

**Published:** 2024-08-09

**Authors:** Jing Shen, Zhen Li, Xiaoyu Liu, Mengqi Zheng, Peng Zhang, Yatai Chen, Qiuheng Tian, Wenyu Tian, Guanjun Kou, Yanyan Cui, Bowen Xu, Yunjiao Zhai, Weijia Li, Xiaohuan Guo, Ju Qiu, Chunyang Li, Ran He, Lixiang Li, Chunhong Ma, Yanqing Li, Xiuli Zuo, Detian Yuan, Shiyang Li

**Affiliations:** ^1^ Department of Gastroenterology Qilu Hospital of Shandong University Jinan 250012 China; ^2^ Advanced Medical Research Institute Shandong University Jinan 250012 China; ^3^ Shandong Provincial Clinical Research Center for Digestive diseases Jinan 250012 China; ^4^ Department of Biochemistry and Molecular Biology School of Basic Medical Sciences Shandong University Jinan 250012 China; ^5^ Institute for Immunology School of Medicine Tsinghua University Beijing 100084 China; ^6^ Beijing Key Lab for Immunological Research on Chronic Diseases Tsinghua University Beijing 100084 China; ^7^ CAS Key Laboratory of Tissue Microenvironment and Tumor Shanghai Institute of Nutrition and Health University of Chinese Academy of Sciences Chinese Academy of Sciences Shanghai 200031 China; ^8^ Key Laboratory for Experimental Teratology of Ministry of Education and Department of Histology and Embryology School of Basic Medical Sciences Cheeloo College of Medicine Shandong University Jinan 250012 China; ^9^ Department of Immunology School of Basic Medicine Tongji Medical College Huazhong University of Science and Technology Wuhan 43003 China; ^10^ Department of Immunology School of Basic Medical Sciences Cheeloo Medical College of Shandong University Jinan 250012 China

**Keywords:** group 2 innate lymphoid cells, liver injury, liver‐gut axis, NAD^+^ metabolism, nicotinamide phosphoribosyltransferase, ulcerative colitis

## Abstract

The correlation between liver disease and the progression of ulcerative colitis (UC) has remained elusive. In this study, it demonstrates that liver injury is intricately linked to the heightened severity of UC in patients, and causes more profound intestinal damage during DSS‐induced colitis in mice. Metabolomics analysis of plasma from liver cirrhosis patients shows liver injury compromising nicotinamide supply for NAD^+^ biosynthesis in the intestine. Subsequent investigation identifies intestinal group 2 innate lymphoid cells (ILC2s) are responsible for liver injury‐exacerbated colitis. Reconstitution of ILC2s or the restoration of NAD^+^ metabolism proves effective in relieving liver injury‐aggravated experimental colitis. Mechanistically, the NAD^+^ salvage pathway regulates gut ILC2s in a cell‐intrinsic manner by supporting the generation of succinate, which fuels the electron transport chain to sustaining ILC2s function. This research deepens the understanding of cellular and molecular mechanisms in liver disease‐UC interplay, identifying a metabolic target for innovative treatments in liver injury‐complicated colitis.

## Graphical abstract

Liver injury is intricately linked to the heightened severity of UC in patients, and causes more profound intestinal damage during DSS‐induced colitis in mice. Liver injury compromises nicotinamide supply for NAD^+^ biosynthesis in the intestinal ILC2s, contributing to liver injury‐exacerbated colitis. Mechanistically, the NAD^+^ salvage pathway sustains gut ILC2s by supporting succinate generation, which fuels the electron transport chain.

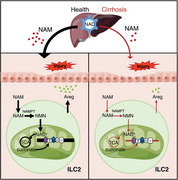

## 1 Introduction

Inflammatory bowel disease (IBD), including ulcerative colitis (UC) and Crohn's disease, constitutes multifaceted disorders with chronic and relapsing intestinal inflammation. Although the cause of IBD remains unknown, disturbed intestinal immune system and microbiota on the basis of genetic susceptibility are considered to be involved in the pathophysiology of IBD.^[^ ^1^ ^]^ Additionally, the increased IBD severity is noticed in IBD patients with abnormality in other organs, such as liver.^[^ ^2^, ^3^ ^]^ It is widely acknowledged that the primary sclerosing cholangitis heightens incidence of IBD, and increases the risk of IBD‐associated colorectal cancers.^[^ ^3^ ^]^ However, the mechanism underlying the exacerbation of IBD by liver disease is not completely understood.

In mammals, liver makes nicotinamide adenine dinucleotide (NAD^+^) *de novo* from tryptophan, releasing nicotinamide (NAM) into the blood for NAD^+^ salvage pathway by peripheral tissues to meet NAD^+^ demand.^[^ ^4^ ^]^ Fundamentally, NAD^+^ receives high‐energy electrons from glycolytic and tricarboxylic acid (TCA) intermediates and eventually feeds electrons into complex I of the electron transport chain (ETC) to drive oxidative phosphorylation, and adenosine triphosphate (ATP) production.^[^ ^5^ ^]^ Arisen evidences highlight NAD^+^ metabolism in programming immune responses in the gut during colitis, e.g., NAD^+^ depletion alters macrophage polarization to ameliorate DSS‐induced colitis.^[^ ^6^ ^]^ However, whether NAD^+^, mainly circulated from liver, has effect on tissue repair by regulating gut immune cells in colitis remains unclear.

Group 2 innate lymphoid cells (ILC2s) expressing the transcription factor GATA3 take part in the maintenance of intestinal homeostasis by promoting epithelial repair and barrier integrity. ILC2s are regulated by a large range of tissue signals, including prostaglandins, neuropeptides, metabolic cues, and alarmins such as IL‐25, IL‐33, and IL‐18 released by damaged epithelial cells.^[^ ^7^ ^]^ Upon intestine injury, IL‐33 released by necrotic cells promotes ILC2s accumulation and type 2 cytokines secretion, concomitant with amphiregulin (Areg) production, to elevate tissue repair by enhancing epithelial cell proliferation, stem cell renewal and mucin production by goblet cells in DSS colitis.^[^ ^8^, ^9^, ^10^, ^11^ ^]^

Immunometabolism has shown an emerging role in modulating the function of ILC2s. Arginase1, a key enzyme in arginine metabolism, serves as a metabolic checkpoint controlling ILC2s function.^[^ ^12^ ^]^ Upon exposure to IL‐33, external lipids of ILC2s are converted into phospholipids to promote ILC2s proliferation.^[^ ^13^, ^14^ ^]^ S‐adenosylmethionine links environmental allergic signals and ILC2s response.^[^ ^15^ ^]^ Circulating naive human ILC2s reside in an active metabolic state dependent on mitochondrial oxidative phosphorylation (OXPHOS).^[^ ^16^ ^]^

In this study, we demonstrated that liver injury was associated with the aggravated symptoms in UC patients, and liver injury led to severe intestinal damage during DSS‐induced colitis in mice. Mechanistically, diminished ILC2s function, caused by decreased NAD^+^ in the gut as a consequence of disordered liver NAD^+^ biogenesis, was responsible for the exacerbation of experimental colitis in mice. Our findings link the liver fibrosis and worsened colitis in mouse model, in accordance with the concurrency of certain liver disease and UC, providing new insights into the metabolic control of gut ILC2s by NAD^+^ metabolism and a potential therapeutic target of liver disease‐aggravated colitis.

## 2 Results

### 2.1 Liver Injury is Associated with Severe Symptoms in UC and Experimental Colitis

**Figure 1:**
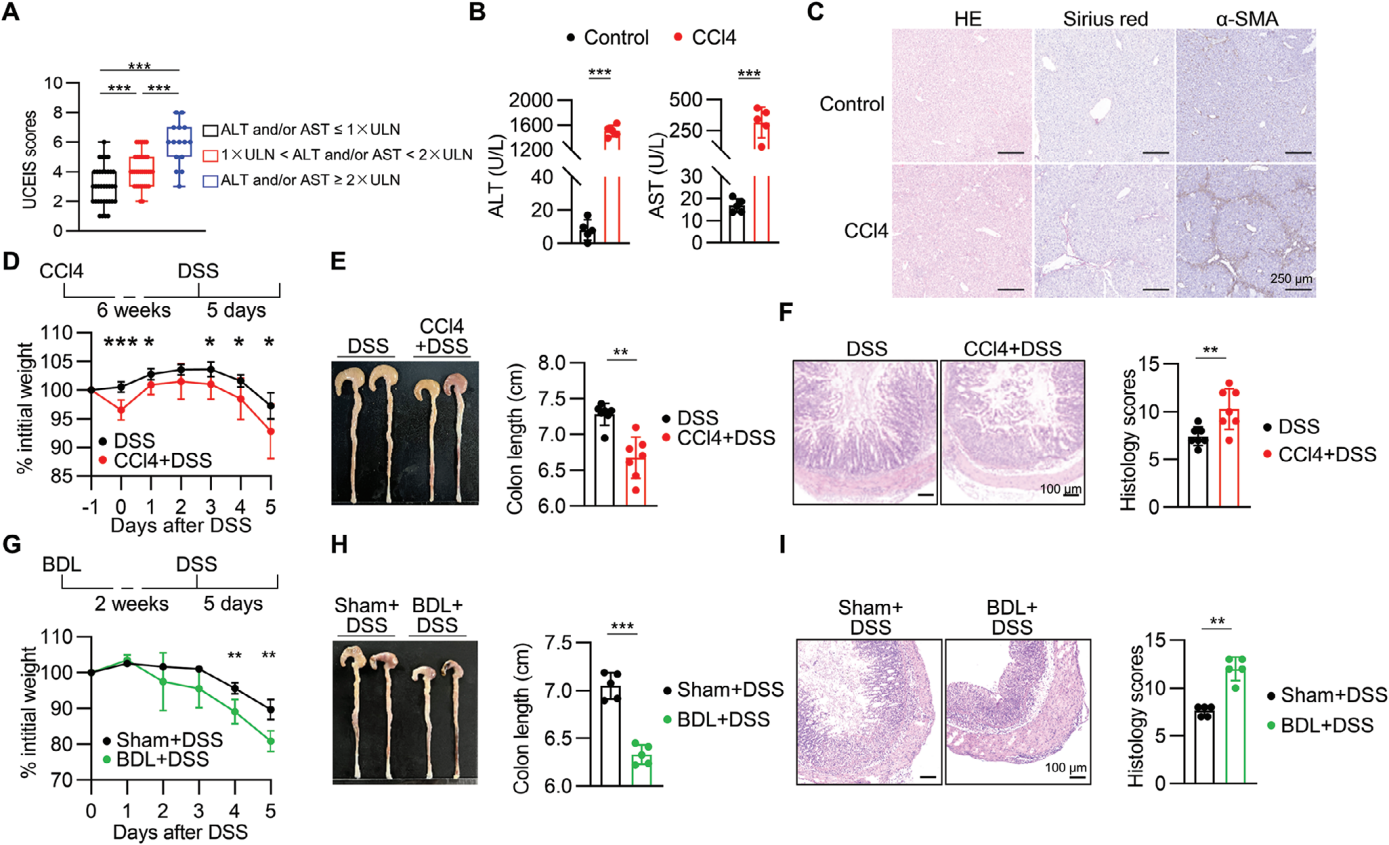
Liver injury is associated with severe symptoms in UC and experimental colitis. A) Ulcerative colitis endoscopic index of severity (UCEIS) scores in UC patients with or without liver injury. Liver injury was defined as the levels of alanine aminotransferase (ALT) and/or aspartate aminotransferase (AST) exceeding the upper limit of normal (ULN). n = 35 for ALT and/or AST ≤ 1 × ULN group, n = 35 for 1 × ULN < ALT and/or AST < 2 × ULN group, and n = 15 for ALT and/or AST ≥ 2 × ULN group. Box‐and‐whisker plot representing the median (line within the box), the interquartile range (length of the box), the min and the max (whiskers above and below the box) of UCEIS. B,C) Mice were intraperitoneally injected with carbon tetrachloride (CCl4, 2 mL kg^−1^, twice a week) for 6 weeks. Serum ALT and AST levels (B), hematoxylin‐eosin (H&E) and Sirius Red staining, and alfa‐smooth muscle actin (*a*‐SMA) immunohistochemistry staining of liver (C). Data were presented as mean ± SD; n = 5 per group, from two independent experiments. D–F) Mice were intraperitoneally injected with CCl4 (2 mL kg^−1^, twice a week) for 6 weeks before DSS administration. The experimental outline is shown (D, top). Weight course (D, bottom). Day 0 represents the starting day for DSS treatment. The body weight was calculated as percent of the original body weight (day −1). Representative colons and colon lengths (E), representative H&E staining and histological severity scores of colons (F), mean ± SD of 7 biological replicates, from two independent experiments. G–I) Mice were subjected to bile duct ligation (BDL) or sham operation. After a period of 2 weeks, mice were fed with DSS in drinking water. The experimental outline is shown (G, top). Weight course (G, bottom). Day 0 represents the starting day for DSS treatment. The body weight was calculated as percent of the original body weight (day 0). Representative colons and colon lengths (H), representative H&E staining of colons and histological severity scores (I); mean ± SD of 5 biological replicates, from two independent experiments. The two‐sided unpaired t‐test or two‐sided Mann–Whitney U‐test according to normal distribution in A, B, D‐I. The statistical test method and exact *P* value are detailed in the Table S4 (Supporting Information). ^*^ *p* < 0.05, ^**^ *p* < 0.01, ^***^ *p* < 0.001.

To investigate the effect of liver injury on colitis, the ulcerative colitis endoscopic index of severity (UCEIS) was analyzed in UC patients classified by the levels of alanine aminotransferase (ALT) and aspartate aminotransferase (AST) in the serum using retrospective chart review, and higher UCEIS scores were observed in liver injury‐suffering UC patients whose ALT or AST is greater than upper limit of normal (ULN) (**Figure** **1**A; Figure S1A,B and Table S1, Supporting Information). Additionally, the Pearson's analysis unveiled a positive correlation between UCEIS scores and ALT or AST (Figure S1C,D, Supporting Information). To test this finding in mice, we modeled liver injury using carbon tetrachloride (CCl4)‐induced liver cirrhosis followed by DSS‐induced colitis (Figure 1B–F). Mice were injected with CCl4 for 6 weeks, and the appearance of liver cirrhosis was confirmed by elevated serum ALT and AST levels, and severe liver fibrosis was assessed by histopathological analysis with staining of hematoxylin‐eosin (H&E), Sirius Red and *α*‐smooth muscle actin (*α*‐SMA) (Figure 1B,C). No significant inflammation and epithelial barrier alteration in the large intestine (LI) of mice with CCl4‐induced liver injury, revealed by colon length, H&E and immunohistochemistry (Figure S1E‐G, Supporting Information). However, mice subjected to DSS‐induced colitis model after CCl4 treatment showed exacerbated disease, in terms of accelerated weight loss, shortened colon, and increased histopathological severity compared to DSS‐feeding mice without CCl4 (Figure 1D–F). To rule out the possibility that CCl4, as a chemical, instead of its induction of liver injury contributed to the phenomenon, bile duct ligation (BDL)‐induced liver fibrosis model was conducted (Figure S1H, Supporting Information). In line with CCl4 model, mice receiving BDL surgery were more susceptible to DSS‐induced colitis (Figure 1G–I), while no tissue damage was noticed in the colon of BDL‐bearing mice without induction of colitis (Figure S1I,J, Supporting Information). Taken together, these findings demonstrate that liver injury correlates with severe UC in human, and exacerbates DSS‐induced colitis in mice.

### 2.2 Liver Injury Impairs NAD^+^ Metabolism in Human and Mice

**Figure 2:**
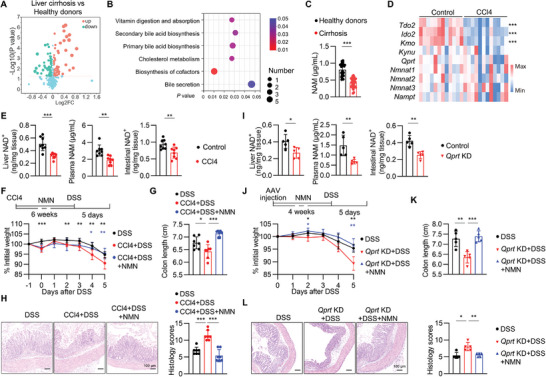
Liver injury impairs NAD^+^ metabolism in human and mice. A,B) Metabolomics analysis of healthy donors and liver cirrhosis patients’ plasma. (A) Volcano chart of differential metabolites in healthy donors and liver cirrhosis patients. (B) KEGG pathway analysis for differential metabolites between healthy donors and liver cirrhosis patients’ plasma. n = 9 per group. C) NAM concentration in plasma from healthy donors and liver cirrhosis patients were measured by HPLC‐MSMS. Data were presented as mean ± SD; n = 30 per group. D) Mice were intraperitoneally injected with CCl4 (2 mL kg^−1^, twice a week) for 6 weeks. Quantitative reverse transcription polymerase chain reaction (qRT‐PCR) analysis of the mRNA levels of indicated genes in liver, n = 12 per group, from four independent experiments. E) Mice were intraperitoneally injected with CCl4 (2 mL kg^−1^, twice a week) for 6 weeks. Liver NAD^+^, plasma NAM and intestinal NAD^+^ contents were measured by HPLC‐MSMS; mean ± SD of 8 biological replicates, from three independent experiments. F–H) Mice were intraperitoneally injected with CCl4 (2 mL kg^−1^, twice a week) and orally administrated nicotinamide mononucleotide (NMN) for 6 weeks before DSS administration. The experimental outline is shown (F, top). Weight course (F, bottom). Day 0 represents the starting day for DSS treatment. The body weight was calculated as percent of the original body weight (Day −1). Colon length (G), representative hematoxylin‐eosin (H&E) staining and histological severity scores of colons (H); mean ± SD of 7 biological replicates, from two independent experiments. I) Mice were injected with liver‐specific AAV carrying siRNA targeting *Qprt* or control siRNA. After a period of 4 weeks, liver NAD^+^, plasma NAM and intestinal NAD^+^ contents were measured by HPLC‐MSMS, mean ± SD of 5 biological replicates, from two independent experiments. J–L) Mice were injected with liver‐specific AAV carrying siRNA targeting *Qprt* or control siRNA, and orally administrated NMN. After a period of 4 weeks, they were subjected to DSS feeding. The experimental outline is shown above (J, top). Weight course (J, bottom). Day 0 represents the starting day for DSS treatment. The body weight was calculated as percent of the original body weight (Day 0). Colon length (K), representative H&E staining and histological severity scores of colons (L); mean ± SD of 5 biological replicates, from two independent experiments. The two‐sided unpaired t‐test or two‐sided Mann–Whitney U‐test according to normal distribution in C‐L. The statistical test method and exact *P* value are detailed in the Table S4 (Supporting Information). ^*^ *p* < 0.05, ^**^ *p* < 0.01, ^***^ *p* < 0.001.

The liver is a source of circulating factors that promote metabolic homeostasis of various organs, including the gut. To explore the mechanism underlying the association of liver injury and exacerbated colitis, we performed non‐targeted metabolomics on plasma from healthy donors and patients with cirrhosis, a consequence of long‐term liver injury. Results showed that the metabolomes of the plasma from liver cirrhosis patients and healthy donors clustered separately (Figure S2A, Supporting Information). There were 126 differentially regulated metabolites, of which 67 decreased in liver cirrhosis patients (**Figure** **2**A). KEGG pathway analysis showed that six metabolic pathways were significantly different between liver cirrhosis patients and healthy donors (Figure 2B), while biosynthesis of cofactors was the most different pathway, which was determined by decreased levels of L‐Serine, NAM, pantothenic acid, and retinol, and increased levels of deoxycholic acid 3‐glucuronide and L‐tyrosine (Figure S2B, Supporting Information).

NAM, an important intermediate metabolite of NAD^+^ metabolism in the liver, is critical for the NAD^+^ biosynthesis in the peripheral organs that contributes to the maintenance of tissue and metabolic homeostasis.^[^ ^5^ ^]^ The reduction of plasma NAM in the patients with cirrhosis was confirmed using high‐performance lipid chromatography‐mass spectrometry (Figure 2C). Additionally, publicly available RNA‐seq data revealed that the mRNA levels of tryptophan 2,3‐dioxygenase (*TDO2*), 3‐hydroxyanthranilic acid dioxygenase (*HAAO*), and quinolinate phosphoribosyl transferase (*QPRT*), encoding key enzymes in the *de novo* and salvage biosynthesis pathway for NAD^+^ synthesis, were decreased in the liver samples from patients with non‐alcoholic steatohepatitis (GSE49541)^[^ ^17^ ^]^ or alcoholic hepatitis (GSE28619)^[^ ^18^ ^]^ (Figure S2C–E, Supporting Information). The liver cirrhosis reduced *Tdo2*, indoleamine‐2,3‐dioxygenase 1 (*Ido2*) and kynurenine 3‐monooxygenase (*Kmo*) mRNA levels in the liver (Figure 2D), as well as liver NAD^+^, plasma NAM and intestinal NAD^+^ levels in mice (Figure 2E; Figure S2F, Supporting Information). Consistently, metabolic associated fatty liver disease (MAFLD, GSE200482)‐ or BDL (GSE40041)‐induced liver fibrosis in mice reduced NAD^+^ levels in liver^[^ ^19^ ^]^ (Figure S2G–I, Supporting Information), demonstrating that liver injury impaired NAD^+^ metabolism in mice.

We sought to test whether liver injury aggravates DSS colitis owing to impaired NAD^+^ metabolism. NMN was supplemented to restore NAD^+^ biosynthesis during CCl4 treatment. Liver damage, assessed by histopathological analysis, as well as plasma NAM and intestinal NAD^+^ levels were rescued in CCl4‐treated mice receiving NMN (Figure S3A,B, Supporting Information). Accordingly, the productions of Areg, IL‐5, and IL‐13 in the gut ILC2s were recovered by NMN in CCl4‐treated mice (Figure S3C,D, Supporting Information). Of note, NMN supplementation protected mice from CCl4‐ or BDL‐aggravated DSS colitis based on ameliorated weight loss, increased colon length, reduced histological severity of colitis, and decreased expression of inflammatory cytokines, such as *Il1β* and *Il6* (Figure 2F‐H; Figure S4E‐H, Supporting Information).

To verify the impact of hepatic NAD^+^ metabolism on the progression of colitis, we disrupted NAD^+^ metabolism specifically in the liver by knockdown of QPRT using AAV2/8‐TBG‐mir30‐*m*‐*Qprt*‐ZsGreen (*Qprt‐*KD), which successfully decrease the liver NAD^+^, plasma NAM and intestinal NAD^+^ levels, which was rescued by NMN (Figure 2I; Figure S3I,J, Supporting Information). Accordingly, *Qprt*‐KD downregulated the production of effector molecules by gut ILC2s (Figure S3K, Supporting Information), and showed aggravated disease symptoms in DSS‐induced colitis, which was rescued by NMN (Figure 2J‐L). It is worth noting that AAV vectors are known to activate both innate and adaptive responses, potentially inducing hepatotoxicity.^[^ ^20^, ^21^, ^22^ ^]^ Our findings indicate that AAV vector infection led to reduced liver NAD^+^, plasma NAM, and intestinal NAD^+^ levels (Figure 2I), compared to the control mice without AAV administration (Figure 2E). This suggests a potential impact of AAV vectors on NAD^+^ metabolism due to hepatotoxicity, necessitating further research for validation.

### 2.3 Liver Injury Undermines Intestinal ILC2s

**Figure 3:**
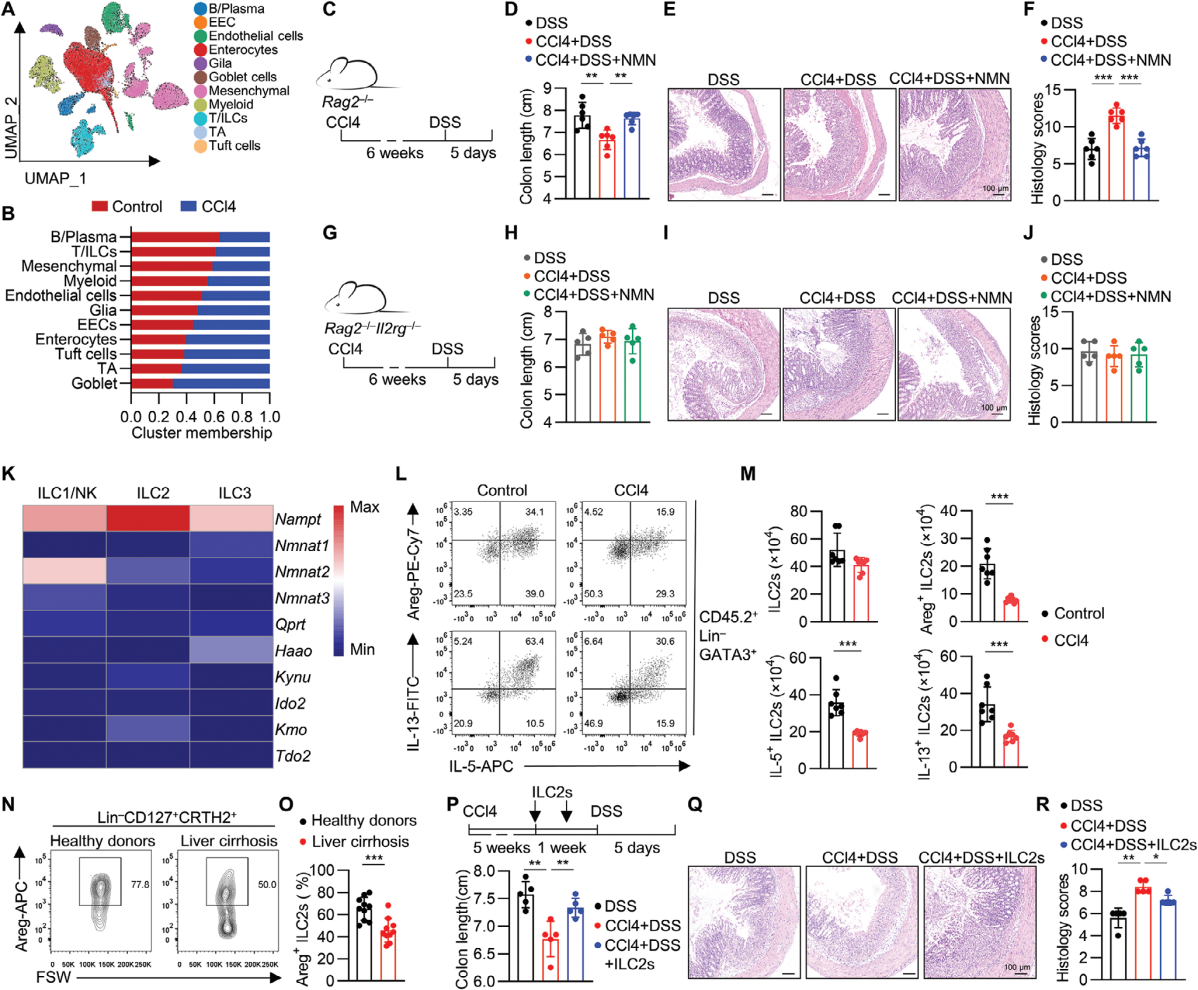
Liver injury undermines intestinal ILC2s. A) scRNA‐seq was performed with colonic tissue from the mice treated with carbon tetrachloride (CCl4, 2 mL kg^−1^, twice a week) for 6 weeks. Uniform manifold approximation and projection (UMAP) plots showing the cell clusters. EEC, enteroendocrine cells; TA, Transit amplifying cell. B) Cell percentage of the clusters within CCl4 and control groups. C–F) *Rag2* ^–/–^ mice were intraperitoneally injected with CCl4 (2 mL kg^−1^, twice a week) and orally administrated NMN for 6 weeks before DSS administration. Experimental strategy (C). Colon lengths (D), representative hematoxylin‐eosin (H&E) staining (E) and histological severity scores of colons (F), mean ± SD of 6 biological replicates, from two independent experiments. G–J) *Rag2* ^–/–^ *Il2rg* ^–/–^ mice were intraperitoneally injected with CCl4 (2 mL kg^−1^, twice a week) and orally administrated NMN for 6 weeks before DSS administration. Experimental strategy (G). Colon lengths (H), representative H&E staining (I) and histological severity scores of colons (J); mean ± SD of 5 biological replicates, from two independent experiments. K) Heatmap showing the mRNA expression of NAD^+^ synthesis pathway genes in ILCs. L,M) Mice were intraperitoneally injected with CCl4 (2 mL kg^−1^, twice a week) for 6 weeks. Representative FACS analyses (L) and absolute numbers of ILC2s, Areg^+^ ILC2s, IL‐5^+^ ILC2s, and IL‐13^+^ ILC2s (M) in large intestine; mean ± SD of 7 biological replicates, from 3 independent experiments. N,O) Representative FACS analyses (N) and frequency of Areg^+^ ILC2s (O) in PBMCs of healthy donors and liver cirrhosis patients; mean ± SD of 10 biological replicates. P–R) Mice were intraperitoneally injected with CCl4 (2 mL kg^−1^, twice a week) for 6 weeks and ILC2s were transferred to mice via tail vein injection two times before DSS treatment. Experimental strategy (P, top). Colon lengths (P, bottom), representative H&E staining (Q) and histological severity scores of colons (R); mean ± SD of 5 biological replicates, from two independent experiments. The two‐sided unpaired t‐test or two‐sided Mann–Whitney U‐test according to normal distribution in D, F, H, J, M, O, P, R. The statistical test method and exact *P* value are detailed in the Table S4 (Supporting Information). ^*^ *p* < 0.05, ^**^ *p* < 0.01, ^***^ *p* < 0.001.

To investigate the mechanism underlying the exacerbation of colitis by liver injury, we carried out single‐cell RNA sequencing (scRNA‐seq) on colonic tissues from CCl4‐treated or control mice (**Figure** **3**A,B). After data preprocessing and quality control, we obtained single‐cell transcriptomes of 43249 cells. The cells were classified into 11 clusters based on differentially expressed and characteristic genes (Figure 3A; Figure S4A, Supporting Information). CCl4 treatment led to increase of intestinal enterocytes and secretory epithelial cells, suggesting that CCl4‐induced liver injury may not disrupt gut epithelium to aggravate DSS‐induced colitis (Figure 3B). Notably, decreased cell populations caused by CCl4 administration include B/plasma cell, T/ILCs, and mesenchymal cells (Figure 3B), among which T/ILCs compartment is intensively involved in the progression of colitis.^[^ ^23^, ^24^ ^]^

To investigate the effects of B/T/ILCs in liver injury‐aggravated colitis, we administrated CCl4, along with or without NMN supplementation, to mice lacking T and B cells (*Rag2* ^–/–^) or those deficient in T, B cells, and ILCs (*Rag2* ^–/–^ *Il2rg* ^–/–^),^[^ ^25^, ^26^ ^]^ followed by DSS feeding. NMN administration protected *Rag2* ^–/–^ mice from CCl4‐aggravated DSS colitis (Figure 3C‐F), but no discernible impact was observed in CCl4‐combined DSS colitis in *Rag2* ^–/–^ *Il2rg* ^–/–^ mice (Figure 3G‐J). Together, these results suggest that liver injury‐impaired NAD^+^ metabolism and consequent compromised gut ILCs contributes to the liver injury‐exacerbated colitis.

Further, our scRNA‐seq analysis showed that the mRNA level of nicotinamide phosphoribosyltransferase (*Nampt*), encoding rate‐limiting enzyme of NAD^+^ salvage pathway, is expressed at the highest level in ILC2s compared to other ILC subsets in the gut (Figure 3K), suggesting that ILC2s might be the corresponding ILC subset in liver injury‐exacerbated colitis. Indeed, decreased expression of ILC2 characteristic genes was observed in CCl4‐treated mice (Figure S4B, Supporting Information). Consistently, flow cytometry analysis revealed that CCl4 treatment compromised the production of effector molecules by gut ILC2s, such as Areg, IL‐5, and IL‐13 (Figure 3L,M; Figure S4C,D, Supporting Information), which participate in the process of tissue repair in colitis.^[^ ^8^ ^]^ Similarly, BDL surgery also disturbed the production of Areg, IL‐5, and IL‐13 by gut ILC2s (Figure S4E‐H, Supporting Information). Clinically, human ILC2s were suppressed in liver cirrhosis patients, as evidenced by decreased Areg expression by the ILC2s in human peripheral blood mononuclear cells from liver cirrhosis patients, compared to that of healthy donors (Figure 3N,O). To test whether compromised ILC2 function accounted for the exacerbation of DSS‐induced colitis in mice with CCl4‐induced liver cirrhosis, purified gut ILC2s were adoptively transferred into CCl4‐treated mice, followed by DSS treatment (Figure 3P‐R). Restoring ILC2s ameliorated CCl4‐aggravated DSS colitis, revealed by increased colon length and reduced histopathological scores (Figure 3P‐R). Together, these results validate that liver injury undermines intestinal ILC2s, leading to exacerbation of colitis.

### 2.4 Intestinal ILC2s Maintenance Depends on NAD^+^ Salvage Pathway

**Figure 4:**
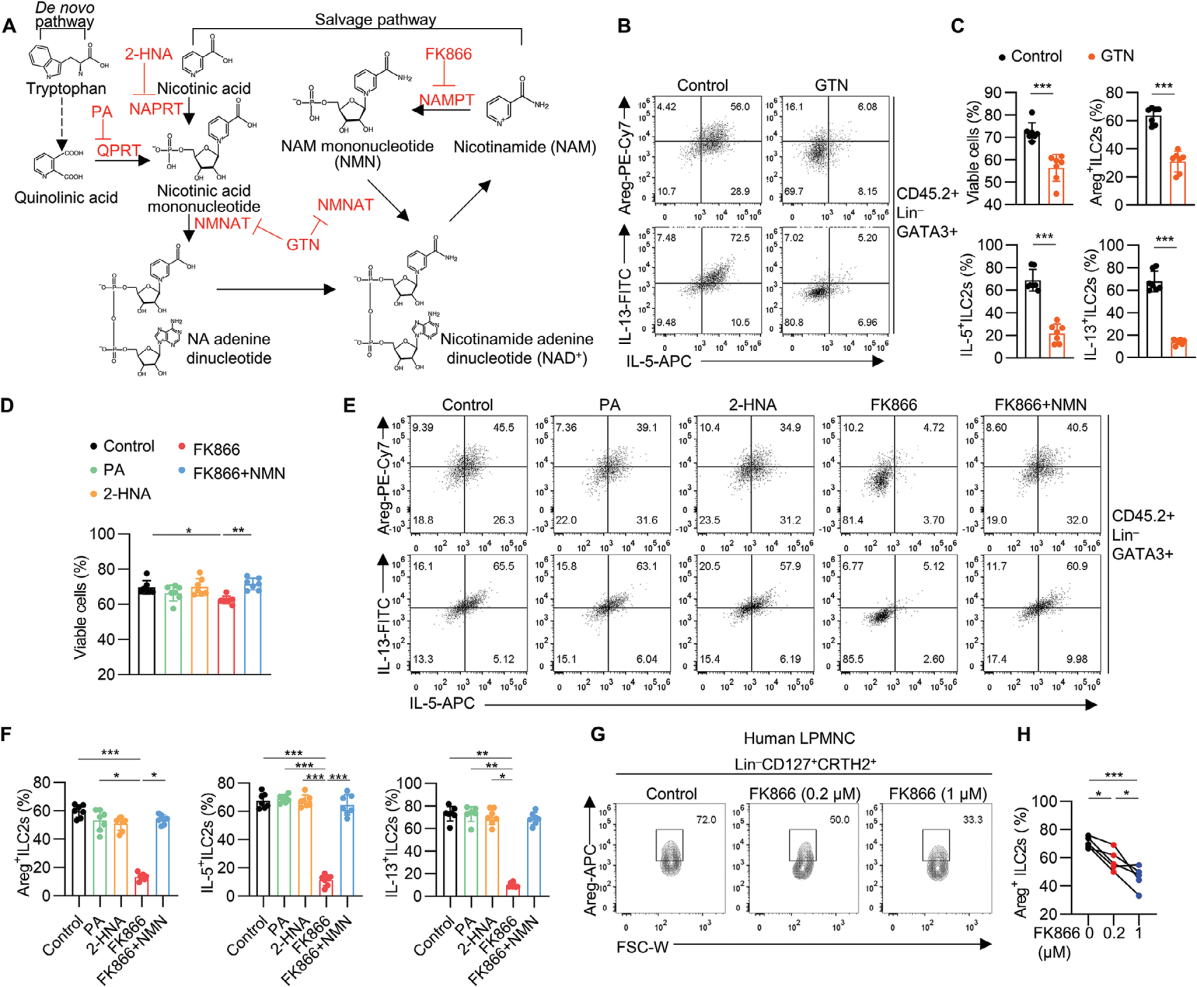
Intestinal ILC2s maintenance depends on NAD^+^ salvage pathway. A) Schematic representation of the NAD^+^ synthesis pathway and key enzymes. B,C) Sorted large intestinal ILC2s were treated with nicotinamide mononucleotide adenylyltransferease (NMNAT) inhibitor gallotannin (GTN, 50 µm) for 16 h. Flow cytometry analysis of viability and cytokines expression in ILC2s (CD45.2^+^Lin^–^GATA3^+^); mean ± SD of 7 biological replicates, from three independent experiments. D–F) Sorted large intestinal ILC2s were treated with or without quinolinate phosphoribosyl transferase (QPRT) inhibitor phthalic acid (PA, 500 µm), nicotinate phosphoribosyltransferase (NAPRT) inhibitor 2‐hydroxynicotinic acid (2‐HNA, 1 mm), nicotinamide phosphoribosyltransferase (NAMPT) inhibitor FK866 (20 nM), or NMN (1 mm) for 16 h. Flow cytometry analysis of viability (D), representative FACS analyses (E) and cytokines expression (F) in ILC2s (CD45.2^+^Lin^–^GATA3^+^); mean ± SD of 7 biological replicates, from three independent experiments. G,H) Representative FACS analyses (G) and frequency of Areg^+^ ILC2s (Lin^–^CD127^+^CRTH2^+^) (H) in healthy donors lamina propria mononuclear cells (LPMNCs) in the presence or absence of FK866. Data were presented as mean ± SD; n = 5 per group. The two‐sided unpaired t‐test or two‐sided Mann–Whitney U‐test according to normal distribution in C. The One‐way ANOVA with Tukey or Kruskal Wallis with Dunns according to normal distribution in D, F, H. The statistical test method and exact *P* value are detailed in the Table S4 (Supporting Information). ^*^ *p* < 0.05, ^**^ *p* < 0.01, ^***^ *p* < 0.001.

The NAD^+^ is synthesized *de novo* from tryptophan or from vitamin precursors such as nicotinic acid, or regenerated via salvage pathway by recycling degraded NAD^+^ metabolites such as NAM (**Figure** **4**A). To assess whether NAD^+^ metabolism directly regulates ILC2s, sorted ILC2s from the gut were treated with gallotannin (GTN), a known inhibitor of NMN adenylyl transferases (NMNATs) that generate NAD^+^ downstream of *de novo* and salvage pathways (Figure 4A). The results showed that GTN significantly inhibited the cell viability and the production of effector molecules of ILC2s (Figure 4B,C). FK866, the inhibitor of NAMPT, markedly reduced the viability and cytokines production of sorted ILC2s, which was reversed by NMN replenishment (Figure 4D‐F). Inhibition of QPRT by phthalic acid (PA), or nicotinate phosphoribosyltransferase (NAPRT) by 2‐hydroxynicotinic acid (2‐HNA), had no impact on ILC2s, in line with the low expression of these two genes in ILC2s (Figures 3K and 4D–F). To test whether the requirement of NAMPT in mouse ILC2s is also valid in human ILC2s, human lamina propria mononuclear cells (LPMNC) were isolated from healthy donors, and cultured in presence of FK866. Dose‐dependent reduction of Areg was noticed in Lin^–^CD127^+^CRTH2^+^ ILC2s (Figure 4G,H). Collectively, these results suggest that NAD^+^ salvages synthesis is indispensable for the maintenance and function of intestinal ILC2s.

### 2.5 NAD^+^ Supports Intestinal ILC2s via Feeding ETC

**Figure 5:**
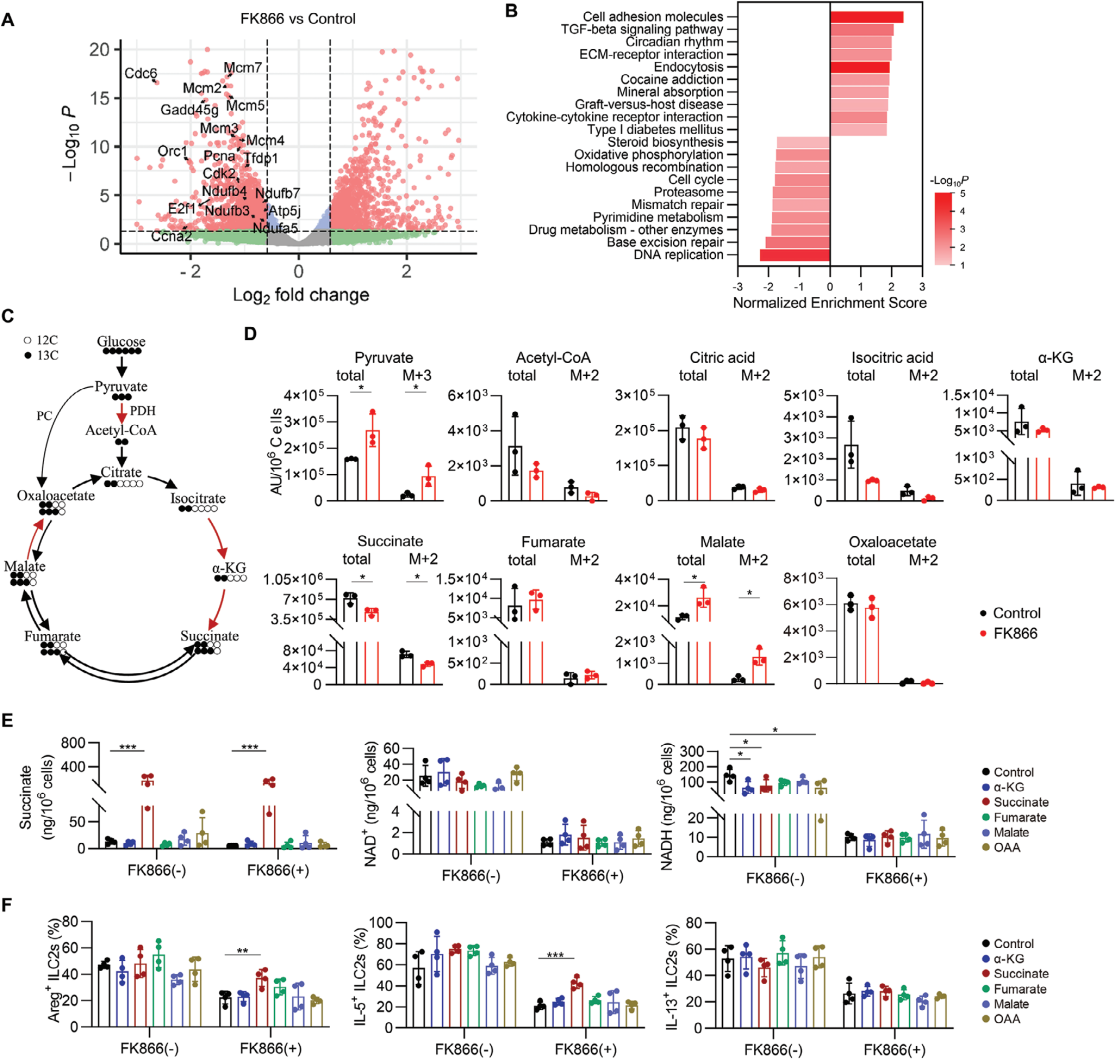
NAD^+^ sustains intestinal ILC2s by supporting succinate. A,B) Sorted large intestinal ILC2s were treated with FK866 for 16 h and then subjected to RNA‐seq. Volcano chart (A) and KEGG pathway analysis (B) for differently expression genes between control and FK866‐treated ILC2s.C,D) Sorted LI ILC2s were cultured in the presence of IL‐2, IL‐7, IL‐25, and IL‐33 for 5 days, followed by ^13^C_6_‐glucose and FK866 treatment for 24 h. Schematic depiction of the contribution of ^13^C_6_‐glucose to TCA cycle intermediates (C). White circles indicate ^12^C‐, and black circles indicate ^13^C‐atoms from glucose. The abundance of total pool size and ^13^C‐derived TCA cycle intermediates in ILC2s (D). Data were presented as mean ± SD; n = 3 per group. E) Sorted LI ILC2s were cultured in the presence of IL‐2, IL‐7, IL‐25, and IL‐33 for 5 days, followed by the indicated treatment for 24 h. Levels of succinate, NAD^+^ and NADH, in ILC2s treated with ɑ‐ketoglutarate (ɑ‐KG, 5 mm), succinate (10 mm), fumarate (5 mm), malate (10 mm), and oxaloacetate (OAA, 10 mm), with or without FK866 (200 nM). Data were presented as mean ± SD; n = 4 per group, from two independent experiments. F) Sorted LI ILC2s were cultured with ɑ‐KG (5 mm), succinate (10 mm), fumarate (5 mm), malate (10 mm), and OAA (10 mm), with or without FK866 (10 nM) for 16 h. The frequency of Areg, IL‐5, and IL‐13 in ILC2s (CD45.2^+^Lin^–^GATA3^+^). Data were presented as mean ± SD; n = 4 per group, from two independent experiments. The two‐sided unpaired t‐test or two‐sided Mann–Whitney U‐test according to normal distribution in D. The One‐way ANOVA with Dunnett or Kruskal Wallis with Dunns according to normal distribution in E and F. The statistical test method and exact *P* value are detailed in the Table S4 (Supporting Information). ^*^ *p* < 0.05, ^**^ *p* < 0.01, ^***^ *p* < 0.001.

NAD^+^ accepts high‐energy electrons from glycolysis and TCA intermediates, and ultimately feeds electrons into complex I of the ETC to drive oxidative phosphorylation, the primary source of adenosine triphosphate. Transcriptional profiles of ILC2s by SMART sequencing revealed that 1182 genes were upregulated and 1864 genes were downregulated in the FK866‐treated ILC2s (**Figure** **5**A and q value < 0.05, fold change > 1.5), with which gene set enrichment analysis showed downregulation of cell cycle and oxidative phosphorylation pathways (Figure 5B; Figure S5A, Supporting Information). Metabolomics of ILC2s showed that IL‐33 treatment elevated the concentrations of NAD^+^, citrate, isocitrate, succinate, fumarate, and malate in ILC2s, indicating that the activation of ILC2s by IL‐33 might require NAD^+^ to promote TCA cycle and fuel oxidative phosphorylation (GSE166081)^[^ ^15^ ^]^ (Figure S5B, Supporting Information).

We next sought to evaluate whether NAD^+^ regulates ILC2s through the metabolites of TCA cycle. Metabolic flow of glycolytic pathway and TCA cycle of ILC2s was analyzed after incubation with ^13^C_6_‐glucose for 24 h in the presence or absence of FK866 (Figure 5C,D; Figure S5C, Supporting Information). In each round of the TCA cycle, three molecules of NAD^+^ are reduced to NADH (Figure 5C). Pyruvate contributed two carbons to the TCA cycle through pyruvate dehydrogenase (PDH)‐dependent generation of acetyl coenzyme A (acetyl‐CoA), in which NAD^+^ was reduced to NADH (Figure 5C). FK866 treatment accumulated more pyruvate and malate than those of control group in ILC2s (Figure 5D). Of note, FK866 decreased the ^13^C‐labeled isotopologue of succinate was mainly derived from PDH (M+2), as well as total pool size of succinate (Figure 5D). Additionally, FK866 led to decreased succinate, NAD^+^, and NADH in ILC2s (Figure 5E; Figure S5D, Supporting Information), while TCA cycle supplementation had no impact on NAD^+^ and NADH in ILC2s with or without FK866 (Figure 5E; Figure S5D, Supporting Information). Accordingly, only succinate among the key TCA cycle metabolites rescued the function of FK866‐supressed ILC2s partially (Figure 5F), suggesting that NAD^+^ maintained ILC2s via supporting the generation of succinate.

**Figure 6:**
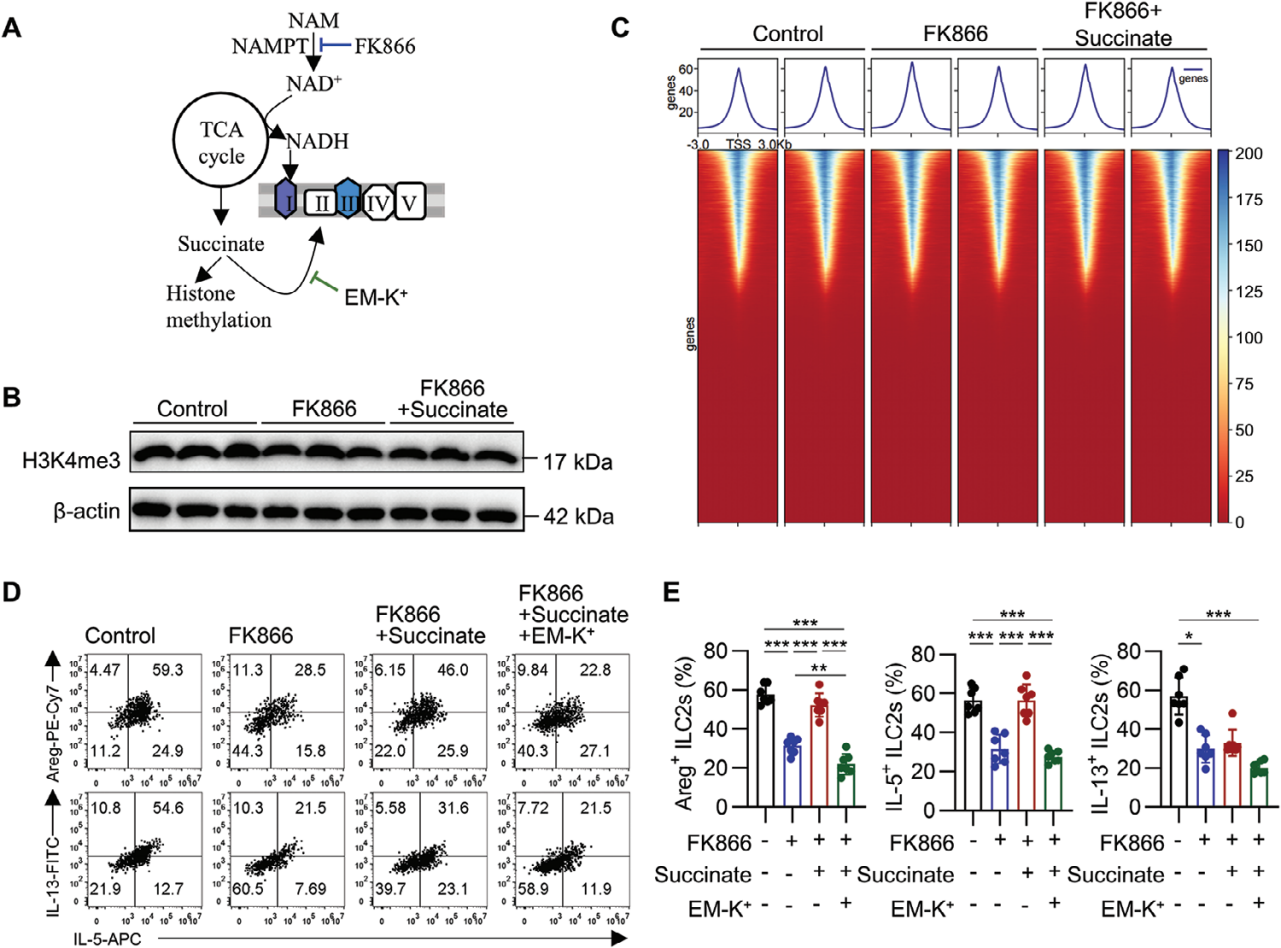
NAD^+^ maintains intestinal ILC2s via succinate‐fed ETC. A) Schematic illustration of the inhibitory effects of FK866 on nicotinamide phosphoribosyltransferase (NAMPT) or ethyl potassium malonate (EM‐K^+^) on succinate dehydrogenase (SDH). B) Sorted large intestinal ILC2s were cultured in the presence of IL‐2, IL‐7, IL‐25, and IL‐33 for 5 days, followed by the indicated treatment for 24 h. Representative western blotting of H3K4me3 in ILC2s under indicated treatment. C) Sorted large intestinal ILC2s were cultured in the presence of IL‐2, IL‐7, IL‐25, and IL‐33 for 5 days, followed by the indicated treatment for 24 h. CUT&Tag targeting H3K4me3 was conducted, and the H3K4me3 enrichment signal across all peak locations among different groups were shown. D,E) Representative FACS analyses (D) and frequency (E) of Areg, IL‐5, and IL‐13 expression in ILC2s treated with FK866 (10 nm), succinate (10 mm), or EM‐K^+^ (20 mm) for 16 h. Data were presented as mean ± SD; n = 7 per group, from three independent experiments. The One‐way ANOVA with Tukey or Kruskal Wallis with Dunns according to normal distribution in E. The statistical test method and exact *P* value are detailed in the Table S4 (Supporting Information). ^*^ *p* < 0.05, ^**^ *p* < 0.01, ^***^ *p* < 0.001.

As previously indicated, succinate can serve as a hydrogen ion provider to complex II, bypassing complex I to promote the ETC, or act as a suppressor of histone demethylation (**Figure** **6**A).^[^ ^27^ ^]^ To explore the impact of reduced succinate levels on histone methylation under conditions of disrupted NAD^+^ metabolism, we selected H3K4me3 as our target due to its established association with succinate^[^ ^28^, ^29^, ^30^ ^]^ and its influence on ILC2s function.^[^ ^15^ ^]^ Western blot analysis confirmed that H3K4me3 levels remained unchanged after NAMPT inhibition (Figure 6B). Furthermore, CUT&Tag genomic profiling revealed no significant difference in global H3K4me3 landscape upon FK866 treatment (Figure 6C), as well as the H3K4me3 enrichment at the loci of ILC2‐associated genes, such as *Gata3*, *Areg*, *Il5*, and *Il13* (Figure S5E, Supporting Information). To elucidate the role of succinate in the ETC of ILC2s, ethyl potassium malonate (EM‐K^+^), which impedes electron transport from succinate to the ETC,^[^ ^31^ ^]^ was administered in conjunction with succinate to FK866‐treated ILC2s. The results demonstrated that EM‐K^+^ abolished the restorative effects of succinate on FK866‐treated ILC2s (Figure 6D,E). Collectively, these findings suggest that NAD^+^ sustains the ETC via succinate generated from the TCA cycle, thereby preserving the function of ILC2s.

### 2.6 NAD^+^ Regulates gut ILC2s in a Cell‐Intrinsic Manner

**Figure 7:**
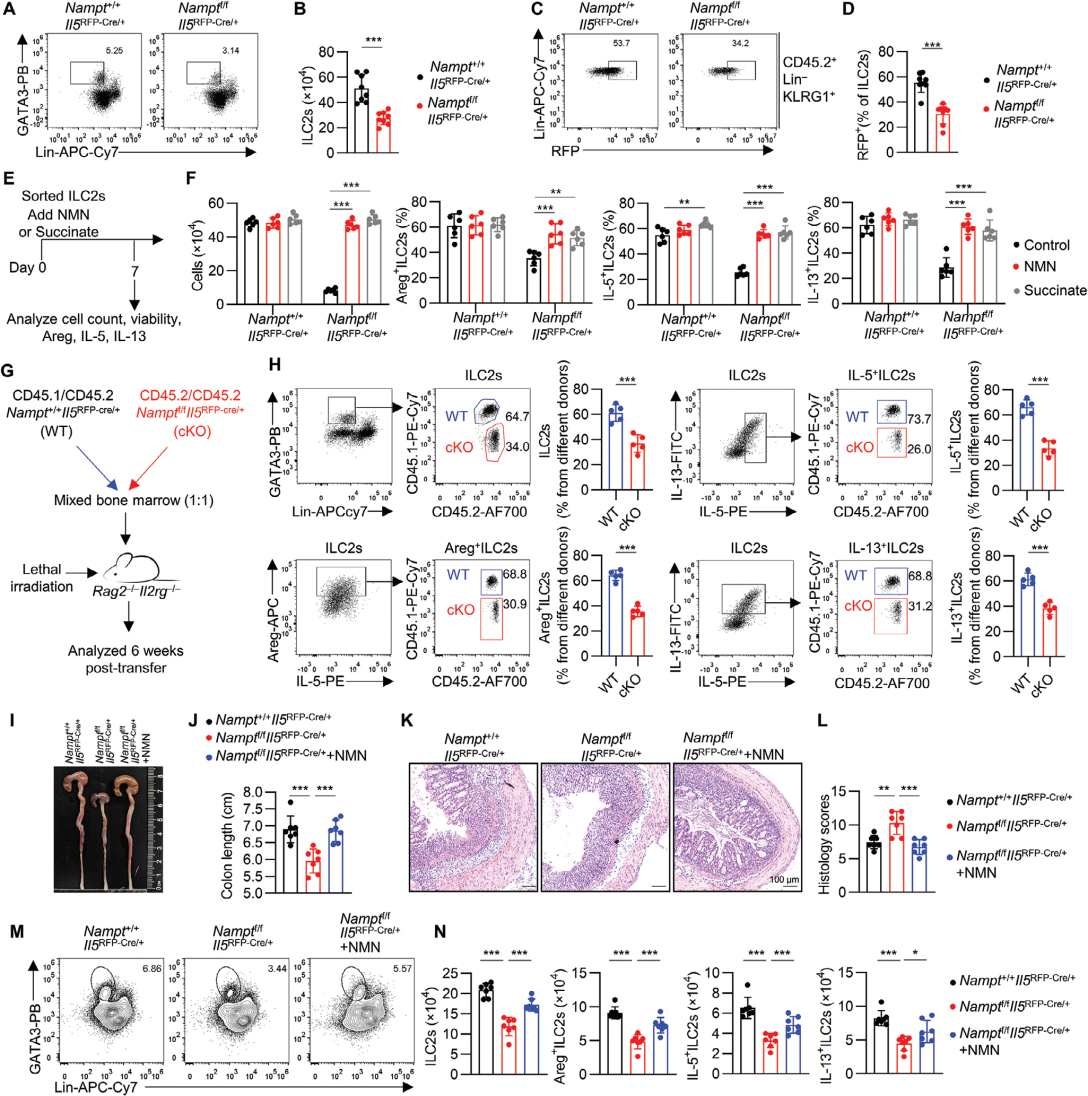
NAMPT deficiency in ILC2s aggravates DSS‐induced colitis. A,B) Representative FACS analyses and absolute numbers of ILC2s in the large intestine of *Nampt* ^+/+^ *Il5* ^RFP‐Cre/+^ or *Nampt* ^f/f^ *Il5* ^RFP‐Cre/+^ littermate mice. Data were presented as mean ± SD; n = 8 per group, from four independent experiments. C,D) Representative FACS analyses and frequency of RFP^+^ ILC2s after gating on Lin^–^ KLRG1^+^ ILC2s in the LI of *Nampt* ^+/+^ *Il5* ^RFP‐Cre/+^ or *Nampt* ^f/f^ *Il5* ^RFP‐Cre/+^ littermate mice; mean ± SD of 8 biological replicates; from three independent experiments. E,F) Large intestinal ILC2s were sorted from *Nampt* ^+/+^ *Il5* ^RFP‐Cre/+^ or *Nampt* ^f/f^ *Il5* ^RFP‐Cre/+^ littermate mice and cultured in the presence of IL‐2, IL‐7, IL‐25, and IL‐33 for 7 days. The nicotinamide mononucleotide (NMN) or succinate were added on day 0, and ILC2s were collected at day 7. Experimental strategy (E). The absolute numbers of ILC2s and frequency of Areg, IL‐5, and IL‐13 expression in ILC2s (CD45.2^+^Lin^–^GATA3^+^) (F). Data were presented as mean ± SD; n = 6 per group, from three independent experiments. G,H) The lethally irradiated *Rag2* ^–/–^ *Il2rg* ^–/–^ recipient mice were transferred with an equal mixture (1:1) of bone marrow cells from *Nampt* ^+/+^ *Il5* ^RFP‐Cre/+^ (WT, CD45.1/CD45.2) or *Nampt* ^f/f^ *Il5* ^RFP‐Cre/+^ (cKO, CD45.2/CD45.2) mice. Experimental strategy (G). Representative FACS analyses and frequency of ILC2s, Areg^+^ILC2s, IL‐5^+^ILC2s, and IL‐13^+^ILC2s from indicated donors (H). Data were presented as mean ± SD; n = 5 per group, from two independent experiments. I–N) The *Nampt* ^f/f^ *Il5* ^RFP‐Cre/+^ mice were orally administrated NMN for two weeks before DSS administration. Representative colons (I) and colon lengths (J), representative hematoxylin‐eosin (H&E) staining (K) and histological severity scores of colons (L). Flow cytometry analysis of Lin^–^GATA3^+^ ILC2s expression (M), absolute numbers of ILC2s, Areg^+^, IL‐5^+^ and IL‐13^+^ ILC2s (N); mean ± SD of 7 biological replicates, from two independent experiments. The two‐sided unpaired t‐test in B, D, H, J, L, N. The One‐way ANOVA with Dunnett in F. The statistical test method and exact *P* value are detailed in the Table S4 (Supporting Information). ^*^ *p* < 0.05, ^**^ *p* < 0.01, ^***^ *p* < 0.001.

To verify the regulation of ILC2s by NAD^+^ in a genetic approach, we generated *Nampt* ^f/f^ *Il5* ^RFP‐Cre^ mice to specifically delete *Nampt* in ILC2s, since IL‐5 is mainly produced by ILC2s in the gut (Figure S6A,B, Supporting Information), and thus *Il5* ^RFP‐cre^ mice is used to eliminate genes in ILC2s. Decreased ILC2s, as well as Areg^+^, IL‐5^+^ and IL‐13^+^ ILC2s, were observed in the LI of *Nampt* ^f/f^ *Il5* ^RFP‐Cre^ mice compared to littermate *Nampt* ^+/+^ *Il5* ^RFP‐Cre^ mice (**Figure** **7**A,B; Figure S6C,D, Supporting Information). Of note, ex vivo ILC2s from *Nampt* ^f/f^ *Il5* ^RFP‐Cre^ mice only showed reduced RFP proportion in ILC2s, but not IL‐5 and IL‐13 (Figure 7C,D; Figure S6E, Supporting Information), which might be owing to the elimination of *Nampt*‐knockout ILC2s, and thus more *Nampt*‐intact ILC2s existed in the gut of *Nampt* ^f/f^ *Il5* ^RFP‐Cre^ mice. To this end, the LI ILC2s were sorted from *Nampt* ^+/+^ *Il5* ^RFP‐Cre^ and *Nampt* ^f/f^ *Il5* ^RFP‐Cre^ mice and cultured in vitro in the presence of IL‐2, IL‐7, IL‐25, and IL‐33 to enhance IL‐5 expression and improve the knockout efficiency (Figure 7E). The number, viability, and effector molecules production of ILC2s isolated from *Nampt* ^f/f^ *Il5* ^RFP‐Cre^ mice were significantly lower than those of littermate controls after seven‐day culture in vitro (Figure 7F). The reduced viability and function of ILC2s lack of *Nampt* were restored by NMN or succinate treatment, in line with the electron feeding to ETC by NAD^+^ through succinate to sustain ILC2s in the gut (Figure 7F).

To further corroborate the cell‐intrinsic role of NAMPT in ILC2s, we conducted a mixed‐bone‐marrow transfer experiment. Bone marrow cells isolated from *Nampt* ^+/+^ *Il5* ^RFP‐Cre^ (CD45.1/CD45.2) and *Nampt* ^f/f^ *Il5* ^RFP‐Cre^ (CD45.2/CD45.2) mice were mixed at a 1:1 ratio and transferred into *Rag2* ^–/–^ *Il2rg* ^–/–^ mice (Figure 7G). Six weeks post transfer, ILC2s were isolated from the LI of the bone‐marrow‐chimeric mice and distinguished with congenic markers to trace their origins. Less LI ILC2s, as well as Areg‐, IL‐5‐, and IL‐13‐producing ILC2s, were derived from *Nampt* ^f/f^ *Il5* ^RFP‐Cre^ bone marrow (Figure 7H). Collectively, these data demonstrate that NAD^+^ regulates gut ILC2s in a cell‐intrinsic manner.

### 2.7 NAMPT Deficiency in ILC2s Aggravates DSS‐Induced Colitis

We next sought to investigate the impact of NAMPT deficiency in ILC2s on DSS‐induced colitis (Figure 7I‐N). *Nampt* ^f/f^ *Il5* ^RFP‐Cre^ mice and littermate controls were fed with DSS, and more severe colitis was observed in *Nampt* ^f/f^ *Il5* ^RFP‐Cre^ mice in terms of shortened colon, increased histological severity, which was mitigated by NMN supplementation (Figure 7I‐L).

Correspondingly, the number of ILC2s, as well as Areg, IL‐5 and IL‐13 production by ILC2s, were diminished in the LI of *Nampt* ^f/f^ *Il5* ^RFP‐Cre^ mice compared to those of control mice after DSS treatment, which was restored by NMN (Figure 7M,N). These results suggested that intact NAD^+^ metabolism in ILC2s is indispensable to confine the progression of colitis.

## 3 Discussion

Liver injury causes various metabolic dysfunction and dysbiosis that may contribute to increased disease activity and mortality of IBD. For example, the high incidence of IBD in PSC patients has been noticed clinically.^[^ ^32^ ^]^ However, the mechanism underlying the exacerbation of IBD by liver disease remains elusive. In this study, we found that UC patients complicated with liver injury had more severe disease revealed by higher UCEIS scores, which was reproduced in mice that DSS‐induced colitis was aggravated by chemical‐ or non‐chemical‐based liver cirrhosis. NAM acts as the NAD^+^ precursor and is the product of NAD^+^ degradation in the liver reciprocally.^[^ ^5^ ^]^ Accordingly, liver injury‐related disruption of NAD^+^ metabolism was associated with this phenomenon. Additionally, we also noticed reduced L‐serine, pantothenic acid, and retinol, and enriched deoxycholic acid, 3‐glucuronide and L‐tyrosine in the plasma of patients with liver cirrhosis. The changes of these metabolites may participate in the UC progression since serine and pantothenic acid relieve DSS‐induced colitis, and retinoic acid maintains immune homeostasis in the gut.^[^ ^33^, ^34^, ^35^ ^]^ The role of deoxycholic acid is contradictory as exacerbating enteritis by promoting M1 polarization or alleviating DSS‐induced colitis through maintaining epithelial integrity.^[^ ^36^, ^37^ ^]^ However, the action of L‐tyrosine in colitis needs further investigation.

The role of NAD^+^ metabolism in colitis remains controversial. Deletion of NAMPT, the key enzyme in the NAD^+^ salvage synthesis pathway, in the myeloid cell lineage reduces phagocytic activity of macrophages and exacerbates DSS‐induced colitis.^[^ ^38^ ^]^ In the contrary, NAMPT inhibition by intraperitoneal injection of FK866 promotes the transformation of M1 to M2 and alleviates colitis in mice.^[^ ^6^ ^]^ In this study, we found that specific deletion of NAMPT in ILC2s interfered their function in tissue repair and aggravated DSS‐induced colitis. However, the administration of FK866 in vivo elevated ILC2s function in the gut (data not shown). This phenomenon is probably due to increased IL‐33 indirectly by suppressing epithelial production of glucagon‐like peptide‐1, which is controlled by NAMPT and negatively regulates IL‐33 release.^[^ ^39^, ^40^ ^]^ Notably, the inhibition of NAMPT in vivo by FK866 injection lasted for 5 days, while the treatment of CCl4 continued for 6 weeks. Although both of two conditions cause impaired NAD^+^ metabolism, different duration of NAD^+^ deficiency may lead to diverse responses of different cell compartments, and thus show opposite impact on gut ILC2s.

Sufficient NAD^+^ is indispensable for the cells’ energy production pathways, including glycolysis, TCA cycle, and oxidative phosphorylation. It is reported that the diminished NAD^+^ level by NAMPT inhibition impairs the generation of several intermediates of the TCA cycle, such as *α*‐KG, malate, and succinate, in multiple cell types.^[^ ^41^, ^42^ ^]^ However, we noticed that only replenishment of succinate, but not other key metabolites in TCA cycle, rescued ILC2s function after pharmacological inhibition or genetic ablation of NAMPT, suggesting succinate‐fed ETC, instead of NAD^+^‐regulated epigenetic machinery, is crucial for ILC2s.

In this study, we find that impaired NAD^+^ metabolism caused by liver diseases diminishes the viability and function of ILC2s in the gut. However, the effect of other changed metabolites in the plasma of cirrhosis patients on ILC2s cannot be excluded. Additionally, DSS feeding is performed after liver injury in our study, but the impact of the occurrence of liver injury after the onset of IBD needs further investigation. Mechanistically, given that succinate rescues IL‐5 and Areg, but not IL‐13 expression in FK866‐treated ILC2s, there might be other mechanisms responsible for IL‐13 production in ILC2s other than succinate‐fed mitochondrial respiration. Clinically, it needs to be further validated whether ILC2s function is compromised in liver disease‐complicated IBD patients.

## 4 Experimental Section

### Mice

Male and female C57BL/6J mice at the age of 6–8 weeks old were used for all animal experiments. C57BL/6 WT mice and *Nampt* ^f/f^ mice were purchased from GemPharmatech. *Rag2* ^–/–^ *Il2rg* ^–/–^ mice were purchased from Taconics Biosciences. *Il5* ^RFP‐Cre^ mice and CD45.1/CD45.1 mice were purchased from Jackson Laboratories. The *Nampt* ^f/f^ mice were crossed to *Il5* ^RFP‐Cre^ mice to obtain a strain with *Nampt* ^f/f^ *Il5* ^RFP‐Cre^ conditional gene deletion and *Nampt* ^+/+^ *Il5* ^RFP‐Cre^ WT littermates. All the mice in this study were housed in specific‐pathogen‐free facilities in ventilated cages with ad libitum food and water at the Shandong University.

### Clinical Sample Acquisition

For retrospective chart review, the UC patients complicated with liver injury diagnosed at Qilu hospital between January 2019 and June 2024 and age‐ and sex‐ matched UC patients without liver injury controls at similar visits time were used. The diagnosis of UC was based on well‐established clinical, endoscopic, and histopathological criteria by experienced physicians. Liver injury was defined as ALT and/or AST higher than the upper limit of normal (ULN). ULN of ALT and AST were referred to the criteria of The Asian Pacific Association for the Study of the Liver (40 U L^−1^ for both genders).^[^ ^43^ ^]^ Liver injury grade I was defined as 1 × ULN (40 U L^−1^) < ALT and/or AST < 2 × ULN (80 U L^−1^), and liver injury grade II was defined as ALT and/or AST ≥ 2 × ULN (80 U L^−1^). Exclusion criteria of UC patients were as follows: had history of diabetes; combined with Crohn's disease, malignant tumor. The characteristics of UC patients with or without liver injury were listed in Tables S1 and S2 (Supporting Information).

Human blood and mucosal biopsies were obtained at Qilu hospital of Shandong University. The human blood samples were obtained from 40 healthy donors (30 for metabolic analysis and 10 for flow cytometry analysis) and 40 patients with liver cirrhosis (30 for metabolic analysis and 10 for flow cytometry analysis). The mucosal biopsies samples were obtained from five healthy donors. Exclusion criteria of healthy donors and liver cirrhosis were as follows: had history of diabetes; combined with malignant tumor. In addition, the healthy donors had no history of alcohol abuse or viral hepatitis. The characteristics of healthy donors and liver cirrhosis patients were listed in Table S3 (Supporting Information).

### CCl4‐Induced Liver Cirrhosis

The mice were intraperitoneal injection with CCl4 (2 mL kg^−1^) dissolved in olive oil at a ratio of 1:4, twice a week for 6 weeks. Olive oil was used as a vehicle control.

### BDL Surgery

The mice aged 12 weeks were anaesthetized with 3.5 µL g^−1^ of 2% sodium pentobarbital. After skin disinfection, a midline abdominal incision was made. The common bile ducts were exposed and ligated using 5 – 0 non‐absorbable sutures.

### DSS‐Induced Colitis

The mice were administrated with 3% (w/v) DSS (MP Biomedicals) in their drinking water for 5 consecutive days. DSS‐treated mice were monitored daily for body weight and percentages of weight change were determined. Colon length was measured. The middle part of the colon tissue was collected and fixed in neutral buffered 4% formalin. Samples were dehydrated, embedded in paraffin, sectioned at 4 µm, and stained with H&E. Histology scores were blindly analyzed by a trained gastrointestinal pathologist according to a previously described standard. In brief, the histological scores include severity of inflammation (1 < 10%, 2 = 10–25%, 3 = 26–50%, 4 >51%), extent of inflammation (1 = mucosal, 2 = mucosal and submucosal, 3 = mucosal, submucosal, and transmural), extent of crypt damage (1 = basal 1/3 damaged, 2 = basal 2/3 damaged, 3 = only surface epithelium intact, 4 = entire crypt and epithelium lost), percent of crypt damage (1 = 1–25%, 2 = 26–50%, 3 = 51–75%, 4 = 76–100%). The histology score was the sum of the four sections scores.

### Immunohistochemistry Staining

Formaldehyde‐fixed, paraffin‐embedded slides were subjected to dewaxing, hydration and antigen retrieval, followed by blocking and antibody incubation. Primary antibodies were diluted as suggested, added onto slides and were incubated at 4 °C overnight in a moist chamber. Then slides were washed with PBS and incubated with HRP‐conjugated secondary antibodies.

### scRNA‐seq

The colon tissues were enzymatically digested into single‐cell suspensions using Multi Tissue Dissociation Kits (Miltenyi Biotec). The single‐cell suspensions were converted to barcoded scRNA‐seq libraries through steps including droplet encapsulation, emulsion breakage, mRNA captured bead collection, reverse transcription, cDNA amplification, and purification. Qualification was performed using Qubit ssDNA Assay Kit (Thermo Fisher Scientific) and Agilent Bioanalyzer 2100. All libraries were further sequenced by the DNBSEQ‐T1 sequencing platform.

Sequencing data filtered and gene expression matrix was obtained using DNBelab C Series scRNA analysis‐software (https://github.com/MGI‐tech‐bioinformatics/DNBelab_C_Series_HT_scRNA‐analysis‐software). Briefly, all samples were performed sample de‐multiplexing, barcode processing, and single‐cell 3′ unique molecular identifier (UMI) counting with default parameters. Processed reads were then aligned to GRCh38 genome reference using STAR (v2.5.3). Valid cells were automatically identified based on the UMI number distribution of each cell by using the “barcodeRanks” function of the DropletUtils tool to remove background beads and the beads that had UMI counts less than the threshold value. Finally, PISA was used to calculate the gene expression of cells and create a gene x cell matrix for each library.

Scanpy (version 1.8.1)^[^ ^44^ ^]^ and Seurat (4.0.1)^[^ ^45^ ^]^ analysis pipelines were applied to analyze our expression data. Briefly, only cells expressing at least 200 genes and genes containing non‐zero values as well as associated with at least 3 cells were maintained for subsequent analysis. “scanpy.pp.normalize_total” and “scanpy.pp.log1p” functions were used to normalize data and change to log counts. Highly variable genes were observed using the “scanpy.pp.highly_variable_genes” function with setting parameters min_mean = 0.0125, max_mean = 3, min_disp = 0.5, and these highly variable genes were used to perform principal components analysis (PCA). Principal components were selected by “sc.pl.pca_variance_ratio” function. These principal components were used to compute the neighborhood graph of cells and then were used to perform uniform manifold approximation and projection (UMAP) (https://doi.org/10.48550/arXiv.1802.03426) analysis using functions “scanpy.pp.neighbors” and “scanpy.tl.umap”. Leiden graph‐clustering method^[^ ^46^ ^]^ was used to cluster the neighborhood graph of the cells. The marker genes of each cluster were identified using the “scanpy.tl.rank_genes_groups” function.

### Isolation of Immune Cells from Intestinal Lamina Propria

The isolation of mouse immune cells from intestinal lamina propria was done as previously described.^[^ ^47^ ^]^ Briefly, large intestines were separated and fat tissues were removed. Intestines were cut open and washed in PBS, and were then cut into 1 cm‐long pieces, washed and shaken in PBS for 2 min. Intestines were incubated in PBS containing 30 mm EDTA and 10 mm HEPEs with shaking 200 rpm at 37 °C for 30 min. The tissues were then digested in RPMI1640 containing FBS (5%), 1% penicillin‐streptomycin, DNase I (150 µg mL^−1^, Sigma) and collagenase VIII (150 U mL^−1^, Sigma) at 37 °C in 5% CO_2_ incubator for 1.5 h. The digested tissues were shaken and filtered through 100 µm cell strainers. Mononuclear cells were then harvested from the interphase of an 80% and 40% Percoll (Cytiva) gradient after a spin at 2500 rpm for 15 min at room temperature.

For human mucosa, mucosal tissues were washed twice in PBS. Mucosa were incubated in PBS containing 10 mm EDTA (Solarbio) and 5% FBS (Gibco) with shaking 220 rpm at 37 °C for 20 min. The tissues were then digested in RPMI1640 (Macgene) containing FBS (5%, Gibco), 1% penicillin‐streptomycin (Macgene), DNase I (150 µg mL^−1^, Sigma) and collagenase IV (5 mg mL^−1^, Sigma) with shaking 80 rpm at 37 °C for 30 min. The digested tissues were shaken and filtered through 100 µm cell strainers. Mononuclear cells were then harvested at 2500 rpm for 5 min at room temperature.

### Flow Cytometry and Cell Sorting

For cytokine production, cells were stimulated ex vivo by 50 ng mL^−1^ PMA (PeproTech) and 500 ng mL^−1^ ionomycin (BioGems) for 4 h. Brefeldin A (2 µg mL^−1^, BioGems) was added 2 h before cells were harvested for analysis. The live and dead cells were discriminated by Zombie Aqua Fixable Viability Kit (BioLegend) in PBS. CD16/32 antibody was used to block the non‐specific binding to Fc receptors before surface staining. Cells were stained with surface‐labeled antibodies for 25 min at 4 °C. For intracellular staining, cells were incubated with Fixation/Permeabilization at 4 °C overnight. After fixation, the cells were incubated with the indicated antibodies for 2 h at 4 °C. Flow cytometry was performed with Backman Gallios cytometers or FACSymphony A3 and analyzed with FlowJo software. For sorting experiments, ILC2s were sorted from large intestinal lamina propria using a Backman Moflo Astrios EQ. For flow cytometry analyses, live cells were gated after singlet discrimination. Mouse ILC2 cells were gated based on the following markers: Lin^–^CD127^+^KLRG1^+^, CD45.2^+^Lin^–^Gata3^+^, or CD45.2^+^Lin^–^KLRG1^+^ (Figure S7, Supporting Information). Antibodies used for flow cytometry were shown in Table S5 (Supporting Information).

### Adoptive Transfer of Intestine ILC2s into CCl4‐Treated Mice

For adoptive transfer, large intestinal ILC2s (Lin^–^CD127^+^KLRG1^+^) were sorted from WT mice. To obtain sufficient numbers of ILC2s, sorted ILC2s were cultured in IMDM media supplemented with 15% FBS, mIL‐2 (10 ng mL^−1^, PeproTech), mIL‐7 (10 ng mL^−1^, PeproTech), mIL‐25 (10 ng mL^−1^, PeproTech) and mIL‐33 (10 ng mL^−1^, PeproTech) for 5 days. In the last week of CCl4 model, a total of 2 × 10^6^ ILC2s were harvested and transferred to mice twice via tail vein injection.

### Metabolomics Analysis

For analysis of plasma metabolites, 100 µL aliquots were extracted using a 400 µL methanol: acetonitrile (1: 1, v/v) solution. The mixture sonicated at 40 kHz for 30 min at 5 °C, and then the samples were placed at −20 °C for 30 min to precipitate proteins. After centrifugation at 13 000 g at 4 °C for 15 min, the supernatants were carefully transferred to new microtubes and evaporated to dryness under a gentle stream of nitrogen. The samples were reconstituted in 100 µL loading solution of acetonitrile: water (1: 1, v/v) by brief sonication in a 5 °C‐water bath. Extracted metabolites were spun for 15 min at 13,000 g at 4 °C on a bench‐top centrifuge and cleared supernatant were transferred to sample vials for UPLC‐MS/MS analysis using UPLC‐Q Exactive system of Thermo Fisher Scientific.

After the mass spectrometry detection was completed, the raw data of LC/MS was preprocessed by Progenesis QI (Waters Corporation, Milford, USA) software, and a three‐dimensional data matrix in CSV format was exported. The information in this 3D matrix includes: sample information, metabolite name and mass spectral response intensity. The metabolites were searched and identified, and the main database was the HMDB (http://www.hmdb.ca/), Metlin (https://metlin.scripps.edu/) and Majorbio Database. The data after the database search was analyzed by the Majorbio cloud platform (https://cloud.majorbio.com).

### Long‐Term NMN Administration

Water consumption was measured for 1 week prior to the start of NMN administration. Mice were given NMN (Macklin) in drinking water at 1 g/kg/d, based on the previously measured water consumption. The NMN administration began at the start of CCl4 intraperitoneal injection.

### Measurements of NAD^+^ Metabolites and TCA Cycle Intermediates

Liver, plasma and intestinal NAD^+^ or NAM were determined using a HPLC‐MS system with an ADME column (100 mm × 2.1 mm). Frozen plasma samples were thawed at 25 °C and vortex‐mixed briefly. 100 µL plasma or ≈10 mg liver or colon was extracted in 1 mL acetonitrile, followed by additions of IS working solution (Oxiracetam, 20 µg mL^−1^, 10 µL) and methanol–water (5:95, v/v) (30 µL). The mixture was extracted by shocking at 4 °C for 6 min and then centrifuged at 10 000 g for 20 min. The supernatants were transferred to another 1.5 mL Eppendorf tube and evaporated to dryness at 30 °C by a vacuum extractor. The residue was reconstituted in 150 µL of 5 mm ammonium formate.

For cellular NAD^+^, NADH, and TCA cycle intermediates analysis, cells were extracted in 600 µL acetonitrile and, followed by additions of IS working solution (Oxiracetam, 10 µg mL^−1^, 10 µL, and ^13^C_4_ succinate, 1 µg mL^−1^, 10 µL) and 100 µL methanol–water (5:95, v/v). The extractions were centrifuged at 10 000 g for 20 min. The supernatants were divided into two portions and evaporated to dryness at 30 °C by a vacuum extractor. The first portion was reconstituted in 100 µL of 10 mm ammonium formate with 0.1% formate for NAD^+^ and NADH analysis. For TCA cycle intermediates analysis, the second portion was reconstituted in 20 µL of methanol (5%) solution and 20 µL of *N*‐(3‐dimethylaminopropyl)‐*N*′‐ethylcarbodiimide and 20 µL of 3‐nitrophenylhydrazine hydrochloride, and then incubated at room temperature for 60 min, afterwards 40 µL of 0.05 mg mL^−1^ 2,6‐di‐tert‐butyl‐4‐methylphenol were added to the samples and vortexed.^[^ ^48^ ^]^ Respective standards were used to generate standard calibration curves. Samples were analyzed on a Qtrap 5500 (AB Sciex) system. LI and liver metabolite concentrations were normalized to tissue weight. Cellular metabolites concentrations were normalized to cell counts.

### Administration of AAV‐shQprt in mice

AAV2/8‐TBG‐mir30‐*m*‐*Qprt*‐ZsGreen (*Qprt‐*KD) were constructed by Hanbio Biotechnology, and AAV2/8‐TBG‐ZsGreen was used as control. For AAV transduction, the viruses (1 × 10^11^ genomic copies/mouse) were delivered via tail vein injection to 6‐week‐old mice for 4 weeks, and then the transfected mice were subjected to DSS treatment or isolation of immune cells from intestinal lamina propria.

### Cell Culture

Immune cells from intestinal lamina propria were cultured in IMDM media (Macgene) supplemented with 5% FBS for 4 h, or in IMDM media supplemented with 10% FBS for 16 h. Sorted ILC2s were cultured in IMDM media supplemented with 15% FBS, mIL‐2 (10 ng mL^−1^, PeproTech), mIL‐7 (10 ng mL^−1^, PeproTech), mIL‐25 (10 ng mL^−1^, PeproTech) and mIL‐33 (10 ng mL^−1^, PeproTech) for indicated times. Human PBMC were cultured in RPMI1640 media supplemented with 5% FBS. Human LPMNCs were cultured in RPMI1640 media supplemented with 10% FBS, hIL‐2 (20 ng mL^−1^, PeproTech), hIL‐7 (20 ng mL^−1^, PeproTech), hIL‐25 (20 ng mL^−1^, PeproTech) and hIL‐33 (20 ng mL^−1^, PeproTech) for 16 h. All the media contains 1% penicillin/streptomycin, 0.1% gentamycin, and the cells maintained at 37 °C in 5% CO_2_ incubator.

### Bone Marrow Transfer

To generate mixed bone marrow chimeras, bone marrow cells from *Nampt* ^f/f^ *Il5^RFP‐Cre^* (CD45.2/CD45.2) mice were mixed at 1:1 ratio with *Nampt* ^+/+^ *Il5^RFP‐Cre^* (CD45.1/CD45.2) cells, and 5 × 10^6^ cell mixture were intravenously injected into *Rag2* ^–/–^ *Il2rg* ^–/–^ mice recipients irradiated at 700 rads. Chimeric mice were treated with antibiotics (sulfamethoxazole and trimethoprim suspension, Hi‐Tech Pharmacal) for 2 weeks after injection and were analyzed 6 weeks after reconstitution.

### Quantitative Real‐Time RT‐PCR

The liver or colon tissues were dissolved in RNA isolation reagent (Vazyme) and total RNA was isolated. cDNA was synthesized from extracted total‐RNA using Reverse Transcriptase kit (Vazyme) according to the manufacturer's protocol. Quantitative PCR was performed with SYBR‐Green premix (Vazyme) and detected by a Real Time PCR System (StepOne, Applied Biosystems). The expression levels of target gene were normalized to the housekeeping gene *Gapdh*. For analysis, 2^−∆∆Ct^ was used to calculate the relative mRNA expression of target genes. The primer sequences used for qRT‐PCR were shown in Table S6 (Supporting Information).

### RNA‐Seq and Analysis

LI ILC2s were sorted from WT and cultured in the presence of IL‐2, IL‐7, IL‐25, and IL‐33, and treated with or without FK866 for 16 h. ILC2s were lysed in RNA isolation reagent and total RNA was isolated. Full‐length cDNA was generated using SMART‐Seq HT Kit (Takara), paired‐end DNA libraries was generated and indexed using Nextera XT Library Prep Kit and then Nextera XT Index Kit. Barcoded samples were pooled and sequenced run with Illumina Novaseq TM 6000 sequence platform, generating 2 × 150 bp paired‐end reads. The reads were further filtered by Cutadapt (https://cutadapt.readthedocs.io/en/stable/, version: cutadapt‐1.9). Filtered reads were mapped against the mm10 assembly of the Mus musculus genome (National Center for Biotechnology Information) using hisat2 (Version 2.2.1). Significantly changed genes were identified by DE‐seq2. Significantly changed genes were used for pathway analysis with GSEA software.

### Stable Isotope Labeling and Metabolites Analysis

For ^13^C tracing experiments, ILC2s were cultured with glucose‐free IMDM containing 10% FBS, ^13^C_6_‐glucose (4.5 g L^−1^) in the presence or absence of FK866 for 24 h. At the conclusion of the tracer experiment, medium was aspirated, and cells were washed twice with PBS. The live viable cells were calculated. The 80% methanol was added to each tube followed by sonicate and centrifugation. The entire supernatant was divided into two portions, and evaporated to dryness under a gentle stream of nitrogen. The first portion was reconstituted in 100 µL of methanol (80%) solution for Glycolytic metabolites analysis. For TCA cycle intermediates analysis, the second portion was reconstituted in 20 µL of methanol (5%) solution and 20 µL of EDC and 20 µL of 3‐NPH, and then incubated at room temperature for 60 min. Samples were analyzed by UPLC‐MS using a UPLC HILIC column (4.6‐mm, Amide XBridge, Waters) installed in a Waters Acquity ultra performance liquid chromatography (UPLC) interfaced with Xevo tandem mass spectrometer (Metabo‐Profile Biotechnology Co., Ltd., Shanghai, China). Mass isotopomer were determined by integrating metabolite ion fragments. The metabolite levels were normalized by the total cell amount per sample.

### Western Blotting

Western blotting was performed as described previously. In brief, the cells were harvested and resuspended in 5xSDS‐PAGE loading buffer. The protein samples were then boiled and subjected to SDS‐PAGE, followed by immunoblotting with the appropriate primary antibodies and secondary antibodies.

### CUT&Tag and Data Processing

The CUT&Tag assay was conducted using Hrperactive Universal CUT&Tag Assay Kit for Immumina Pro (Vazyme, TD904). Briefly, 1.5 × 10^5^ ILC2s were collected and washed with 500 uL wash buffer before they were bound to activated ConA beads for 15 min at room temperature. Subsequently, H3K4me3 antibody‐beads was incubated at 4 °C overnight. Secondary antibody was added and incubated for 1 h at room temperature the next day. Then cells incubated with 0.04 µm pA/G‐Tnp for 1 h at room temperature. Next, sample was tagmentated in tagmentation buffer at 37 °C for one hour. The DNA was extracted and dissolved in 20 µL nuclease‐free water according to the manufacturer's instructions. Libraries were constructed using “TD904 Hyperactive Universal CUT&Tag Assay Kit for Illumina Pro” and “TD202 TruePrep Index Kit V2 for Illumina” from Vazyme Biotech.

The sequencing adapters were trimmed and read pairs with low quality or low complexity were filtered from raw data through Cutadapt (Version 2.5). Then, cleaned fastq data were mapped using Bowtie2 (Version 2.5.0) to the mm10 reference genome. Picard was used for marking and removing duplication. Samtools (Version 1.17) was used for converting and sorting the SAM files into BAM format. Macs2 (Version 2.2.7.1) was used for peak calling.

### Statistical Analysis

Statistical analyses were performed using GraphPad Prism (v.8). Normality for all datasets was assessed using the Shapiro–Wilk test, and subsequent parametric or non‐parametric tests were applied accordingly. Two‐sided unpaired t‐tests were employed for comparisons between two groups with a normal distribution, while two‐sided Mann–Whitney U‐tests were used for comparisons between two groups with a non‐normal distribution. For comparisons involving more than two groups, ANOVA was utilized with correction for multiple comparisons using Tukey or Dunnett for parametrically distributed datasets and the Kruskal–Wallis test with Dunn's correction for non‐parametrically distributed datasets. Statistically significant results were labeled (^*^ *p* < 0.05, ^**^ *p* < 0.01, ^***^ *p* <0.001). Error bars represent standard deviation unless otherwise indicated.

### Ethics Approval Statement

All studies with mice were approved by the Ethics Committee of Shandong University of Basic Medical Sciences (ECSBMSSDU2020‐2‐091). The human studies were performed according to the guidelines approved by the Ethics Committee of Shandong University of Basic Medical Sciences (ECSBMSSDU2020‐1‐053).

## Conflict of Interest

The authors declare no conflict of interest.

## Author Contributions

J.S., Z.L., X.L., and M.Z. contributed equally to this work. S.L., D.Y., and X.Z. designed and supervised the research. J.S., Z.L., X.L., and M.Z. performed most of the experiments. P.Z., Y.C., Q.T., Y.C., B.X., and Y.Z. provided help with experiments. Z.L., G.K., and W.L. carried out clinical sample collection and data analysis. W.T. formed the bioinformatics analysis of the RNA‐seq data and sc‐RNA seq data. X.G., J.Q., and C.L. provided help with animal models. S.L., J.S., and X.L. wrote the manuscript. Y.L., L.L., R.H., C.M., and Z.L. were involved in data discussions and edited the manuscript. All authors read and provided feedback on manuscript and figures.

## Supporting information

Supporting Information

## Data Availability

The data that support the findings of this study are available from the corresponding author upon reasonable request.
