# Supplementary material for: Sensing of Liver‐Derived Nicotinamide by Intestinal Group 2 Innate Lymphoid Cells Links Liver Cirrhosis and Ulcerative Colitis Susceptibility
Source: Adv Sci (Weinh). 2024 Aug 9;11(38):2404274. doi: 10.1002/advs.202404274 (PMC11481183; doi:10.1002/advs.202404274)
Supplement: Supplementary file 1 — Supporting Information [file ADVS-11-2404274-s001.pdf]

ADVANCED SCIENCE
Open Access

Supporting Information

for *Adv. Sci.*, DOI 10.1002/advs.202404274

Sensing of Liver-Derived Nicotinamide by Intestinal Group 2 Innate Lymphoid Cells Links Liver Cirrhosis and Ulcerative Colitis Susceptibility

Jing Shen, Zhen Li, Xiaoyu Liu, Mengqi Zheng, Peng Zhang, Yatai Chen, Qiuheng Tian, Wenyu Tian, Guanjun Kou, Yanyan Cui, Bowen Xu, Yunjiao Zhai, Weijia Li, Xiaohuan Guo, Ju Qiu, Chunyang Li, Ran He, Lixiang Li, Chunhong Ma, Yanqing Li, Xiuli Zuo, Detian Yuan* and Shiyang Li**

Sensing of Liver-Derived Nicotinamide by Intestinal Group 2 Innate Lymphoid Cells Links Liver Cirrhosis and Ulcerative Colitis Susceptibility

Jing Shen,† *Zhen Li,*† *Xiaoyu Liu,*†*Mengqi Zheng,*† *Peng Zhang, Yatai Chen, Qiuheng Tian, Wenyu Tian, Guanjun Kou, Yanyan Cui, Bowen Xu, Yunjiao Zhai, Weijia Li, Xiaohuan Guo, Ju Qiu, Chunyang Li, Ran He, Lixiang Li, Chunhong Ma, Yanqing Li, Xiuli Zuo,** *Detian Yuan,** *Shiyang Li.**

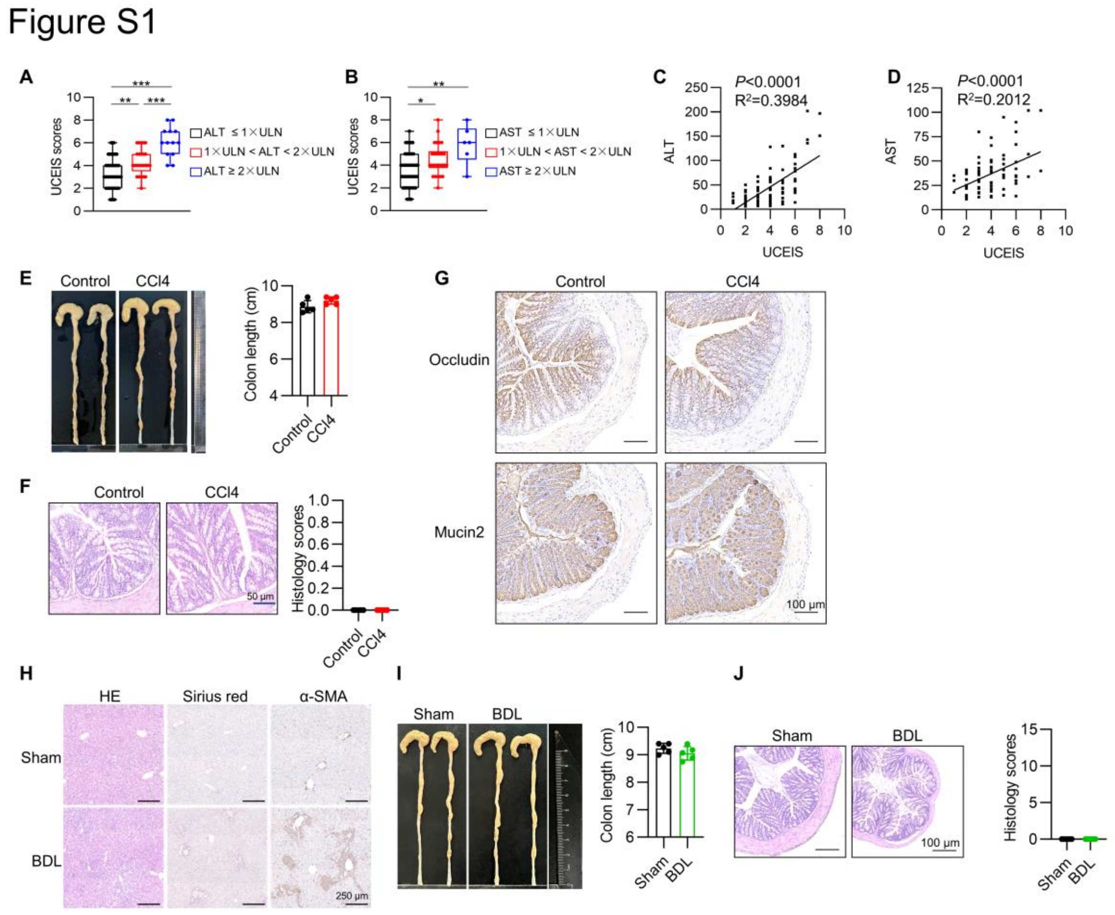

Figure S1. Liver injury does not cause intestinal inflammation and epithelial barrier alteration

(**A, B**) Ulcerative colitis endoscopic index of severity (UCEIS) scores in UC patients with higher alanine aminotransferase (ALT) or aspartate aminotransferase (AST). n=47 for ALT≤ 1×ULN group, n=25 for 1×ULN < ALT< 2×ULN group, and n=13 for ALT ≥ 2×ULN group

(A). n=53 for AST≤ 1×ULN group, n=26 for 1×ULN < AST< 2×ULN group, and n=6 for AST ≥ 2×ULN group (B). Box-and-whisker plot representing the median (line within the box), the interquartile range (length of the box), the min and the max (whiskers above and below the box) of UCEIS.

(**C, D**) Pearson's correlation between UCIES and ALT (C) or AST (D).

(**E-G**) Mice were intraperitoneally injected with carbon tetrachloride (CCl_4, 2 mL/kg, twice a week) for 6 weeks. Representative colons and colon lengths (E), representative hematoxylin-eosin (H&E) staining and histology scores of colons (F), immunohistochemistry analysis of Occludin and Mucin 2 of colons (G); mean±SD of 5 biological replicates, from 2 independent experiments.

(**H-J**) Mice were subjected to bile duct ligation (BDL) or sham operation. After a peirod of two weeks, H&E and Sirius Red staining, and alfa-smooth muscle actin immunohistochemistry staining of liver (H). Representative colons and colon lengths (I), representative H&E staining and histology scores of colons (J); mean±SD of 5 biological replicates, from 2 independent experiments.

The two-sided unpaired t-test in **A**, **B**, **E, I**. The statistical test method and exact *P* value are detailed in the **Table S4, Supporting Information**. *p<0.05, **p<0.01, ***p<0.001.

Figure S2

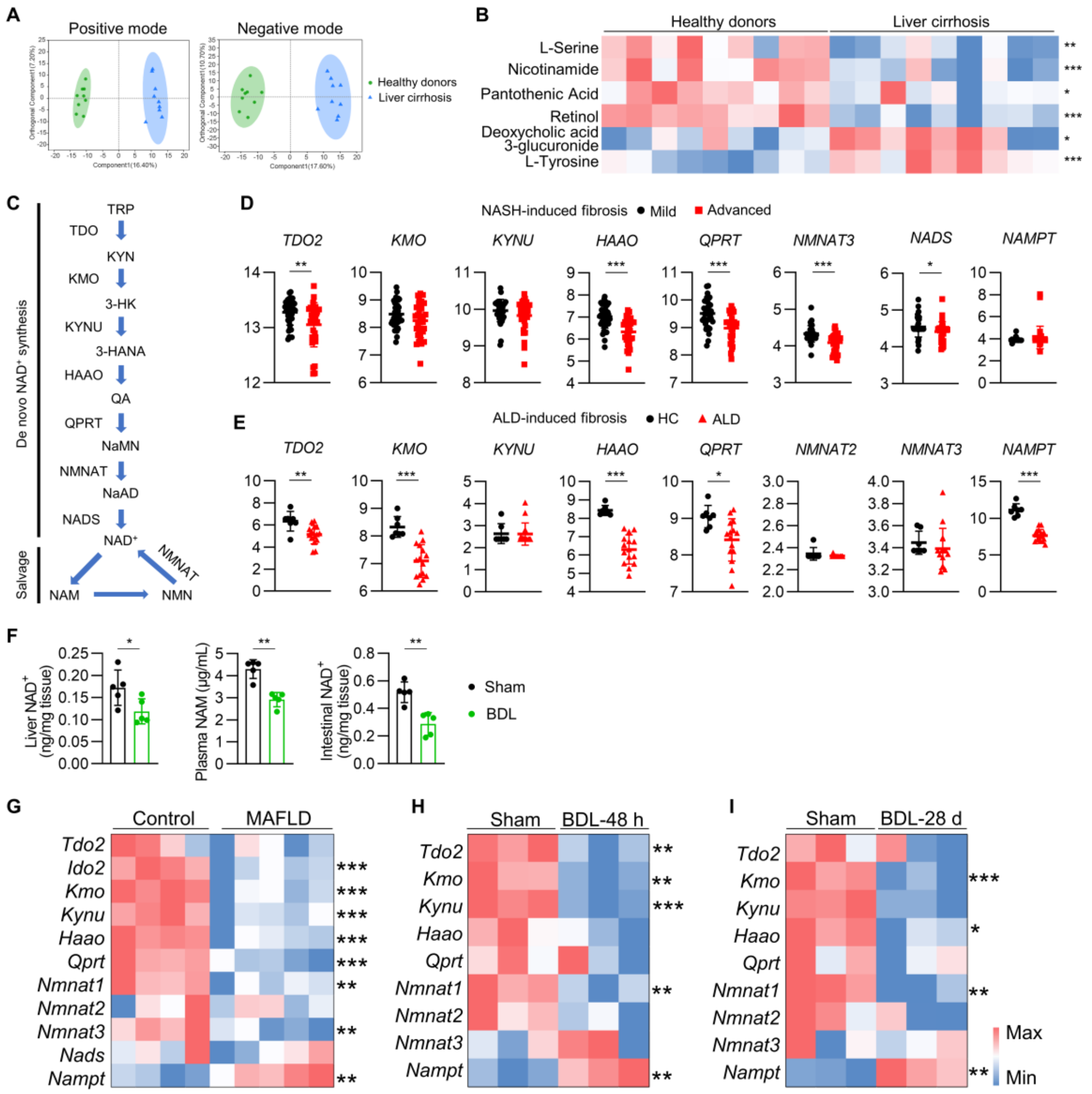

Figure S2. Liver cirrhosis disturbs NAD$^+$ metabolism in human and mice

(**A, B**) Metabolomics analysis of healthy donors and liver cirrhosis patients' plasma. Orthogonal projections to latent structures discriminant analysis (OPLS-DA) models for plasma samples from healthy donors and liver cirrhosis patients group in positive (A, left) and negative (A, right) ion mode. Heatmap revealed the plasma levels of L-serine, nicotinamide, pantothenic acid, retinol, deoxycholic acid 3-glucuronide, and L-tyrosine from biosynthesis of co-factor pathway in healthy donors and liver cirrhosis patients (B). n=9 per group.

(**C**) Schematic representation of the NAD$^+$ synthesis pathway. TRP, tryptophan; KYN, Kynurenine; 3-HK, 3-hydroxykynurenine; 3-HANA, 3-hydroxyanthranilic acid; QA,

quinolinic acid; NaMN, nicotinic acid mononucleotide; NaAD, nicotinic acid adenine dinucleotide; NAM, nicotinamide; NMN, nicotinamide mononucleotide; TDO, tryptophan 2,3-dioxygenase; KMO, kynurenine 3-monooxygenase; KYNU, kynureninase; HAAO, 3-hydroxyanthranilic acid dioxygenase; QPRT, quinolinate phosphoribosyl transferase ; NMNAT, nicotinamide mononucleotide adenylyltransferase; NADS, NAD Synthetase 1.

(**D** and **E**) The mRNA level of key enzymes in NAD^+ synthesis pathway in the liver of patients with nonalcoholic steatohepatitis (NASH) (GSE49541, D) or alcoholrelated liver disease (ALD)-induced liver fibrosis (GSE28619, E).

(**F**) Mice were subjected to bile duct ligation (BDL) or sham operation. After a peirod of two weeks, liver NAD^+, plasma NAM and intestinal NAD^+ were measured by HPLC-MSMS; mean±SD of 5 biological replicates, from 2 independent experiments.

(**G-I**) The mRNA level of key enzymes in NAD^+ synthesis pathway among in the liver of mice with metabolic associated fatty liver disease (MAFLD, GSE200482, G) or bile duct ligation (BDL, GSE40041, H and I) as determined by microarray data.

The two-sided unpaired t-test or two-sided Mann-Whitney U-test according to normal distribution in **B**, **D-I**. The statistical test method and exact *P* value are detailed in the **Table S4, Supporting Information**. *p<0.05, **p<0.01, ***p<0.001.

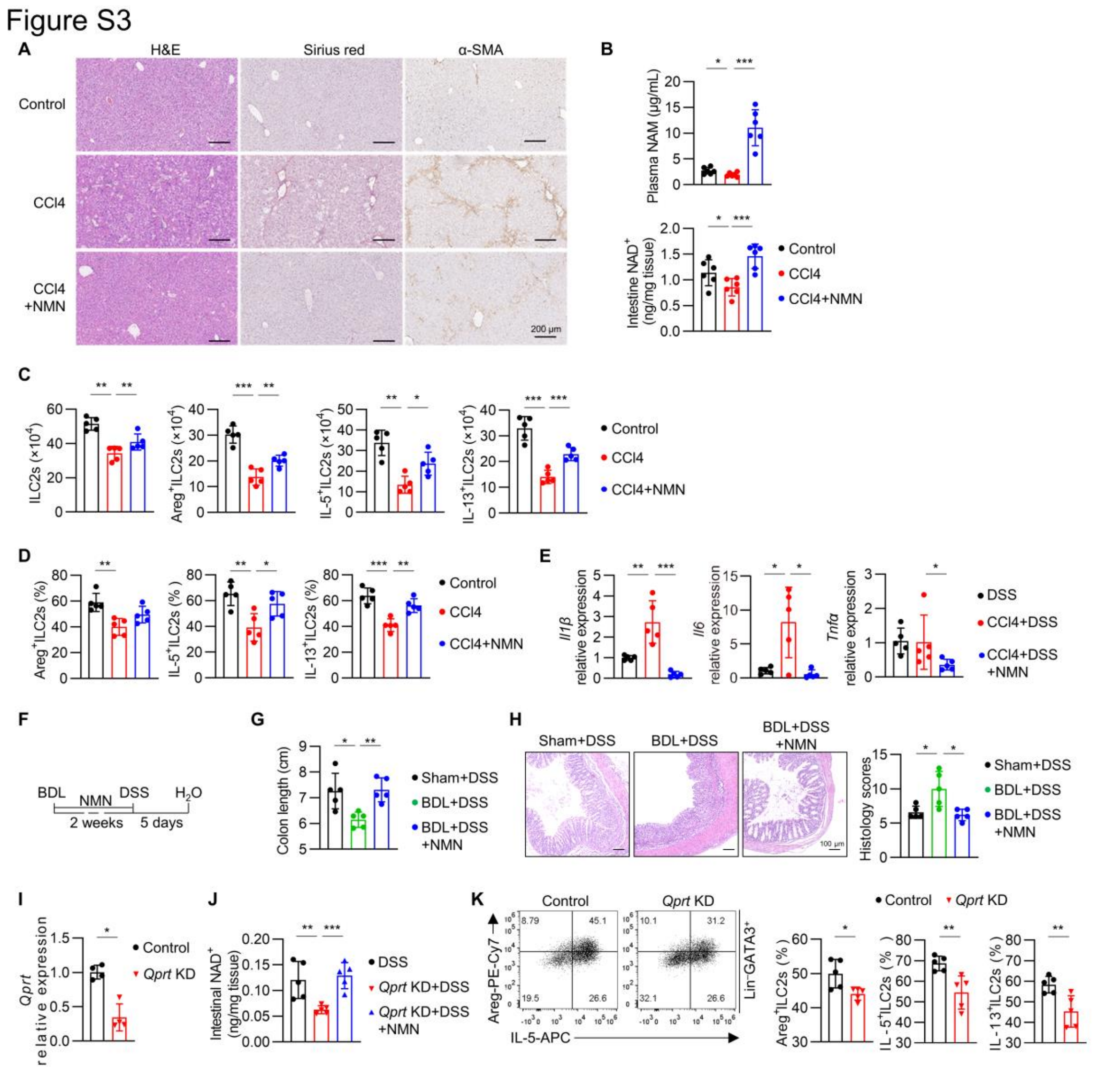

Figure S3. NMN rescues liver damage and CCl4- or BDL-aggravated DSS colitis

(**A, B**) Mice were intraperitoneally injected with carbon tetrachloride (CCl4, 2 mL/kg, twice a week) and orally administrated nicotinamide mononucleotide (NMN) for 6 weeks. The degree of liver fibrosis was evaluated by hematoxylin-eosin (H&E) and Sirius Red staining, and alfa-smooth muscle actin (α-SMA) immunohistochemistry staining. Plasma NAM and intestinal NAD^+ contents were measured by HPLC-MSMS (**B**); mean±SD of 6 biological replicates, from 3 independent experiments.

(**C, D**) Mice were intraperitoneally injected with CCl4 (2 mL/kg, twice a week) and orally administrated NMN for 6 weeks. Absolute numbers of ILC2s, $Areg^+$ ILC2s, IL-5^+ ILC2s, and

IL-13$^+$ ILC2s in large intestine (C). The frequency of Areg$^+$, IL-5$^+$, and IL-13$^+$ ILC2s in LI (D); mean±SD of 5 biological replicates, from 2 independent experiments.

(**E**) Mice were intraperitoneally injected with CCl4 (2 mL/kg, twice a week) and orally administrated NMN for 6 weeks, followed by DSS administration. The mRNA expression of pro-inflammatory cytokines *Il1β*, *Il6*, and *Tnfα* in the colons; mean±SD of 5 biological replicates, from 2 independent experiments.

(**F**-**H**) Mice were subjected to bile duct ligation (BDL) or sham operation. After a peirod of two weeks, mice were administrated DSS. Experimental strategy (F), colon lengths (G), representative H&E staining and histological severity scores of colons (H); mean±SD of 5 biological replicates, from 2 independent experiments.

(**I**) Mice were injected with liver-specific AAV carrying siRNA targeting quinolinate phosphoribosyl transferase (*Qprt*) or control siRNA. After a peirod of 4 weeks, the relative expression of *Qprt* in liver were measured; mean±SD of 4 biological replicates, from 2 independent experiments.

(**J**) Mice were injected with liver-specific AAV carrying siRNA targeting *Qprt* or control siRNA, and orally administrated NMN. After a peirod of 4 weeks, they were treated with DSS. Intestinal NAD$^+$ contents were measured by HPLC-MSMS; mean±SD of 5 biological replicates, from 2 independent experiments.

(**K**) Mice were injected with liver-specific AAV carrying siRNA targeting *Qprt* or control siRNA. After a peirod of 4 weeks, flow cytometry analysis and frequency of Areg$^+$ ILC2s, IL-5$^+$ ILC2s, and IL-13$^+$ ILC2s in the large intestine were shown; mean±SD of 5 biological replicates, from 2 independent experiments.

The two-sided unpaired t-test or two-sided Mann-Whitney U-test according to normal distribution in **B**-**E**, **G**-**K**. The statistical test method and exact *P* value are detailed in the **Table S4, Supporting Information**. *p<0.05, **p<0.01, ***p<0.001.

Figure S4

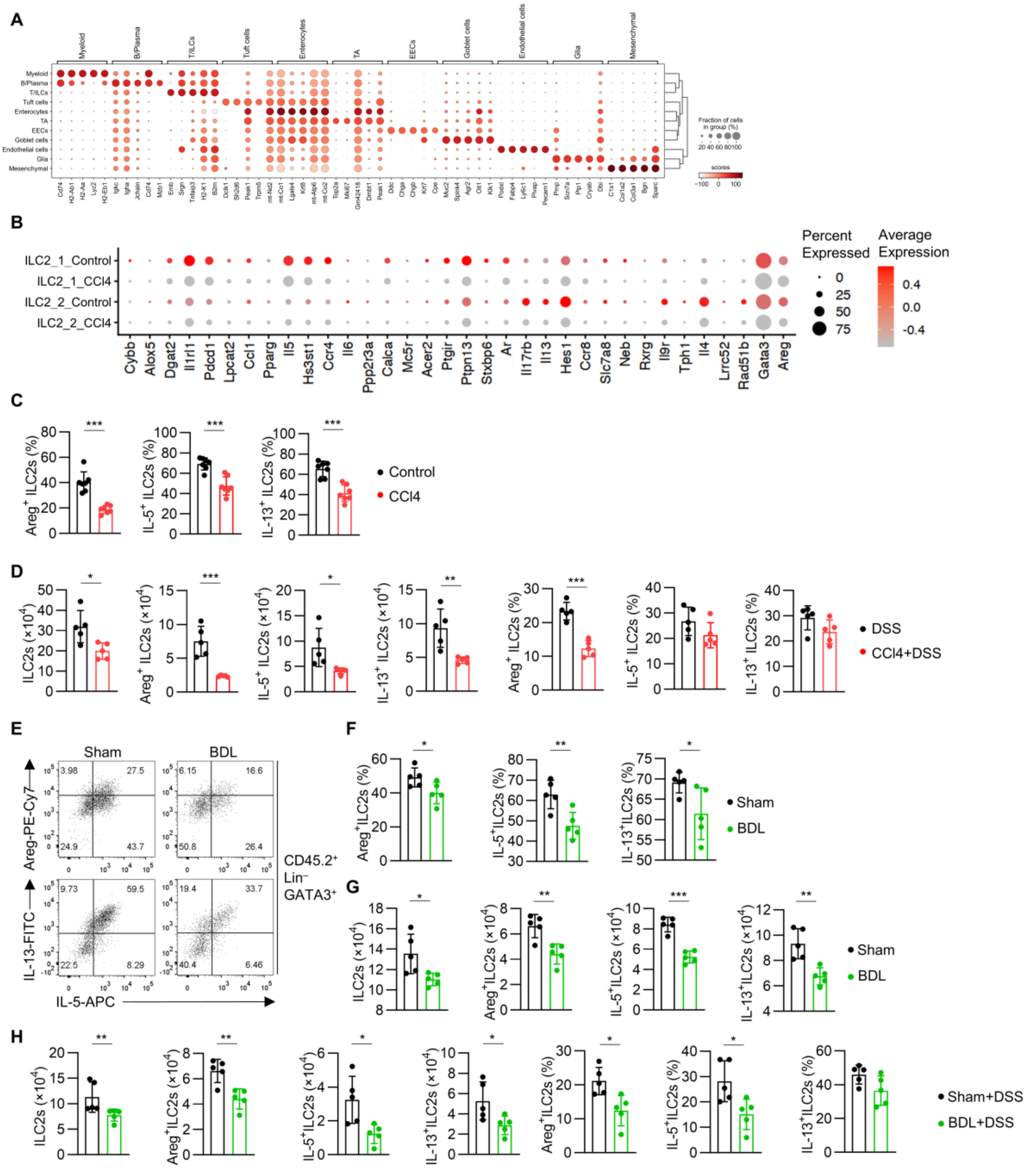

Figure S4. ILC2s contribute to liver injury-aggravated DSS colitis

(**A, B**) scRNA-seq was performed with colonic tissue from the mice treated with carbon tetrachloride (CCl4, 2 mL/kg, twice a week) for 6 weeks. Dot plot for expression of differentially genes in each cell types in the scRNA-seq dataset (A). Dot plot showing the expression of ILC2s characteristic genes (B).

(**C**) Mice were intraperitoneally injected with CCl4 (2 mL/kg, twice a week) for 6 weeks. Frequency of Areg$^+$ ILC2s, IL-5$^+$ ILC2s, and IL-13$^+$ ILC2s in large intestine (LI); mean±SD of 5 biological replicates, from 2 independent experiments.

(**D**) Mice were intraperitoneally injected with CCl4 (2 mL/kg, twice a week) for 6 weeks, followed by DSS feeding. Absolute numbers of ILC2s, Areg$^+$ ILC2s, IL-5$^+$ ILC2s, and IL-13$^+$ ILC2s, and frequency of Areg$^+$, IL-5$^+$, and IL-13$^+$ ILC2s in LI; mean±SD of 5 biological replicates, from 2 independent experiments.

(**E**-**G**) Mice were subjected to bile duct ligation (BDL) or sham operation. After a peirod of two weeks, flow cytometry analysis (E) and frequency (F) of Areg$^+$, IL-5$^+$, and IL-13$^+$ ILC2s, and absolute numbers (G) of ILC2s, Areg$^+$ ILC2s, IL-5$^+$ ILC2s, and IL-13$^+$ ILC2s in LI were measured; mean±SD of 5 biological replicates, from 2 independent experiments.

(**H**) Mice were subjected to bile duct ligation (BDL) or sham operation. After a peirod of two weeks, mice were fed with DSS in drinking water. Absolute numbers of ILC2s, Areg$^+$ ILC2s, IL-5$^+$ ILC2s, and IL-13$^+$ ILC2s, and frequency of Areg$^+$, IL-5$^+$, and IL-13$^+$ ILC2s in LI; mean±SD of 5 biological replicates, from 2 independent experiments.

The two-sided unpaired t-test or two-sided Mann-Whitney U-test according to normal distribution in **C**, **D**, **F**-**H**. The statistical test method and exact *P* value are detailed in the **Table S4, Supporting Information**. *$p<0.05$, **$p<0.01$, ***$p<0.001$.

Figure S5

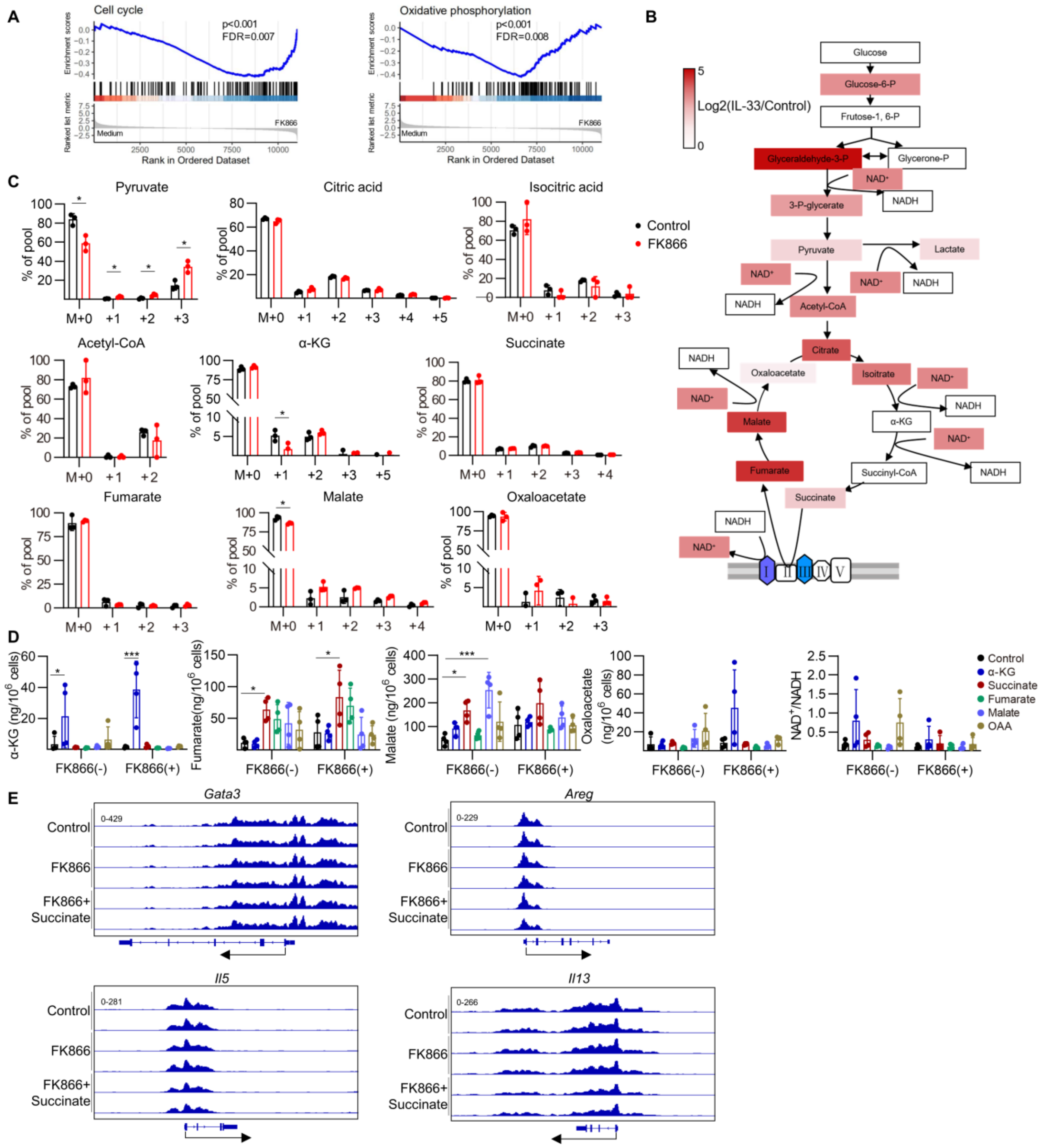

Figure S5. NAMPT inhibition impairs ILC2s function

(**A**) Sorted large intestine (LI) ILC2s treated with FK866 for 16 h and then subjected to RNA-seq. Gene set enrichment analysis of the RNA sequencing data.

(**B**) The relative abundance of indicated labeled metabolites in IL-33-treated vs control ILC2s is represented by a color scale (values are expressed as log ratio of means) (GSE166081).

(**C)** Sorted LI ILC2s were cultured in the presence of IL-2, IL-7, IL-25, and IL-33 for 5 days, followed by $^{13}C_6$-glucose and FK866 treatment for 24 hours. The fraction proportion of ^{13}C-derived TCA cycle intermediates in ILC2. Data were presented as mean±SD, n=3 per group.

(**D**) Sorted LI ILC2s were cultured in the presence of IL-2, IL-7, IL-25, and IL-33 for 5 days, followed by the indicated treatment for 24 hours. Levels of α-ketoglutarate (α-KG), fumarate, malate, oxaloacetate (OAA) and NAD^+/NADH in ILC2s treated with α-KG (5 mM), succinate (10 mM), fumarate (5 mM), malate (10 mM), and OAA (10 mM), with or without FK866 (200 nM); mean±SD of 4 biological replicates, from 2 independent experiments.

(**E**) Sorted LI ILC2s were cultured in the presence of IL-2, IL-7, IL-25, and IL-33 for 5 days, followed by the indicated treatment for 24 hours. Representative CUT&Tag signals of H3K4me3 at the loci of indicted genes in ILC2s.

The two-sided unpaired t-test or two-sided Mann-Whitney U-test according to normal distribution in **C**. The One-way ANOVA with Dunnett or Kruskal Wallis with Dunns according to normal distribution in **D**. The statistical test method and exact *P* value are detailed in the **Table S4, Supporting Information**. $^*p<0.05$, $^{**}p<0.01$, $^{***}p<0.001$.

Figure S6

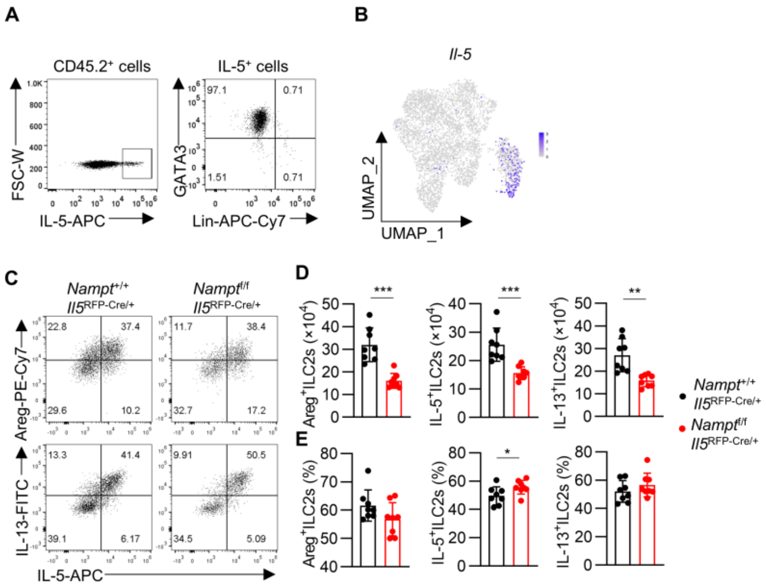

Figure S6. NAD$^+$ maintains intestinal ILC2s

(**A**) Flow cytometry analysis of expression of IL-5 in large intestine (LI) tissue of mice.

(**B**) scRNA-seq was performed with colonic tissue from the mice treated with carbon tetrachloride (CCl4, 2 mL/kg, twice a week) for 6 weeks. Uniform manifold approximation and projection (UMAP) plots showing the expression of *Il5* in T/ILCs.

(**C**-**E**) Flow cytometry analysis (C), frequency of Areg$^+$, IL-5$^+$, and IL-13$^+$ ILC2s after gating on CD45.2$^+$Lin$^-$GATA3$^+$ ILC2s (D) and absolute numbers of ILC2s, Areg$^+$ ILC2s, IL-5$^+$ ILC2s, and IL-13$^+$ ILC2s (E) in the LI of *Nampt*$^{+/+}$*Il5*$^{\text{RFP-Cre/+}}$ or *Nampt*$^{f/f}$*Il5*$^{\text{RFP-Cre/+}}$ littermate mice; mean±SD of 8 biological replicates, from 3 independent experiments.

The two-sided unpaired t-test or two-sided Mann-Whitney U-test according to normal distribution in **D, E**. The statistical test method and exact *P* value are detailed in the Supplemental table 4. *p<0.05, **p<0.01, ***p<0.001.

Figure S7

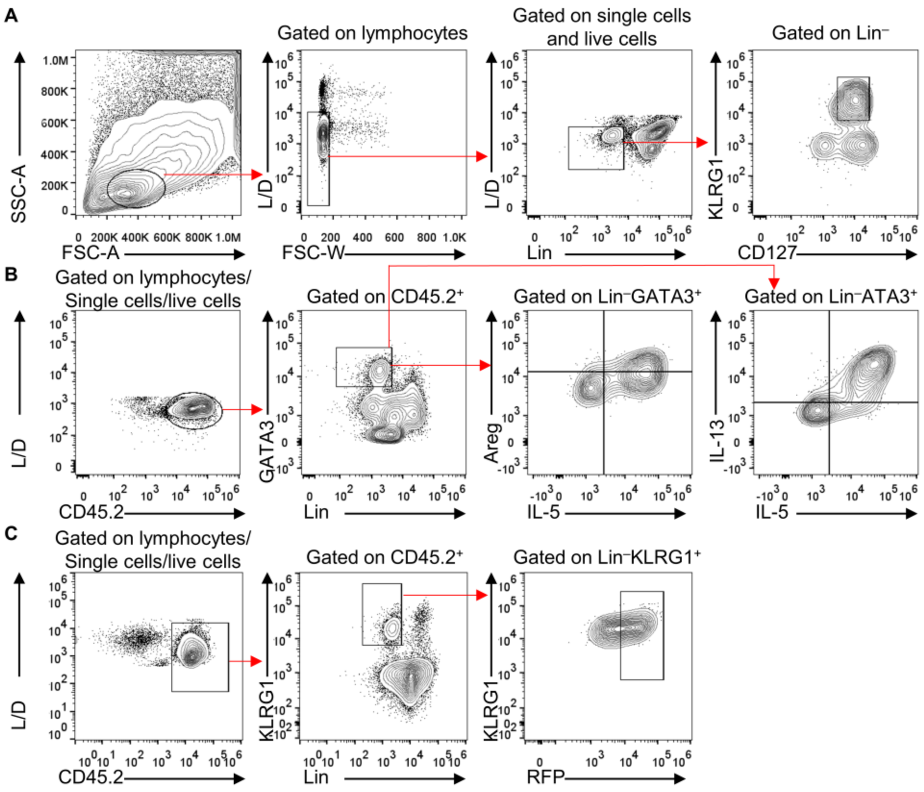

Figure S7. Gating strategy of colonic ILC2s by flow cytometry.

(**A**-**C**) For flow cytometry analyses, live cells were gated after singlet discrimination. Mouse ILC2 cells were gated based on the following markers: $Lin^{-}CD127^{+}KLRG1^{+}$, $CD45.2^{+}Lin^{-}Gata3^{+}$, or $CD45.2^{+}Lin^{-}KLRG1^{+}$.

Table S1. Baseline characteristics of included patients in retrospective chart review

Patients	UC	UC	UC
Liver injury	Non (ALT and/or AST≤1×ULN)	Grade I (1×ULN < ALT and/or AST < 2×ULN)	Grade II (ALT and/or AST≥ 2×ULN)
Patients' numbers	35	35	15
Age	48.8±13.8	49.0±14.5	44.5±17.3
Serum ALT (U/L)	17.6±9.0	46.1±13.1	115.5±48.1
Serum AST (U/L)	23.0±8.9	39.3±9.2	65.1±27.1

Liver injury was defined as the levels of alanine aminotransferase (ALT) and/or aspartate aminotransferase (AST) exceeding the upper limit of normal (ULN). ULN of ALT and AST

were defined according to the criteria of The Asian Pacific Association for the Study of the Liver (40 U/L for both genders).

Table S2. Demographic information of UC participants in retrospective chart review

ID	Age (years)	Gender	Weight (kg)	BMI (kg/m^2)	ALT level	AST level
U-1	24	Male	51	17.6	ALT≤1×ULN	AST≤1×ULN
U-2	31	Male	68	21.0	ALT≤1×ULN	AST≤1×ULN
U-3	33	Male	64	19.8	ALT≤1×ULN	AST≤1×ULN
U-4	33	Male	52	19.6	ALT≤1×ULN	AST≤1×ULN
U-5	42	Male	65	23.3	ALT≤1×ULN	AST≤1×ULN
U-6	47	Male	55	20.2	ALT≤1×ULN	AST≤1×ULN
U-7	48	Male	73	25.9	ALT≤1×ULN	AST≤1×ULN
U-8	49	Male	70	23.7	ALT≤1×ULN	AST≤1×ULN
U-9	51	Male	76	27.9	ALT≤1×ULN	AST≤1×ULN
U-10	53	Male	65	23.0	ALT≤1×ULN	AST≤1×ULN
U-11	54	Male	90	27.8	ALT≤1×ULN	AST≤1×ULN
U-12	54	Male	86	26.5	ALT≤1×ULN	AST≤1×ULN
U-13	56	Male	66	23.4	ALT≤1×ULN	AST≤1×ULN
U-14	59	Male	77	25.4	ALT≤1×ULN	AST≤1×ULN
U-15	60	Male	70	24.2	ALT≤1×ULN	AST≤1×ULN
U-16	66	Male	63	19.9	ALT≤1×ULN	AST≤1×ULN
U-17	67	Male	67	25.2	ALT≤1×ULN	AST≤1×ULN
U-18	68	Male	85	30.8	ALT≤1×ULN	AST≤1×ULN
U-19	70	Male	67	24.6	ALT≤1×ULN	AST≤1×ULN
U-20	22	Female	63	23.1	ALT≤1×ULN	AST≤1×ULN
U-21	32	Female	58	18.9	ALT≤1×ULN	AST≤1×ULN
U-22	33	Female	50	20.0	ALT≤1×ULN	AST≤1×ULN
U-23	40	Female	53	19.5	ALT≤1×ULN	AST≤1×ULN
U-24	45	Female	64	25.6	ALT≤1×ULN	AST≤1×ULN
U-25	45	Female	57	21.5	ALT≤1×ULN	AST≤1×ULN
U-26	50	Female	48	19.2	ALT≤1×ULN	AST≤1×ULN
U-27	50	Female	66	23.1	ALT≤1×ULN	AST≤1×ULN
U-28	56	Female	75	26.6	ALT≤1×ULN	AST≤1×ULN
U-29	57	Female	48	18.8	ALT≤1×ULN	AST≤1×ULN
U-30	58	Female	53	22.1	ALT≤1×ULN	AST≤1×ULN
U-31	62	Female	49	18.0	ALT≤1×ULN	AST≤1×ULN
U-32	67	Female	52	23.7	ALT≤1×ULN	AST≤1×ULN
U-33	67	Male	55	20.2	ALT≤1×ULN	AST≤1×ULN
U-34	35	Male	63	19.4	ALT≤1×ULN	AST≤1×ULN
U-35	49	Male	75	24.8	ALT≤1×ULN	AST≤1×ULN
U-LI-1	40	Female	54	21.1	1×ULN < ALT< 2×ULN	AST≤1×ULN
U-LI-2	59	Female	50	18.8	1×ULN < ALT< 2×ULN	1×ULN < AST< 2×ULN
U-LI-3	45	Female	55	21.2	ALT≤1×ULN	1×ULN < AST< 2×ULN
U-LI-4	43	Female	50	19.5	ALT≤1×ULN	1×ULN < AST< 2×ULN
U-LI-5	35	Female	57	22.5	ALT≤1×ULN	1×ULN < AST< 2×ULN
U-LI-6	55	Female	55	22.9	ALT≤1×ULN	1×ULN < AST< 2×ULN
U-LI-7	61	Female	60	24.0	ALT≤1×ULN	1×ULN < AST< 2×ULN
U-LI-8	51	Female	50	20.0	1×ULN < ALT< 2×ULN	1×ULN < AST< 2×ULN
U-LI-9	41	Male	58	22.7	1×ULN < ALT< 2×ULN	1×ULN < AST< 2×ULN
U-LI-10	24	Male	55	17.4	1×ULN < ALT< 2×ULN	1×ULN < AST< 2×ULN
U-LI-11	54	Male	64	22.1	1×ULN < ALT< 2×ULN	AST≤1×ULN

U-LI-12	62	Male	50	16.9	1×ULN < ALT< 2×ULN	AST≤1×ULN
U-LI-13	60	Male	59	21.7	1×ULN < ALT< 2×ULN	AST≤1×ULN
U-LI-14	32	Male	64	21.4	1×ULN < ALT< 2×ULN	AST≤1×ULN
U-LI-15	56	Male	79	28.3	1×ULN < ALT< 2×ULN	AST≤1×ULN
U-LI-16	62	Male	50	17.9	1×ULN < ALT< 2×ULN	AST≤1×ULN
U-LI-17	23	Male	55	18.4	1×ULN < ALT< 2×ULN	1×ULN < AST< 2×ULN
U-LI-18	42	Male	65	22.8	1×ULN < ALT< 2×ULN	1×ULN < AST< 2×ULN
U-LI-19	42	Female	45	18.0	ALT≤1×ULN	1×ULN < AST< 2×ULN
U-LI-20	55	Male	80	27.0	1×ULN < ALT< 2×ULN	AST≤1×ULN
U-LI-21	72	Male	70	24.2	1×ULN < ALT< 2×ULN	1×ULN < AST< 2×ULN
U-LI-22	65	Male	60	19.6	1×ULN < ALT< 2×ULN	AST≤1×ULN
U-LI-23	37	Female	55	21.5	ALT≤1×ULN	1×ULN < AST< 2×ULN
U-LI-24	54	Male	56	20.6	ALT≤1×ULN	1×ULN < AST< 2×ULN
U-LI-25	72	Female	55	21.5	1×ULN < ALT< 2×ULN	1×ULN < AST< 2×ULN
U-LI-26	58	Male	70	24.2	1×ULN < ALT< 2×ULN	1×ULN < AST< 2×ULN
U-LI-27	65	Male	60	20.8	1×ULN < ALT< 2×ULN	AST≤1×ULN
U-LI-28	45	Male	58	18.3	1×ULN < ALT< 2×ULN	1×ULN < AST< 2×ULN
U-LI-29	19	Female	60	20.0	1×ULN < ALT< 2×ULN	AST≤1×ULN
U-LI-30	31	Male	72	25.5	1×ULN < ALT< 2×ULN	AST≤1×ULN
U-LI-31	23	Male	85	25.7	1×ULN < ALT< 2×ULN	AST≤1×ULN
U-LI-32	52	Male	68	23.5	1×ULN < ALT< 2×ULN	AST≤1×ULN
U-LI-33	58	Female	40	17.8	ALT≤1×ULN	1×ULN < AST< 2×ULN
U-LI-34	54	Male	78	27.0	ALT≤1×ULN	1×ULN < AST< 2×ULN
U-LI-35	69	Male	60	20.0	ALT≤1×ULN	1×ULN < AST< 2×ULN
U-LI-36	67	Female	40	19.0	ALT≤1×ULN	AST ≥ 2×ULN
U-LI-37	49	Female	70	27.3	ALT ≥ 2×ULN	1×ULN < AST< 2×ULN
U-LI-38	47	Male	58	18.9	ALT ≥ 2×ULN	1×ULN < AST< 2×ULN
U-LI-39	58	Male	60	22.0	ALT ≥ 2×ULN	AST ≥ 2×ULN
U-LI-40	20	Male	60	19.4	ALT ≥ 2×ULN	AST≤1×ULN
U-LI-41	53	Male	75	30.0	ALT ≥ 2×ULN	AST≤1×ULN
U-LI-42	54	Male	85	26.2	ALT ≥ 2×ULN	AST ≥ 2×ULN
U-LI-43	49	Male	61	18.8	1×ULN < ALT< 2×ULN	AST ≥ 2×ULN
U-LI-44	62	Female	65	24.5	ALT ≥ 2×ULN	AST ≥ 2×ULN
U-LI-45	22	Male	60	19.6	ALT ≥ 2×ULN	AST≤1×ULN
U-LI-46	54	Male	45	14.7	ALT ≥ 2×ULN	1×ULN < AST< 2×ULN
U-LI-47	23	Male	75	21.4	ALT ≥ 2×ULN	AST≤1×ULN
U-LI-48	63	Male	59	19.5	ALT ≥ 2×ULN	1×ULN < AST< 2×ULN
U-LI-49	18	Female	59	19.9	ALT ≥ 2×ULN	AST ≥ 2×ULN
U-LI-50	28	Male	65	21.2	ALT ≥ 2×ULN	1×ULN < AST< 2×ULN

Table S3 Demographic information of healthy donors and liver cirrhosis participants

ID	Age (years)	Gender	Weight (kg)	BMI (kg/m^2)	Patients	Index
HC-1	32	Female	70.0	25.7	Healthy donor	Plasma, non-targeted metabolomics; NAM measurement
HC-2	62	Male	70.0	22.1	Healthy donor	Plasma, non-targeted metabolomics; NAM measurement
HC-3	62	Male	85.0	28.7	Healthy donor	Plasma, non-targeted metabolomics; NAM measurement

HC-4	59	Male	75.0	27.5	Healthy donor	Plasma, non-targeted metabolomics; NAM measurement
HC-5	57	Female	53.0	22.6	Healthy donor	Plasma, non-targeted metabolomics; NAM measurement
HC-6	58	Female	75.0	26.6	Healthy donor	Plasma, non-targeted metabolomics; NAM measurement
HC-7	48	Female	65.0	28.9	Healthy donor	Plasma, non-targeted metabolomics; NAM measurement
HC-8	63	Male	75.0	24.8	Healthy donor	Plasma, non-targeted metabolomics; NAM measurement
HC-9	62	Female	66.0	25.8	Healthy donor	Plasma, non-targeted metabolomics; NAM measurement
HC-10	54	Female	61.0	23.8	Healthy donor	NAM measurement
HC-11	54	Male	83.0	28.7	Healthy donor	NAM measurement
HC-12	53	Female	56.5	21.3	Healthy donor	NAM measurement
HC-13	44	Male	53.0	18.8	Healthy donor	NAM measurement
HC-14	28	Male	50.0	18.4	Healthy donor	NAM measurement
HC-15	53	Female	62.5	25.0	Healthy donor	NAM measurement
HC-16	62	Male	68.0	23.5	Healthy donor	NAM measurement
HC-17	51	Male	72.5	23.7	Healthy donor	NAM measurement
HC-18	51	Female	68.0	24.4	Healthy donor	NAM measurement
HC-19	74	Male	67.0	23.2	Healthy donor	NAM measurement
HC-20	61	Male	81.0	25.6	Healthy donor	NAM measurement
HC-21	55	Male	67.0	22.4	Healthy donor	NAM measurement
HC-22	32	Female	61.0	22.4	Healthy donor	NAM measurement
HC-23	73	Female	55.0	22.9	Healthy donor	NAM measurement
HC-24	15	Male	55.0	20.2	Healthy donor	NAM measurement
HC-25	65	Male	60.0	21.3	Healthy donor	NAM measurement
HC-26	57	Male	65.0	23.3	Healthy donor	NAM measurement
HC-27	55	Male	65.0	22.8	Healthy donor	NAM measurement
HC-28	72	Male	85.0	26.8	Healthy donor	NAM measurement
HC-29	48	Male	114.0	35.2	Healthy donor	NAM measurement
HC-30	34	Male	97.0	29.9	Healthy donor	NAM measurement
HC-31	64	Female	77.0	29.3	Healthy donor	PBMC, Flow Cytometry
HC-32	70	Male	68.0	21.7	Healthy donor	PBMC, Flow Cytometry
HC-33	47	Male	70.0	24.2	Healthy donor	PBMC, Flow Cytometry
HC-34	58	Female	70.0	28.0	Healthy donor	PBMC, Flow Cytometry
HC-35	51	Male	68.0	22.5	Healthy donor	PBMC, Flow Cytometry
HC-36	68	Male	73.0	26.2	Healthy donor	PBMC, Flow Cytometry
HC-37	45	Male	69.0	22.3	Healthy donor	PBMC, Flow Cytometry
HC-38	68	Male	70.0	24.5	Healthy donor	PBMC, Flow Cytometry
HC-39	76	Male	80.0	30.9	Healthy donor	PBMC, Flow Cytometry
HC-40	32	Male	75.0	23.4	Healthy donor	PBMC, Flow Cytometry
HC-41	36	Male	75.0	24.5	Healthy donor	LPMNC, Flow Cytometry
HC-42	35	Female	65.0	23.9	Healthy donor	LPMNC, Flow Cytometry
HC-43	52	Female	55.0	22.9	Healthy donor	LPMNC, Flow Cytometry

HC-44	61	Male	67.0	21.9	Healthy donor	LPMNC, Flow Cytometry
HC-45	76	Male	70.0	24.2	Healthy donor	LPMNC, Flow Cytometry
LC-1	60	Female	59.0	23.6	Cirrhosis	Plasma, non-targeted metabolomics; NAM measurement
LC-2	62	Male	67.0	21.9	Cirrhosis	Plasma, non-targeted metabolomics; NAM measurement
LC-3	44	Male	65.0	22.5	Cirrhosis	Plasma, non-targeted metabolomics; NAM measurement
LC-4	55	Male	75.0	26.0	Cirrhosis	Plasma, non-targeted metabolomics; NAM measurement
LC-5	50	Male	84.0	27.4	Cirrhosis	Plasma, non-targeted metabolomics; NAM measurement
LC-6	38	Male	61.0	18.8	Cirrhosis	Plasma, non-targeted metabolomics; NAM measurement
LC-7	58	Male	65.0	21.2	Cirrhosis	Plasma, non-targeted metabolomics; NAM measurement
LC-8	43	Male	61.0	22.7	Cirrhosis	Plasma, non-targeted metabolomics; NAM measurement
LC-9	57	Male	68.0	24.1	Cirrhosis	Plasma, non-targeted metabolomics; NAM measurement
LC-10	61	Female	67.0	24.6	Cirrhosis	NAM measurement
LC-11	57	Male	63.5	22.0	Cirrhosis	NAM measurement
LC-12	54	Male	95.0	31.0	Cirrhosis	NAM measurement
LC-13	59	Female	61.5	24.0	Cirrhosis	NAM measurement
LC-14	59	Male	70.0	24.8	Cirrhosis	NAM measurement
LC-15	50	Male	61.0	22.7	Cirrhosis	NAM measurement
LC-16	39	Male	89.0	28.4	Cirrhosis	NAM measurement
LC-17	63	Female	67.0	24.6	Cirrhosis	NAM measurement
LC-18	60	Female	75.0	26.9	Cirrhosis	NAM measurement
LC-19	58	Female	50.0	20.8	Cirrhosis	NAM measurement
LC-20	32	Male	70.5	22.5	Cirrhosis	NAM measurement
LC-21	58	Male	56.0	20.1	Cirrhosis	NAM measurement
LC-22	59	Female	61.0	23.8	Cirrhosis	NAM measurement
LC-23	43	Male	94.0	30.3	Cirrhosis	NAM measurement
LC-24	55	Male	79.0	24.9	Cirrhosis	NAM measurement
LC-25	69	Male	86.0	27.8	Cirrhosis	NAM measurement
LC-26	69	Male	67.0	24.0	Cirrhosis	NAM measurement
LC-27	65	Female	60.0	24.0	Cirrhosis	NAM measurement
LC-28	40	Female	60.0	22.6	Cirrhosis	NAM measurement
LC-29	61	Female	55.0	20.0	Cirrhosis	NAM measurement
LC-30	71	Female	45.0	19.0	Cirrhosis	NAM measurement
LC-31	55	Male	66.0	22.3	Cirrhosis	PBMC, Flow Cytometry
LC-32	64	Male	67.0	23.2	Cirrhosis	PBMC, Flow Cytometry
LC-33	56	Male	62.0	21.5	Cirrhosis	PBMC, Flow Cytometry

LC-34	50	Male	82.0	28.4	Cirrhosis	PBMC, Flow Cytometry
LC-35	67	Male	60.0	19.8	Cirrhosis	PBMC, Flow Cytometry
LC-36	53	Male	105.0	33.1	Cirrhosis	PBMC, Flow Cytometry
LC-37	43	Female	50.0	20.8	Cirrhosis	PBMC, Flow Cytometry
LC-38	43	Male	60.0	20.8	Cirrhosis	PBMC, Flow Cytometry
LC-39	39	Male	68.0	22.5	Cirrhosis	PBMC, Flow Cytometry
LC-40	50	Female	56.0	19.4	Cirrhosis	PBMC, Flow Cytometry

Table S4 *P* values and statistics

	Description	Test	Comparison	p-value	significance code
Figure 1A	UCEIS	Mann Whitney	ALT and/or AST≤1×ULN vs 1×ULN<ALT and/or AST<2×ULN	0.0004	***
Figure 1A	UCEIS	Mann Whitney	1×ULN<ALTand/or AST<2×ULN vs ALT and/or AST≥2×ULN	0.0003	***
Figure 1A	UCEIS	Mann Whitney	ALT and/or AST≤1×ULN vs ALT and/or AST≥2×ULN	<0.0001	***
Figure 1B	AST	Unpaired t test	Control vs CCl4	0.0006	***
Figure 1B	ALT	Unpaired t test	Control vs CCl4	<0.0001	***
Figure 1D	% intitial weight D0	Unpaired t test	DSS vs DSS+CCl4	<0.0001	***
Figure 1D	% intitial weight D1	Unpaired t test	DSS vs DSS+CCl4	0.0281	*
Figure 1D	% intitial weight D2	Mann Whitney	DSS vs DSS+CCl4	0.0530	ns
Figure 1D	% intitial weight D3	Unpaired t test	DSS vs DSS+CCl4	0.0389	*
Figure 1D	% intitial weight D4	Unpaired t test	DSS vs DSS+CCl4	0.0471	*
Figure 1D	% intitial weight D5	Unpaired t test	DSS vs DSS+CCl4	0.0474	*
Figure 1E	Colon length	Mann Whitney	DSS vs DSS+CCl4	0.0012	**
Figure 1F	Histology scores	Unpaired t test	DSS vs DSS+CCl4	0.0074	**
Figure 1G	% intitial weight D1	Unpaired t test	Sham+DSS vs BDL+DSS	0.2199	ns
Figure 1G	% intitial weight D2	Unpaired t test	Sham+DSS vs BDL+DSS	0.2935	ns
Figure 1G	% intitial weight D3	Unpaired t test	Sham+DSS vs BDL+DSS	0.0507	ns
Figure 1G	% intitial weight D4	Unpaired t test	Sham+DSS vs BDL+DSS	0.0043	**
Figure 1G	% intitial weight D5	Unpaired t test	Sham+DSS vs BDL+DSS	0.0013	**
Figure 1H	Colon length	Unpaired t test	Sham+DSS vs BDL+DSS	<0.0001	***
Figure 1I	Histology scores	Mann Whitney	Sham+DSS vs BDL+DSS	0.0079	**
Figure 2C	Plasma NAM	Mann Whitney	Healthy donor vs Liver cirrhosis	<0.0001	***

Figure 2D	Relative expression *Tdo2*	Unpaired t test	Control vs CCl4	<0.0001	***
Figure 2D	Relative expression *Ido2*	Unpaired t test	Control vs CCl4	<0.0001	***
Figure 2D	Relative expression *Kmo*	Unpaired t test	Control vs CCl4	<0.0001	***
Figure 2D	Relative expression *Kynu*	Mann Whitney	Control vs CCl4	0.4095	ns
Figure 2D	Relative expression *Qprt*	Mann Whitney	Control vs CCl4	0.3474	ns
Figure 2D	Relative expression *Nmnat1*	Unpaired t test	Control vs CCl4	0.8509	ns
Figure 2D	Relative expression *Nmnat2*	Mann Whitney	Control vs CCl4	0.5137	ns
Figure 2D	Relative expression *Nmnat3*	Unpaired t test	Control vs CCl4	0.1793	ns
Figure 2D	Relative expression *Nampt*	Unpaired t test	Control vs CCl4	0.6493	ns
Figure 2E	Liver NAD^+	Unpaired t test	Control vs CCl4	0.0004	***
Figure 2E	Plasma NAM	Unpaired t test	Control vs CCl4	0.0014	**
Figure 2E	Intestine NAD^+	Unpaired t test	Control vs CCl4	0.0065	**
Figure 2F	% intitial weight D0	Unpaired t test	DSS vs CCl4+DSS	0.0004	***
Figure 2F	% intitial weight D0	Unpaired t test	CCl4+DSS vs CCl4+DSS+NMN	0.5467	ns
Figure 2F	% intitial weight D1	Unpaired t test	DSS vs CCl4+DSS	0.2852	ns
Figure 2F	% intitial weight D1	Unpaired t test	CCl4+DSS vs CCl4+DSS+NMN	0.2463	ns
Figure 2F	% intitial weight D2	Unpaired t test	DSS vs CCl4+DSS	0.0054	**
Figure 2F	% intitial weight D2	Unpaired t test	CCl4+DSS vs CCl4+DSS+NMN	0.9637	ns
Figure 2F	% intitial weight D3	Unpaired t test	DSS vs CCl4+DSS	0.0084	**
Figure 2F	% intitial weight D3	Unpaired t test	CCl4+DSS vs CCl4+DSS+NMN	0.5877	ns
Figure 2F	% intitial weight D4	Unpaired t test	DSS vs CCl4+DSS	0.0017	**
Figure 2F	% intitial weight D4	Unpaired t test	CCl4+DSS vs CCl4+DSS+NMN	0.0479	*
Figure 2F	% intitial weight D5	Unpaired t test	DSS vs CCl4+DSS	0.0051	**
Figure 2F	% intitial weight D5	Unpaired t test	CCl4+DSS vs CCl4+DSS+NMN	0.0074	**
Figure 2G	Colon length	Unpaired t test	DSS vs CCl4+DSS	0.0272	*
Figure 2G	Colon length	Mann Whitney	CCl4+DSS vs CCl4+DSS+NMN	0.0006	***
Figure 2H	Histology scores	Mann Whitney	DSS vs CCl4+DSS	0.0006	***
Figure 2H	Histology scores	Mann Whitney	CCl4+DSS vs CCl4+DSS+NMN	0.0006	***
Figure 2I	Liver NAD+	Unpaired t test	Control vs Qprt KD	0.0207	*
Figure 2I	Plasma NAM	Unpaired t test	Control vs Qprt KD	0.0084	**

Figure 2I	Intestine NAD+	Unpaired t test	Control vs Qprt KD	0.0012	**
Figure 2J	% intitial weight D1	Unpaired t test	DSS vs Qprt KD+DSS	0.5991	ns
Figure 2J	% intitial weight D1	Unpaired t test	Qprt KD+DSS vs Qprt KD+DSS+NMN	>0.999	ns
Figure 2J	% intitial weight D2	Unpaired t test	DSS vs Qprt KD+DSS	0.0467	*
Figure 2J	% intitial weight D2	Unpaired t test	Qprt KD+DSS vs Qprt KD+DSS+NMN	0.0128	*
Figure 2J	% intitial weight D3	Unpaired t test	DSS vs Qprt KD+DSS	0.2832	ns
Figure 2J	% intitial weight D3	Unpaired t test	Qprt KD+DSS vs Qprt KD+DSS+NMN	0.1653	ns
Figure 2J	% intitial weight D4	Unpaired t test	DSS vs Qprt KD+DSS	0.1371	ns
Figure 2J	% intitial weight D4	Unpaired t test	Qprt KD+DSS vs Qprt KD+DSS+NMN	>0.999	ns
Figure 2J	% intitial weight D5	Unpaired t test	DSS vs Qprt KD+DSS	0.0024	**
Figure 2J	% intitial weight D5	Unpaired t test	Qprt KD+DSS vs Qprt KD+DSS+NMN	0.0018	**
Figure 2K	Colon length	Unpaired t test	DSS vs Qprt KD+DSS	0.0027	**
Figure 2K	Colon length	Unpaired t test	Qprt KD+DSS vs Qprt KD+DSS+NMN	0.0004	***
Figure 2L	Histology scores	Mann Whitney	DSS vs Qprt KD+DSS	0.0238	*
Figure 2L	Histology scores	Mann Whitney	Qprt KD+DSS vs Qprt KD+DSS+NMN	0.0079	**
Figure 3D	Rag2-/- Colon length	Unpaired t test	DSS vs CCl4+DSS	0.0038	**
Figure 3D	Rag2-/- Colon length	Mann Whitney	CCl4+DSS vs CCl4+DSS+NMN	0.0043	**
Figure 3F	Rag2-/- Histology scores	Unpaired t test	DSS vs CCl4+DSS	<0.0001	***
Figure 3F	Rag2-/- Histology scores	Unpaired t test	CCl4+DSS vs CCl4+DSS+NMN	<0.0001	***
Figure 3H	Rag2-/-Il2rg-/- Colon length	Unpaired t test	DSS vs CCl4+DSS	0.2193	ns
Figure 3H	Rag2-/-Il2rg-/- Colon length	Unpaired t test	CCl4+DSS vs CCl4+DSS+NMN	0.4987	ns
Figure 3J	Rag2-/-Il2rg-/- Histology scores	Unpaired t test	DSS vs CCl4+DSS	0.5108	ns
Figure 3J	Rag2-/-Il2rg-/- Histology scores	Unpaired t test	CCl4+DSS vs CCl4+DSS+NMN	0.8417	ns
Figure 3M	ILC2s ($\times 10^4$)	Mann Whitney	Control vs CCl4	0.0530	ns
Figure 3M	Areg$^+$ ILC2s ($\times 10^4$)	Unpaired t test	Control vs CCl4	<0.0001	***
Figure 3M	IL-5$^+$ ILC2s ($\times 10^4$)	Mann Whitney	Control vs CCl4	0.0006	***
Figure 3M	IL-13$^+$ ILC2s ($\times 10^4$)	Unpaired t test	Control vs CCl4	0.0006	***
Figure 3O	Areg+ ILC2s (%)	Unpaired t test	Healthy donors vs Liver cirrhosis	0.0006	***
Figure 3P	Colon length	Unpaired t test	DSS vs CCl4+DSS	0.0019	**

Figure 3P	Colon length	Unpaired t test	CCl4+DSS vs CCl4+DSS+ILC2s	0.0082	**
Figure 3R	Histology scores	Mann Whitney	DSS vs CCl4+DSS	0.0079	**
Figure 3R	Histology scores	Mann Whitney	CCl4+DSS vs CCl4+DSS+ILC2s	0.0317	*
Figure 4C	Viable cells (%)	Mann Whitney	Control vs GTN	0.0006	***
Figure 4C	Areg+ ILC2s (%)	Unpaired t test	Control vs GTN	<0.0001	***
Figure 4C	IL-5+ ILC2s (%)	Mann Whitney	Control vs GTN	0.0006	***
Figure 4C	IL-13+ ILC2s (%)	Mann Whitney	Control vs GTN	0.0006	***
Figure 4D	Viable cells (%)	Kruskal Wallis with Dunns	Control vs PA	>0.9999	ns
Figure 4D	Viable cells (%)	Kruskal Wallis with Dunns	Control vs 2-HNA	>0.9999	ns
Figure 4D	Viable cells (%)	Kruskal Wallis with Dunns	Control vs FK866	0.0379	*
Figure 4D	Viable cells (%)	Kruskal Wallis with Dunns	Control vs FK866+NMN	>0.9999	ns
Figure 4D	Viable cells (%)	Kruskal Wallis with Dunns	PA vs 2-HNA	>0.9999	ns
Figure 4D	Viable cells (%)	Kruskal Wallis with Dunns	PA vs FK866	0.8515	ns
Figure 4D	Viable cells (%)	Kruskal Wallis with Dunns	PA vs FK866+NMN	0.3934	ns
Figure 4D	Viable cells (%)	Kruskal Wallis with Dunns	2-HNA vs FK866	0.0722	ns
Figure 4D	Viable cells (%)	Kruskal Wallis with Dunns	2-HNA vs FK866+NMN	>0.9999	ns
Figure 4D	Viable cells (%)	Kruskal Wallis with Dunns	FK866 vs FK866+NMN	0.0016	**
Figure 4F	Areg+ ILC2s (%)	Kruskal Wallis with Dunns	Control vs PA	>0.9999	ns
Figure 4F	Areg+ ILC2s (%)	Kruskal Wallis with Dunns	Control vs 2-HNA	0.2097	ns
Figure 4F	Areg+ ILC2s (%)	Kruskal Wallis with Dunns	Control vs FK866	<0.0001	***
Figure 4F	Areg+ ILC2s (%)	Kruskal Wallis with Dunns	Control vs FK866+NMN	>0.9999	ns
Figure 4F	Areg+ ILC2s (%)	Kruskal Wallis with Dunns	PA vs 2-HNA	>0.9999	ns

Figure 4F	Areg$^+$ ILC2s (%)	Kruskal Wallis with Dunns	PA vs FK866	0.0183	*
Figure 4F	Areg$^+$ ILC2s (%)	Kruskal Wallis with Dunns	PA vs FK866+NMN	>0.9999	ns
Figure 4F	Areg$^+$ ILC2s (%)	Kruskal Wallis with Dunns	2-HNA vs FK866	0.2573	ns
Figure 4F	Areg$^+$ ILC2s (%)	Kruskal Wallis with Dunns	2-HNA vs FK866+NMN	>0.9999	ns
Figure 4F	Areg$^+$ ILC2s (%)	Kruskal Wallis with Dunns	FK866 vs FK866+NMN	0.0379	*
Figure 4F	IL-5$^+$ ILC2s (%)	One-way ANOVA with Tukey	Control vs PA	0.9733	ns
Figure 4F	IL-5$^+$ ILC2s (%)	One-way ANOVA with Tukey	Control vs 2-HNA	>0.9999	ns
Figure 4F	IL-5$^+$ ILC2s (%)	One-way ANOVA with Tukey	Control vs FK866	<0.0001	***
Figure 4F	IL-5$^+$ ILC2s (%)	One-way ANOVA with Tukey	Control vs FK866+NMN	0.8029	ns
Figure 4F	IL-5$^+$ ILC2s (%)	One-way ANOVA with Tukey	PA vs 2-HNA	0.9654	ns
Figure 4F	IL-5$^+$ ILC2s (%)	One-way ANOVA with Tukey	PA vs FK866	<0.0001	***
Figure 4F	IL-5$^+$ ILC2s (%)	One-way ANOVA with Tukey	PA vs FK866+NMN	0.4439	ns
Figure 4F	IL-5$^+$ ILC2s (%)	One-way ANOVA with Tukey	2-HNA vs FK866	<0.0001	***
Figure 4F	IL-5$^+$ ILC2s (%)	One-way ANOVA with Tukey	2-HNA vs FK866+NMN	0.8254	ns
Figure 4F	IL-5$^+$ ILC2s (%)	One-way ANOVA with Tukey	FK866 vs FK866+NMN	<0.0001	***
Figure 4F	IL-13$^+$ ILC2s (%)	Kruskal Wallis with Dunns	Control vs PA	>0.9999	ns
Figure 4F	IL-13$^+$ ILC2s (%)	Kruskal Wallis with Dunns	Control vs 2-HNA	>0.9999	ns
Figure 4F	IL-13$^+$ ILC2s (%)	Kruskal Wallis with Dunns	Control vs FK866	0.0026	**
Figure 4F	IL-13$^+$ ILC2s (%)	Kruskal Wallis with Dunns	Control vs FK866+NMN	>0.9999	ns

Figure 4F	IL-13$^+$ ILC2s (%)	Kruskal Wallis with Dunns	PA vs 2-HNA	>0.9999	ns
Figure 4F	IL-13$^+$ ILC2s (%)	Kruskal Wallis with Dunns	PA vs FK866	0.0017	**
Figure 4F	IL-13$^+$ ILC2s (%)	Kruskal Wallis with Dunns	PA vs FK866+NMN	>0.9999	ns
Figure 4F	IL-13$^+$ ILC2s (%)	Kruskal Wallis with Dunns	2-HNA vs FK866	0.0270	*
Figure 4F	IL-13$^+$ ILC2s (%)	Kruskal Wallis with Dunns	2-HNA vs FK866+NMN	>0.9999	ns
Figure 4F	IL-13$^+$ ILC2s (%)	Kruskal Wallis with Dunns	FK866 vs FK866+NMN	0.1761	ns
Figure 4H	Areg$^+$ ILC2s (%)	One-way ANOVA with Tukey	Control vs FK866 0.2 µM	0.0295	*
Figure 4H	Areg$^+$ ILC2s (%)	One-way ANOVA with Tukey	Control vs FK866 1 µM	0.0002	***
Figure 4H	Areg$^+$ ILC2s (%)	One-way ANOVA with Tukey	FK866 0.2 µM vs FK866 1 µM	0.0404	*
Figure 5D	Pyruvate Total_response (AU/10^6 cells)	Unpaired t test	Control vs FK866	0.0375	*
Figure 5D	Pyruvate_M3 (AU/10^6 cells)	Unpaired t test	Control vs FK866	0.0355	*
Figure 5D	Acetyl-CoA Total_response (AU/10^6 cells)	Unpaired t test	Control vs FK866	0.2287	ns
Figure 5D	Acetyl-CoA_M2 (AU/10^6 cells)	Unpaired t test	Control vs FK866	0.0847	ns
Figure 5D	Citric acid Total_response (AU/10^6 cells)	Unpaired t test	Control vs FK866	0.2982	ns
Figure 5D	Citric acid_M2 (AU/10^6 cells)	Unpaired t test	Control vs FK866	0.1437	ns
Figure 5D	Isocitric acid Total_response (AU/10^6 cells)	Unpaired t test	Control vs FK866	0.0585	ns
Figure 5D	Isocitric acid_M2 (AU/10^6 cells)	Unpaired t test	Control vs FK866	0.0571	ns
Figure 5D	α-KG Total_response (AU/10^6 cells)	Unpaired t test	Control vs FK866	0.3348	ns
Figure 5D	α-KG_M2 (AU/10^6 cells)	Unpaired t test	Control vs FK866	0.6004	ns
Figure 5D	Succinate Total_response (AU/10^6 cells)	Unpaired t test	Control vs FK866	0.0217	*
Figure 5D	Succinate_M2 (AU/10^6 cells)	Unpaired t test	Control vs FK866	0.0101	*
Figure 5D	Fumarate Total_response (AU/10^6 cells)	Unpaired t test	Control vs FK866	0.6188	ns

Figure 5D	Fumarate_M2 (AU/10^6 cells)	Unpaired t test	Control vs FK866	0.4586	ns
Figure 5D	Malate Total_response (AU/10^6 cells)	Unpaired t test	Control vs FK866	0.0287	*
Figure 5D	Malate_M2 (AU/10^6 cells)	Unpaired t test	Control vs FK866	0.0114	*
Figure 5D	Oxaloacelic acid Total_response (AU/10^6 cells)	Unpaired t test	Control vs FK866	0.5651	ns
Figure 5D	Oxaloacelic acid_M2 (AU/10^6 cells)	Unpaired t test	Control vs FK866	0.3486	ns
Figure 5E	Succinate ng/10^6 cells FK866-	One-way ANOVA with Dunnett	Control vs α-KG	0.9998	ns
Figure 5E	Succinate ng/10^6 cells FK866-	One-way ANOVA with Dunnett	Control vs Succinate	0.0001	***
Figure 5E	Succinate ng/10^6 cells FK866-	One-way ANOVA with Dunnett	Control vs Fumarate	0.9997	ns
Figure 5E	Succinate ng/10^6 cells FK866-	One-way ANOVA with Dunnett	Control vs Malate	0.9997	ns
Figure 5E	Succinate ng/10^6 cells FK866-	One-way ANOVA with Dunnett	Control vs OAA	0.9656	ns
Figure 5E	Succinate ng/10^6 cells FK866+	One-way ANOVA with Dunnett	Control vs α-KG	0.9997	ns
Figure 5E	Succinate ng/10^6 cells FK866+	One-way ANOVA with Dunnett	Control vs Succinate	<0.0001	***
Figure 5E	Succinate ng/10^6 cells FK866+	One-way ANOVA with Dunnett	Control vs Fumarate	>0.9999	ns
Figure 5E	Succinate ng/10^6 cells FK866+	One-way ANOVA with Dunnett	Control vs Malate	0.9983	ns
Figure 5E	Succinate ng/10^6 cells FK866+	One-way ANOVA with Dunnett	Control vs OAA	>0.9999	ns
Figure 5E	NAD+ ng/10^6 cells FK866-	One-way ANOVA with Dunnett	Control vs α-KG	0.9467	ns
Figure 5E	NAD+ ng/10^6 cells FK866-	One-way ANOVA with Dunnett	Control vs Succinate	0.8135	ns
Figure 5E	NAD+ ng/10^6 cells FK866-	One-way ANOVA with Dunnett	Control vs Fumarate	0.3521	ns
Figure 5E	NAD+ ng/10^6 cells FK866-	One-way ANOVA with Dunnett	Control vs Malate	0.2100	ns
Figure 5E	NAD+ ng/10^6 cells FK866-	One-way ANOVA with Dunnett	Control vs OAA	0.9974	ns

Figure 5E	NAD+ ng/10^6 cells FK866+	One-way ANOVA with Dunnett	Control vs α-KG	0.5576	ns
Figure 5E	NAD+ ng/10^6 cells FK866+	One-way ANOVA with Dunnett	Control vs Succinate	0.8939	ns
Figure 5E	NAD+ ng/10^6 cells FK866+	One-way ANOVA with Dunnett	Control vs Fumarate	0.9999	ns
Figure 5E	NAD+ ng/10^6 cells FK866+	One-way ANOVA with Dunnett	Control vs Malate	>0.9999	ns
Figure 5E	NAD+ ng/10^6 cells FK866+	One-way ANOVA with Dunnett	Control vs OAA	0.9475	ns
Figure 5E	NADH ng/10^6 cells FK866-	One-way ANOVA with Dunnett	Control vs α-KG	0.0208	*
Figure 5E	NADH ng/10^6 cells FK866-	One-way ANOVA with Dunnett	Control vs Succinate	0.0448	*
Figure 5E	NADH ng/10^6 cells FK866-	One-way ANOVA with Dunnett	Control vs Fumarate	0.2531	ns
Figure 5E	NADH ng/10^6 cells FK866-	One-way ANOVA with Dunnett	Control vs Malate	0.4790	ns
Figure 5E	NADH ng/10^6 cells FK866-	One-way ANOVA with Dunnett	Control vs OAA	0.0206	*
Figure 5E	NADH ng/10^6 cells FK866+	Kruskal Wallis with Dunns	Control vs α-KG	>0.9999	ns
Figure 5E	NADH ng/10^6 cells FK866+	Kruskal Wallis with Dunns	Control vs Succinate	>0.9999	ns
Figure 5E	NADH ng/10^6 cells FK866+	Kruskal Wallis with Dunns	Control vs Fumarate	>0.9999	ns
Figure 5E	NADH ng/10^6 cells FK866+	Kruskal Wallis with Dunns	Control vs Malate	>0.9999	ns
Figure 5E	NADH ng/10^6 cells FK866+	Kruskal Wallis with Dunns	Control vs OAA	>0.9999	ns
Figure 5F	Areg$^+$ ILC2s (%) FK866-	One-way ANOVA with Dunnett	Control vs α-KG	0.8435	ns
Figure 5F	Areg$^+$ ILC2s (%) FK866-	One-way ANOVA with Dunnett	Control vs Succinate	0.9997	ns
Figure 5F	Areg$^+$ ILC2s (%) FK866-	One-way ANOVA with Dunnett	Control vs Fumarate	0.5421	ns
Figure 5F	Areg$^+$ ILC2s (%) FK866-	One-way ANOVA with Dunnett	Control vs Malate	0.2095	ns

Figure 5F	Areg$^+$ ILC2s (%) FK866-	One-way ANOVA with Dunnett	Control vs OAA	0.9593	ns
Figure 5F	Areg$^+$ ILC2s (%) FK866+	One-way ANOVA with Dunnett	Control vs α-KG	0.9997	ns
Figure 5F	Areg$^+$ ILC2s (%) FK866+	One-way ANOVA with Dunnett	Control vs Succinate	0.0085	**
Figure 5F	Areg$^+$ ILC2s (%) FK866+	One-way ANOVA with Dunnett	Control vs Fumarate	0.0761	ns
Figure 5F	Areg$^+$ ILC2s (%) FK866+	One-way ANOVA with Dunnett	Control vs Malate	0.9997	ns
Figure 5F	Areg$^+$ ILC2s (%) FK866+	One-way ANOVA with Dunnett	Control vs OAA	0.9766	ns
Figure 5F	IL-5$^+$ ILC2s (%) FK866-	One-way ANOVA with Dunnett	Control vs α-KG	0.2757	ns
Figure 5F	IL-5$^+$ ILC2s (%) FK866-	One-way ANOVA with Dunnett	Control vs Succinate	0.0900	ns
Figure 5F	IL-5$^+$ ILC2s (%) FK866-	One-way ANOVA with Dunnett	Control vs Fumarate	0.1330	ns
Figure 5F	IL-5$^+$ ILC2s (%) FK866-	One-way ANOVA with Tukey	Control vs Malate	0.9985	ns
Figure 5F	IL-5$^+$ ILC2s (%) FK866-	One-way ANOVA with Tukey	Control vs OAA	0.8920	ns
Figure 5F	IL-5$^+$ ILC2s (%) FK866+	One-way ANOVA with Dunnett	Control vs α-KG	0.8160	ns
Figure 5F	IL-5$^+$ ILC2s (%) FK866+	One-way ANOVA with Dunnett	Control vs Succinate	0.0002	***
Figure 5F	IL-5$^+$ ILC2s (%) FK866+	One-way ANOVA with Dunnett	Control vs Fumarate	0.5632	ns
Figure 5F	IL-5$^+$ ILC2s (%) FK866+	One-way ANOVA with Dunnett	Control vs Malate	0.8604	ns
Figure 5F	IL-5$^+$ ILC2s (%) FK866+	One-way ANOVA with Dunnett	Control vs OAA	0.9998	ns
Figure 5F	IL-13$^+$ ILC2s (%) FK866-	One-way ANOVA with Dunnett	Control vs α-KG	0.9996	ns
Figure 5F	IL-13$^+$ ILC2s (%) FK866-	One-way ANOVA with Dunnett	Control vs Succinate	0.7190	ns
Figure 5F	IL-13$^+$ ILC2s (%) FK866-	One-way ANOVA with Dunnett	Control vs Fumarate	0.9321	ns

Figure 5F	IL-13^{+} ILC2s (%) FK866-	One-way ANOVA with Dunnett	Control vs Malate	0.8290	ns
Figure 5F	IL-13^{+} ILC2s (%) FK866-	One-way ANOVA with Dunnett	Control vs OAA	0.9997	ns
Figure 5F	IL-13^{+} ILC2s (%) FK866+	One-way ANOVA with Tukey	Control vs α-KG	0.9753	ns
Figure 5F	IL-13^{+} ILC2s (%) FK866+	One-way ANOVA with Dunnett	Control vs Succinate	0.9812	ns
Figure 5F	IL-13^{+} ILC2s (%) FK866+	One-way ANOVA with Dunnett	Control vs Fumarate	0.9982	ns
Figure 5F	IL-13^{+} ILC2s (%) FK866+	One-way ANOVA with Dunnett	Control vs Malate	0.2717	ns
Figure 5F	IL-13^{+} ILC2s (%) FK866+	One-way ANOVA with Dunnett	Control vs OAA	0.9448	ns
Figure 6E	Areg^{+} ILC2s (%)	One-way ANOVA with Tukey	Control vs FK866	<0.0001	***
Figure 6E	Areg^{+} ILC2s (%)	One-way ANOVA with Tukey	Control vs FK866+Succinate	0.2367	ns
Figure 6E	Areg^{+} ILC2s (%)	One-way ANOVA with Tukey	Control vs FK866+Succinate+EM-K+	<0.0001	***
Figure 6E	Areg^{+} ILC2s (%)	One-way ANOVA with Tukey	FK866 vs FK866+Succinate	<0.0001	***
Figure 6E	Areg^{+} ILC2s (%)	One-way ANOVA with Tukey	FK866 vs FK866+Succinate+EM-K+	0.0099	**
Figure 6E	Areg^{+} ILC2s (%)	One-way ANOVA with Tukey	FK866+Succinate vs FK866+Succinate+EM-K+	<0.0001	***
Figure 6E	IL-5^{+} ILC2s (%)	One-way ANOVA with Tukey	Control vs FK866	<0.0001	***
Figure 6E	IL-5^{+} ILC2s (%)	One-way ANOVA with Tukey	Control vs FK866+Succinate	>0.9999	ns
Figure 6E	IL-5^{+} ILC2s (%)	One-way ANOVA with Tukey	Control vs FK866+Succinate+EM-K+	<0.0001	***
Figure 6E	IL-5^{+} ILC2s (%)	One-way ANOVA with Tukey	FK866 vs FK866+Succinate	<0.0001	***
Figure 6E	IL-5^{+} ILC2s (%)	One-way ANOVA with Tukey	FK866 vs FK866+Succinate+EM-K+	0.6904	ns
Figure 6E	IL-5^{+} ILC2s (%)	One-way ANOVA with Tukey	FK866+Succinate vs FK866+Succinate+EM-K+	<0.0001	***

Figure 6E	IL-13$^+$ ILC2s (%)	Kruskal Wallis with Dunns	Control vs FK866	0.0345	*
Figure 6E	IL-13$^+$ ILC2s (%)	Kruskal Wallis with Dunns	Control vs FK866+Succinate	0.2638	ns
Figure 6E	IL-13$^+$ ILC2s (%)	Kruskal Wallis with Dunns	Control vs FK866+Succinate+EM-K+	<0.0001	***
Figure 6E	IL-13$^+$ ILC2s (%)	Kruskal Wallis with Dunns	FK866 vs FK866+Succinate	>0.9999	ns
Figure 6E	IL-13$^+$ ILC2s (%)	Kruskal Wallis with Dunns	FK866 vs FK866+Succinate+EM-K+	0.3571	ns
Figure 6E	IL-13$^+$ ILC2s (%)	Kruskal Wallis with Dunns	FK866+Succinate vs FK866+Succinate+EM-K+	0.0510	ns
Figure 7B	ILC2s ($\times 10^4$)	Unpaired t test	Nampt$^{+/+}$Il5$^{RFP\text{-}Cre/+}$ vs Nampt$^{f/f}$Il5$^{RFP\text{-}Cre/+}$	<0.0001	***
Figure 7D	RFP+ (% of ILC2s)	Mann Whitney	Nampt$^{+/+}$Il5$^{RFP\text{-}Cre/+}$ vs Nampt$^{f/f}$Il5$^{RFP\text{-}Cre/+}$	0.0003	***
Figure 7F	Cells Nampt$^{+/+}$Il5$^{RFP\text{-}Cre/+}$	One-way ANOVA with Dunnett	Control vs NMN	0.4812	ns
Figure 7F	Cells Nampt$^{+/+}$Il5$^{RFP\text{-}Cre/+}$	One-way ANOVA with Dunnett	Control vs Succinate	0.4112	ns
Figure 7F	Cells Nampt$^{f/f}$Il5$^{RFP\text{-}Cre/+}$	One-way ANOVA with Dunnett	Control vs NMN	<0.0001	***
Figure 7F	Cells Nampt$^{f/f}$Il5$^{RFP\text{-}Cre/+}$	One-way ANOVA with Dunnett	Control vs Succinate	<0.0001	***
Figure 7F	Areg$^+$ ILC2s (%) Nampt$^{+/+}$Il5$^{RFP\text{-}Cre/+}$	One-way ANOVA with Dunnett	Control vs NMN	0.6474	ns
Figure 7F	Areg$^+$ ILC2s (%) Nampt$^{+/+}$Il5$^{RFP\text{-}Cre/+}$	One-way ANOVA with Dunnett	Control vs Succinate	0.7182	ns
Figure 7F	Areg$^+$ ILC2s (%) Nampt$^{f/f}$Il5$^{RFP\text{-}Cre/+}$	One-way ANOVA with Dunnett	Control vs NMN	0.0003	***
Figure 7F	Areg$^+$ ILC2s (%) Nampt$^{f/f}$Il5$^{RFP\text{-}Cre/+}$	One-way ANOVA with Dunnett	Control vs Succinate	0.0015	**
Figure 7F	IL-5$^+$ ILC2s (%) Nampt$^{+/+}$Il5$^{RFP\text{-}Cre/+}$	One-way ANOVA with Dunnett	Control vs NMN	0.1907	ns
Figure 7F	IL-5$^+$ ILC2s (%) Nampt$^{+/+}$Il5$^{RFP\text{-}Cre/+}$	One-way ANOVA with Dunnett	Control vs Succinate	0.0059	**
Figure 7F	IL-5$^+$ ILC2s (%) Nampt$^{f/f}$Il5$^{RFP\text{-}Cre/+}$	One-way ANOVA with Dunnett	Control vs NMN	<0.0001	***

Figure 7F	IL-5$^+$ ILC2s (%) Nampt$^{f/f}$Il5$^{RFP\text{-}Cre/+}$	One-way ANOVA with Dunnett	Control vs Succinate	<0.0001	***
Figure 7F	IL-13$^+$ ILC2s (%) Nampt$^{+/+}$Il5$^{RFP\text{-}Cre/+}$	One-way ANOVA with Dunnett	Control vs NMN	0.2948	ns
Figure 7F	IL-13$^+$ ILC2s (%) Nampt$^{+/+}$Il5$^{RFP\text{-}Cre/+}$	One-way ANOVA with Dunnett	Control vs Succinate	0.3340	ns
Figure 7F	IL-13$^+$ ILC2s (%) Nampt$^{f/f}$Il5$^{RFP\text{-}Cre/+}$	One-way ANOVA with Dunnett	Control vs NMN	<0.0001	***
Figure 7F	IL-13$^+$ ILC2s (%) Nampt$^{f/f}$Il5$^{RFP\text{-}Cre/+}$	One-way ANOVA with Dunnett	Control vs Succinate	<0.0001	***
Figure 7H	ILC2s (% from different donors)	Unpaired t test	WT vs cKO	<0.0001	***
Figure 7H	Areg$^+$ ILC2s (% from different donors)	Unpaired t test	WT vs cKO	<0.0001	***
Figure 7H	IL-5$^+$ ILC2s (% from different donors)	Unpaired t test	WT vs cKO	<0.0001	***
Figure 7H	IL-13$^+$ ILC2s (% from different donors)	Unpaired t test	WT vs cKO	<0.0001	***
Figure 7J	Colon length	Unpaired t test	Nampt$^{+/+}$Il5$^{RFP\text{-}Cre/+}$+DSS vs Nampt$^{f/f}$Il5$^{RFP\text{-}Cre/+}$+DSS	0.0006	***
Figure 7J	Colon length	Unpaired t test	Nampt$^{f/f}$Il5$^{RFP\text{-}Cre/+}$+DSS vs Nampt$^{f/f}$Il5$^{RFP\text{-}Cre/+}$+DSS+NMN	0.0003	***
Figure 7L	Histology scores	Unpaired t test	Nampt$^{+/+}$Il5$^{RFP\text{-}Cre/+}$+DSS vs Nampt$^{f/f}$Il5$^{RFP\text{-}Cre/+}$+DSS	0.0023	**
Figure 7L	Histology scores	Unpaired t test	Nampt$^{f/f}$Il5$^{RFP\text{-}Cre/+}$+DSS vs Nampt$^{f/f}$Il5$^{RFP\text{-}Cre/+}$+DSS+NMN	0.0006	***
Figure 7N	ILC2s ($\times 10^4$)	Unpaired t test	Nampt$^{+/+}$Il5$^{RFP\text{-}Cre/+}$+DSS vs Nampt$^{f/f}$Il5$^{RFP\text{-}Cre/+}$+DSS	<0.0001	***
Figure 7N	ILC2s ($\times 10^4$)	Unpaired t test	Nampt$^{f/f}$Il5$^{RFP\text{-}Cre/+}$+DSS vs Nampt$^{f/f}$Il5$^{RFP\text{-}Cre/+}$+DSS+NMN	0.0002	***
Figure 7N	Areg$^+$ ILC2s ($\times 10^4$)	Mann Whitney	Nampt$^{+/+}$Il5$^{RFP\text{-}Cre/+}$+DSS vs Nampt$^{f/f}$Il5$^{RFP\text{-}Cre/+}$+DSS	0.0006	***

Figure 7N	Areg$^+$ ILC2s (×10^4)	Unpaired t test	Nampt$^{f/f}$Il5$^{RFP-Cre/+}$+DSS vs Nampt$^{f/f}$Il5$^{RFP-Cre/+}$+DSS+NMN	0.0004	***
Figure 7N	IL-5$^+$ ILC2s (×10^4)	Mann Whitney	Nampt$^{+/+}$Il5$^{RFP-Cre/+}$+DSS vs Nampt$^{f/f}$Il5$^{RFP-Cre/+}$+DSS	0.0006	***
Figure 7N	IL-5$^+$ ILC2s (×10^4)	Unpaired t test	Nampt$^{f/f}$Il5$^{RFP-Cre/+}$+DSS vs Nampt$^{f/f}$Il5$^{RFP-Cre/+}$+DSS+NMN	0.0007	***
Figure 7N	IL-13$^+$ ILC2s (×10^4)	Mann Whitney	Nampt$^{+/+}$Il5$^{RFP-Cre/+}$+DSS vs Nampt$^{f/f}$Il5$^{RFP-Cre/+}$+DSS	0.0006	***
Figure 7N	IL-13$^+$ ILC2s (×10^4)	Unpaired t test	Nampt$^{f/f}$Il5$^{RFP-Cre/+}$+DSS vs Nampt$^{f/f}$Il5$^{RFP-Cre/+}$+DSS+NMN	0.0331	*
Figure S1A	UCEIS scores	Mann Whitney	ALT ≤1×ULN vs 1×ULN<ALT<2×ULN	0.0030	**
Figure S1A	UCEIS scores	Mann Whitney	1×ULN<ALT<2×ULN vs ALT ≥2×ULN	0.0004	***
Figure S1A	UCEIS scores	Mann Whitney	ALT ≤1×ULN vs ALT ≥2×ULN	<0.0001	***
Figure S1B	UCEIS scores	Mann Whitney	AST ≤1×ULN vs 1×ULN<AST<2×ULN	0.0113	*
Figure S1B	UCEIS scores	Mann Whitney	1×ULN<AST<2×ULN vs AST ≥2×ULN	0.0583	ns
Figure S1B	UCEIS scores	Mann Whitney	AST ≤1×ULN vs AST ≥2×ULN	0.0048	**
Figure S1C	Correlation analysis	Pearson	ALT and UCEIS	<0.0001	***
Figure S1D	Correlation analysis	Pearson	AST and UCEIS	<0.0001	***
Figure S1E	Colon length	Unpaired t test	Control vs CCl4	0.0886	ns
Figure S1I	Colon length	Unpaired t test	Sham vs BDL	0.2310	ns
Figure S2B	Plasma L-Serine	Unpaired t test	Healthy donor vs Liver cirrhosis	0.0015	**
Figure S2B	Plasma Niacinamide	Unpaired t test	Healthy donor vs Liver cirrhosis	0.0006	***
Figure S2B	Plasma Pantothenic Acid	Unpaired t test	Healthy donor vs Liver cirrhosis	0.0496	*
Figure S2B	Plasma Retinol	Unpaired t test	Healthy donor vs Liver cirrhosis	<0.0001	***
Figure S2B	Plasma Deoxycholic acid 3-glucuronide	Unpaired t test	Healthy donor vs Liver cirrhosis	0.0306	*
Figure S2B	Plasma L-Tyrosine	Unpaired t test	Healthy donor vs Liver cirrhosis	0.0005	***
Figure S2D	*TDO2*	Mann Whitney	Mild vs Advanced	0.0071	**

Figure S2D	*KMO*	Unpaired t test	Mild vs Advanced	0.0702	ns
Figure S2D	*KYNU*	Mann Whitney	Mild vs Advanced	0.2088	ns
Figure S2D	*HAAO*	Unpaired t test	Mild vs Advanced	<0.0001	***
Figure S2D	*QPRT*	Unpaired t test	Mild vs Advanced	<0.0001	***
Figure S2D	*NMNAT3*	Mann Whitney	Mild vs Advanced	0.0002	***
Figure S2D	*NADS*	Mann Whitney	Mild vs Advanced	0.0476	*
Figure S2D	*NAMPT*	Mann Whitney	Mild vs Advanced	0.6505	ns
Figure S2E	*TDO2*	Unpaired t test	HC vs ALD	0.0055	**
Figure S2E	*KMO*	Unpaired t test	HC vs ALD	<0.0001	***
Figure S2E	*KYNU*	Mann Whitney	HC vs ALD	0.8467	ns
Figure S2E	*HAAO*	Unpaired t test	HC vs ALD	<0.0001	***
Figure S2E	*QPRT*	Unpaired t test	HC vs ALD	0.0133	*
Figure S2E	*NMNAT2*	Mann Whitney	HC vs ALD	0.7727	ns
Figure S2E	*NMNAT3*	Mann Whitney	HC vs ALD	0.2027	ns
Figure S2E	*NAMPT*	Unpaired t test	HC vs ALD	<0.0001	***
Figure S2F	Liver NAD+	Unpaired t test	Sham vs BDL	0.0409	*
Figure S2F	Plasma NAM	Mann Whitney	Sham vs BDL	0.0079	**
Figure S2F	Intestine NAD+	Unpaired t test	Sham vs BDL	0.0017	**
Figure S2G	*Tdo2*	Unpaired t test	Control vs MAFLD	0.0678	ns
Figure S2G	*Ido2*	Unpaired t test	Control vs MAFLD	0.0003	***
Figure S2G	*Kmo*	Unpaired t test	Control vs MAFLD	<0.0001	***
Figure S2G	*Kynu*	Unpaired t test	Control vs MAFLD	0.0002	***
Figure S2G	*Haao*	Unpaired t test	Control vs MAFLD	<0.0001	***
Figure S2G	*Qprt*	Unpaired t test	Control vs MAFLD	0.0002	***
Figure S2G	*Nmnat1*	Unpaired t test	Control vs MAFLD	0.0090	**
Figure S2G	*Nmnat2*	Unpaired t test	Control vs MAFLD	0.5295	ns
Figure S2G	*Nmnat3*	Unpaired t test	Control vs MAFLD	0.0034	**
Figure S2G	*Nads*	Unpaired t test	Control vs MAFLD	0.4603	ns
Figure S2G	*Nampt*	Unpaired t test	Control vs MAFLD	0.0011	**
Figure S2H	*Tdo2*	Unpaired t test	Sham vs BDL-48 h	0.0049	**
Figure S2H	*Kmo*	Unpaired t test	Sham vs BDL-48 h	0.0018	**

Figure S2H	*Kynu*	Unpaired t test	Sham vs BDL-48 h	0.0001	***
Figure S2H	*Haao*	Unpaired t test	Sham vs BDL-48 h	0.0571	ns
Figure S2H	*Qprt*	Unpaired t test	Sham vs BDL-48 h	0.2277	ns
Figure S2H	*Nmnat1*	Unpaired t test	Sham vs BDL-48 h	0.0090	**
Figure S2H	*Nmnat2*	Unpaired t test	Sham vs BDL-48 h	0.0946	ns
Figure S2H	*Nmnat3*	Unpaired t test	Sham vs BDL-48 h	0.6240	ns
Figure S2H	*Nampt*	Unpaired t test	Sham vs BDL-48 h	0.0030	**
Figure S2I	*Tdo2*	Unpaired t test	Sham vs BDL-28 d	0.1605	ns
Figure S2I	*Kmo*	Unpaired t test	Sham vs BDL-28 d	0.0009	***
Figure S2I	*Kynu*	Mann Whitney	Sham vs BDL-28 d	0.1000	ns
Figure S2I	*Haao*	Unpaired t test	Sham vs BDL-28 d	0.0259	*
Figure S2I	*Qprt*	Unpaired t test	Sham vs BDL-28 d	0.2463	ns
Figure S2I	*Nmnat1*	Unpaired t test	Sham vs BDL-28 d	0.0090	**
Figure S2I	*Nmnat2*	Mann Whitney	Sham vs BDL-28 d	0.2000	ns
Figure S2I	*Nmnat3*	Unpaired t test	Sham vs BDL-28 d	0.8438	ns
Figure S2I	*Nampt*	Unpaired t test	Sham vs BDL-28 d	0.0037	**
Figure S3B	Plasma NAM	Unpaired t test	Control vs CCl4	0.0333	*
Figure S3B	Plasma NAM	Unpaired t test	CCl4 vs CCl4+NMN	<0.0001	***
Figure S3B	Intestine NAD+	Unpaired t test	Control vs CCl4	0.0472	*
Figure S3B	Intestine NAD+	Unpaired t test	CCl4 vs CCl4+NMN	0.0005	***
Figure S3C	ILC2s ($\times 10^4$)	Mann Whitney	Control vs CCl4	0.0079	**
Figure S3C	ILC2s ($\times 10^4$)	Mann Whitney	CCl4 vs CCl4+NMN	0.0079	**
Figure S3C	Areg$^+$ ILC2s ($\times 10^4$)	Unpaired t test	Control vs CCl4	<0.0001	***
Figure S3C	Areg$^+$ ILC2s ($\times 10^4$)	Unpaired t test	CCl4 vs CCl4+NMN	0.0060	**
Figure S3C	IL-5$^+$ ILC2s ($\times 10^4$)	Unpaired t test	Control vs CCl4	0.0003	**
Figure S3C	IL-5$^+$ ILC2s ($\times 10^4$)	Unpaired t test	CCl4 vs CCl4+NMN	0.0110	*
Figure S3C	IL-13$^+$ ILC2s ($\times 10^4$)	Unpaired t test	Control vs CCl4	<0.0001	***
Figure S3C	IL-13$^+$ ILC2s ($\times 10^4$)	Unpaired t test	CCl4 vs CCl4+NMN	0.0006	***
Figure S3D	Areg$^+$ ILC2s (%)	Unpaired t test	Control vs CCl4	0.0023	**
Figure S3D	Areg$^+$ ILC2s (%)	Unpaired t test	CCl4 vs CCl4+NMN	0.0500	ns
Figure S3D	IL-5$^+$ ILC2s (%)	Unpaired t test	Control vs CCl4	0.0032	**

Figure S3D	IL-5+ ILC2s (%)	Unpaired t test	CCl4 vs CCl4+NMN	0.0214	*
Figure S3D	IL-13+ ILC2s (%)	Unpaired t test	Control vs CCl4	0.0002	***
Figure S3D	IL-13+ ILC2s (%)	Unpaired t test	CCl4 vs CCl4+NMN	0.0016	**
Figure S3E	Relative expression Il1β	Unpaired t test	DSS vs CCl4+DSS	0.0058	**
Figure S3E	Relative expression Il1β	Unpaired t test	CCl4+DSS vs CCl4+DSS+NMN	0.0006	***
Figure S3E	Relative expression Il6	Unpaired t test	DSS vs CCl4+DSS	0.0156	*
Figure S3E	Relative expression Il6	Mann Whitney	CCl4+DSS vs CCl4+DSS+NMN	0.0317	*
Figure S3E	Relative expression Tnfα	Mann Whitney	DSS vs CCl4+DSS	0.4206	ns
Figure S3E	Relative expression Tnfα	Mann Whitney	CCl4+DSS vs CCl4+DSS+NMN	0.0397	*
Figure S3G	Colon length	Unpaired t test	Sham+DSS vs BDL+DSS	0.0109	*
Figure S3G	Colon length	Unpaired t test	BDL+DSS vs BDL+DSS+NMN	0.0016	**
Figure S3H	Histology scores	Mann Whitney	Sham+DSS vs BDL+DSS	0.0397	*
Figure S3H	Histology scores	Unpaired t test	BDL+DSS vs BDL+DSS+NMN	0.0133	*
Figure S3I	Relative expression Qprt	Mann Whitney	Control vs Qprt KD	0.0286	*
Figure S3J	Intestine NAD+	Unpaired t test	DSS vs. *Qprt* KD+DSS	0.0080	**
Figure S3J	Intestine NAD+	Unpaired t test	*Qprt* KD+DSS vs. *Qprt* KD+DSS+NMN	0.0005	***
Figure S3K	Areg+ ILC2s (%)	Unpaired t test	Control vs Qprt KD	0.0195	*
Figure S3K	IL-5+ ILC2s (%)	Unpaired t test	Control vs Qprt KD	0.0070	**
Figure S3K	IL-13+ ILC2s (%)	Unpaired t test	Control vs Qprt KD	0.0096	**
Figure S4C	Areg+ ILC2s (%)	Unpaired t test	Control vs CCl4	<0.0001	***
Figure S4C	IL-5+ ILC2s (%)	Unpaired t test	Control vs CCl4	0.0002	***
Figure S4C	IL-13+ ILC2s (%)	Unpaired t test	Control vs CCl4	0.0001	***
Figure S4D	ILC2s ($\times10^4$)	Unpaired t test	DSS vs CCl4+DSS	0.0164	*
Figure S4D	Areg+ ILC2s ($\times10^4$)	Unpaired t test	DSS vs CCl4+DSS	0.0009	***
Figure S4D	IL-5+ ILC2s ($\times10^4$)	Unpaired t test	DSS vs CCl4+DSS	0.0265	*
Figure S4D	IL-13+ ILC2s ($\times10^4$)	Unpaired t test	DSS vs CCl4+DSS	0.0061	**
Figure S4D	Areg+ ILC2s (%)	Unpaired t test	DSS vs CCl4+DSS	0.0001	***
Figure S4D	IL-5+ ILC2s (%)	Unpaired t test	DSS vs CCl4+DSS	0.1400	ns
Figure S4D	IL-13+ ILC2s (%)	Mann Whitney	DSS vs CCl4+DSS	0.0556	ns
Figure S4F	Areg+ ILC2s (%)	Unpaired t test	Sham vs BDL	0.0419	*

Figure S4F	IL-5$^+$ ILC2s (%)	Unpaired t test	Sham vs BDL	0.0073	**
Figure S4F	IL-13$^+$ ILC2s (%)	Unpaired t test	Sham vs BDL	0.0368	*
Figure S4G	ILC2s (×10^4)	Unpaired t test	Sham vs BDL	0.0244	*
Figure S4G	Areg$^+$ ILC2s (×10^4)	Unpaired t test	Sham vs BDL	0.0036	**
Figure S4G	IL-5$^+$ ILC2s (×10^4)	Unpaired t test	Sham vs BDL	<0.0001	***
Figure S4G	IL-13$^+$ ILC2s (×10^4)	Unpaired t test	Sham vs BDL	0.0030	**
Figure S4H	ILC2s (×10^4)	Mann Whitney	Sham+DSS vs BDL+DSS	0.0079	**
Figure S4H	Areg$^+$ ILC2s (×10^4)	Unpaired t test	Sham+DSS vs BDL+DSS	0.0029	**
Figure S4H	IL-5$^+$ ILC2s (×10^4)	Unpaired t test	Sham+DSS vs BDL+DSS	0.0171	*
Figure S4H	IL-13$^+$ ILC2s (×10^4)	Unpaired t test	Sham+DSS vs BDL+DSS	0.0329	*
Figure S4H	Areg$^+$ ILC2s (%)	Unpaired t test	Sham+DSS vs BDL+DSS	0.0120	*
Figure S4H	IL-5$^+$ ILC2s (%)	Unpaired t test	Sham+DSS vs BDL+DSS	0.0199	*
Figure S4H	IL-13$^+$ ILC2s (%)	Unpaired t test	Sham+DSS vs BDL+DSS	0.0746	ns
Figure S5C	Pyruvate_M0 Fractional contribution (%)	Unpaired t test	Control vs FK866	0.0116	*
Figure S5C	Pyruvate_M1 Fractional contribution (%)	Unpaired t test	Control vs FK866	0.0131	*
Figure S5C	Pyruvate_M2 Fractional contribution (%)	Unpaired t test	Control vs FK866	0.0117	*
Figure S5C	Pyruvate_M3 Fractional contribution (%)	Unpaired t test	Control vs FK866	0.0119	*
Figure S5C	Acetyl-CoA _M0 Fractional contribution (%)	Unpaired t test	Control vs FK866	0.4265	ns
Figure S5C	Acetyl-CoA_M1 Fractional contribution (%)	Unpaired t test	Control vs FK866	0.8255	ns
Figure S5C	Acetyl-CoA_M2 Fractional contribution (%)	Unpaired t test	Control vs FK866	0.441	ns
Figure S5C	Citric acid_M0 Fractional contribution (%)	Unpaired t test	Control vs FK866	0.1387	ns
Figure S5C	Citric acid_M1 Fractional contribution (%)	Unpaired t test	Control vs FK866	0.0677	ns
Figure S5C	Citric acid_M2 Fractional contribution (%)	Unpaired t test	Control vs FK866	0.0761	ns
Figure S5C	Citric acid_M3 Fractional contribution (%)	Unpaired t test	Control vs FK866	0.4297	ns
Figure S5C	Citric acid_M4 Fractional contribution (%)	Unpaired t test	Control vs FK866	0.0612	ns
Figure S5C	Citric acid_M5 Fractional contribution (%)	Unpaired t test	Control vs FK866	0.656	ns
Figure S5C	Isocitric acid_M0 Fractional contribution (%)	Unpaired t test	Control vs FK866	0.3171	ns

Figure S5C	Isocitric acid_M1 Fractional contribution (%)	Unpaired t test	Control vs FK866	0.2125	ns
Figure S5C	Isocitric acid_M2 Fractional contribution (%)	Unpaired t test	Control vs FK866	0.3619	ns
Figure S5C	Isocitric acid_M3 Fractional contribution (%)	Unpaired t test	Control vs FK866	0.8633	ns
Figure S5C	α-KG_M0 Fractional contribution (%)	Unpaired t test	Control vs FK866	0.2691	ns
Figure S5C	α-KG_M1 Fractional contribution (%)	Unpaired t test	Control vs FK866	0.0486	*
Figure S5C	α-KG_M2 Fractional contribution (%)	Unpaired t test	Control vs FK866	0.2111	ns
Figure S5C	α-KG_M3 Fractional contribution (%)	Unpaired t test	Control vs FK866	0.9821	ns
Figure S5C	α-KG_M5 Fractional contribution (%)	Unpaired t test	Control vs FK866	0.5896	ns
Figure S5C	Succinate_M0 Fractional contribution (%)	Unpaired t test	Control vs FK866	0.7782	ns
Figure S5C	Succinate_M1 Fractional contribution (%)	Unpaired t test	Control vs FK866	0.3432	ns
Figure S5C	Succinate_M2 Fractional contribution (%)	Unpaired t test	Control vs FK866	0.8928	ns
Figure S5C	Succinate_M3 Fractional contribution (%)	Unpaired t test	Control vs FK866	0.0957	ns
Figure S5C	Succinate_M4 Fractional contribution (%)	Unpaired t test	Control vs FK866	0.0198	*
Figure S5C	Fumarate_M0 Fractional contribution (%)	Mann Whitney	Control vs FK866	0.7000	ns
Figure S5C	Fumarate_M1 Fractional contribution (%)	Unpaired t test	Control vs FK866	0.2623	ns
Figure S5C	Fumarate_M2 Fractional contribution(%)	Unpaired t test	Control vs FK866	0.784	ns
Figure S5C	Fumarate_M3 Fractional contribution (%)	Unpaired t test	Control vs FK866	0.4427	ns
Figure S5C	Malate_M0 Fractional contribution (%)	Unpaired t test	Control vs FK866	0.0126	*
Figure S5C	Malate_M1 Fractional contribution (%)	Unpaired t test	Control vs FK866	0.0754	ns
Figure S5C	Malate_M2 Fractional contribution (%)	Unpaired t test	Control vs FK866	0.0613	ns
Figure S5C	Malate_M3 Fractional contribution (%)	Mann Whitney	Control vs FK866	0.1000	ns
Figure S5C	Malate_M4 Fractional contribution (%)	Unpaired t test	Control vs FK866	0.0655	ns
Figure S5C	Oxaloacelic acid_M0 Fractional contribution (%)	Unpaired t test	Control vs FK866	0.7643	ns
Figure S5C	Oxaloacelic acid_M1 Fractional contribution (%)	Unpaired t test	Control vs FK866	0.2938	ns
Figure S5C	Oxaloacelic acid_M2 Fractional contribution (%)	Unpaired t test	Control vs FK866	0.3276	ns

Figure S5C	Oxaloacelic acid_M3 Fractional contribution (%)	Mann Whitney	Control vs FK866	>0.9999	ns
Figure S5D	α-KG (ng/10^6 cells) FK866(-)	Kruskal Wallis with Dunns	Control vs α-KG	0.0347	*
Figure S5D	α-KG (ng/10^6 cells) FK866(-)	Kruskal Wallis with Dunns	Control vs Succinate	>0.9999	ns
Figure S5D	α-KG (ng/10^6 cells) FK866(-)	Kruskal Wallis with Dunns	Control vs Fumarate	>0.9999	ns
Figure S5D	α-KG (ng/10^6 cells) FK866(-)	Kruskal Wallis with Dunns	Control vs Malate	>0.9999	ns
Figure S5D	α-KG (ng/10^6 cells) FK866(-)	Kruskal Wallis with Dunns	Control vs OAA	0.3216	ns
Figure S5D	α-KG (ng/10^6 cells) FK866(+)	One-way ANOVA with Dunnett	Control vs α-KG	<0.0001	***
Figure S5D	α-KG (ng/10^6 cells) FK866(+)	One-way ANOVA with Dunnett	Control vs Succinate	0.9999	ns
Figure S5D	α-KG (ng/10^6 cells) FK866(+)	One-way ANOVA with Dunnett's	Control vs Fumarate	0.9999	ns
Figure S5D	α-KG (ng/10^6 cells) FK866(+)	One-way ANOVA with Dunnett	Control vs Malate	0.9998	ns
Figure S5D	α-KG (ng/10^6 cells) FK866(+)	One-way ANOVA with Dunnett	Control vs OAA	0.9999	ns
Figure S5D	Fumarate (ng/10^6 cells) FK866(-)	One-way ANOVA with Dunnett	Control vs α-KG	0.9999	ns
Figure S5D	Fumarate (ng/10^6 cells) FK866(-)	One-way ANOVA with Dunnett	Control vs Succinate	0.0133	*
Figure S5D	Fumarate (ng/10^6 cells) FK866(-)	One-way ANOVA with Dunnett's	Control vs Fumarate	0.0929	ns
Figure S5D	Fumarate (ng/10^6 cells) FK866(-)	One-way ANOVA with Dunnett	Control vs Malate	0.2062	ns
Figure S5D	Fumarate (ng/10^6 cells) FK866(-)	One-way ANOVA with Dunnett	Control vs OAA	0.5403	ns
Figure S5D	Fumarate (ng/10^6 cells) FK866(+)	One-way ANOVA with Dunnett	Control vs α-KG	0.9999	ns
Figure S5D	Fumarate (ng/10^6 cells) FK866(+)	One-way ANOVA with Dunnett	Control vs Succinate	0.0366	*
Figure S5D	Fumarate (ng/10^6 cells) FK866(+)	One-way ANOVA with Dunnett	Control vs Fumarate	0.1439	ns

Figure S5D	Fumarate (ng/10^6 cells) FK866(+)	One-way ANOVA with Dunnett	Control vs Malate	0.9997	ns
Figure S5D	Fumarate (ng/10^6 cells) FK866(+)	One-way ANOVA with Dunnett	Control vs OAA	0.9997	ns
Figure S5D	Malate (ng/10^6 cells) FK866(-)	One-way ANOVA with Dunnett	Control vs α-KG	0.5622	ns
Figure S5D	Malate (ng/10^6 cells) FK866(-)	One-way ANOVA with Dunnett	Control vs Succinate	0.0124	*
Figure S5D	Malate (ng/10^6 cells) FK866(-)	One-way ANOVA with Dunnett	Control vs Fumarate	0.9739	ns
Figure S5D	Malate (ng/10^6 cells) FK866(-)	One-way ANOVA with Dunnett	Control vs Malate	<0.0001	***
Figure S5D	Malate (ng/10^6 cells) FK866(-)	One-way ANOVA with Dunnett	Control vs OAA	0.1673	ns
Figure S5D	Malate (ng/10^6 cells) FK866(+)	One-way ANOVA with Dunnett	Control vs α-KG	0.9968	ns
Figure S5D	Malate (ng/10^6 cells) FK866(+)	One-way ANOVA with Dunnett	Control vs Succinate	0.0778	ns
Figure S5D	Malate (ng/10^6 cells) FK866(+)	One-way ANOVA with Dunnett	Control vs Fumarate	0.9726	ns
Figure S5D	Malate (ng/10^6 cells) FK866(+)	One-way ANOVA with Dunnett	Control vs Malate	0.853	ns
Figure S5D	Malate (ng/10^6 cells) FK866(+)	One-way ANOVA with Dunnett	Control vs OAA	>0.9999	ns
Figure S5D	OAA (ng/10^6 cells) FK866(-)	Kruskal Wallis with Dunns	Control vs α-KG	>0.9999	ns
Figure S5D	OAA (ng/10^6 cells) FK866(-)	Kruskal Wallis with Dunns	Control vs Succinate	0.968	ns
Figure S5D	OAA (ng/10^6 cells) FK866(-)	Kruskal Wallis with Dunns	Control vs Fumarate	>0.9999	ns
Figure S5D	OAA (ng/10^6 cells) FK866(-)	Kruskal Wallis with Dunns	Control vs Malate	0.3593	ns
Figure S5D	OAA (ng/10^6 cells) FK866(-)	Kruskal Wallis with Dunns	Control vs OAA	0.2275	ns
Figure S5D	OAA (ng/10^6 cells) FK866(+)	Kruskal Wallis with Dunns	Control vs α-KG	0.4006	ns
Figure S5D	OAA (ng/10^6 cells) FK866(+)	Kruskal Wallis with Dunns	Control vs Succinate	>0.9999	ns

Figure S5D	OAA (ng/10^6 cells) FK866(+)	Kruskal Wallis with Dunns	Control vs Fumarate	0.548	ns
Figure S5D	OAA (ng/10^6 cells) FK866(+)	Kruskal Wallis with Dunns	Control vs Malate	>0.9999	ns
Figure S5D	OAA (ng/10^6 cells) FK866(+)	Kruskal Wallis with Dunns	Control vs OAA	>0.9999	ns
Figure S5D	NAD+/NADH FK866(-)	One-way ANOVA with Dunnett	Control vs α-KG	0.2022	ns
Figure S5D	NAD+/NADH FK866(-)	One-way ANOVA with Dunnett	Control vs Succinate	0.9947	ns
Figure S5D	NAD+/NADH FK866(-)	One-way ANOVA with Dunnett	Control vs Fumarate	0.9998	ns
Figure S5D	NAD+/NADH FK866(-)	One-way ANOVA with Dunnett	Control vs Malate	0.9983	ns
Figure S5D	NAD+/NADH FK866(-)	One-way ANOVA with Dunnett	Control vs OAA	0.2648	ns
Figure S5D	NAD+/NADH FK866(+)	Kruskal Wallis with Dunns	Control vs α-KG	>0.9999	ns
Figure S5D	NAD+/NADH FK866(+)	Kruskal Wallis with Dunns	Control vs Succinate	>0.9999	ns
Figure S5D	NAD+/NADH FK866(+)	Kruskal Wallis with Dunns	Control vs Fumarate	>0.9999	ns
Figure S5D	NAD+/NADH FK866(+)	Kruskal Wallis with Dunns	Control vs Malate	>0.9999	ns
Figure S5D	NAD+/NADH FK866(+)	Kruskal Wallis with Dunns	Control vs OAA	>0.9999	ns
Figure S6D	$Areg^+$ ILC2s (×10^4)	Unpaired t test	$Nampt^{+/+}Il5^{RFP\text{-}Cre/+}$ vs $Nampt^{f/f}Il5^{RFP\text{-}Cre/+}$	<0.0001	***
Figure S6D	$IL\text{-}5^+$ ILC2s (×10^4)	Unpaired t test	$Nampt^{+/+}Il5^{RFP\text{-}Cre/+}$ vs $Nampt^{f/f}Il5^{RFP\text{-}Cre/+}$	0.0005	***
Figure S6D	$IL\text{-}13^+$ ILC2s (×10^4)	Unpaired t test	$Nampt^{+/+}Il5^{RFP\text{-}Cre/+}$ vs $Nampt^{f/f}Il5^{RFP\text{-}Cre/+}$	0.0011	**
Figure S6E	$Areg^+$ ILC2s (%)	Mann Whitney	$Nampt^{+/+}Il5^{RFP\text{-}Cre/+}$ vs $Nampt^{f/f}Il5^{RFP\text{-}Cre/+}$	0.1304	ns
Figure S6E	$IL\text{-}5^+$ ILC2s (%)	Unpaired t test	$Nampt^{+/+}Il5^{RFP\text{-}Cre/+}$ vs $Nampt^{f/f}Il5^{RFP\text{-}Cre/+}$	0.0497	*
Figure S6E	$IL\text{-}13^+$ ILC2s (%)	Unpaired t test	$Nampt^{+/+}Il5^{RFP\text{-}Cre/+}$ vs $Nampt^{f/f}Il5^{RFP\text{-}Cre/+}$	0.2817	ns

Table S5 Antibodies used for flow cytometry

Antibodies	Source	Identifier
Anti-mouse CD16/CD32 (clone 93)	eBioscience	Cat# 14-0161-85

Anti-mouse CD45.1 PE-Cyanine7 (clone A20)	eBioscience	Cat# 25-0453-82
Anti-mouse CD45.2 Alexa Fluor 700 (clone 104)	eBioscience	Cat# 56-0454-82
Anti-mouse CD3e APC-eFlour 780 (clone145-2C11)	eBioscience	Cat# 47-0031-82
Anti-mouse CD5 APC-eFlour 780 (clone 53-7.3)	eBioscience	Cat# 47-0051-82
Anti-mouse CD19 APC-eFlour 780 (clone 1D3)	eBioscience	Cat# 47-0193-82
Anti-mouse/human CD45R/B220 APC/Cyanine7 (clone RA3-6B2)	Biolegend	Cat# 103224
Anti-mouse CD16/32 APC/Cyanine7 (clone 93)	Biolegend	Cat# 101328
Anti-mouse FcεRIα APC/Cyanine7 (clone MAR-1)	Biolegend	Cat# 134326
Anti-mouse/human CD11b APC/Cyanine7 (clone M1/70)	Tonbo Biosciences	Cat# 25-0112
Anti-mouse CD11c APC/Cyanine7 (clone N418)	Biolegend	Cat# 117324
Anti-mouse Ly-6G APC/Cyanine7 (clone 1A8)	Tonbo Biosciences	Cat# 25-1276
Anti-mouse TER-119 APC-eFlour 780 (clone TER-119)	eBioscience	Cat# 47-5921-82
Anti-mouse CD3e FITC (clone 145-2C11)	Tonbo Biosciences	Cat# 35-0031
Anti-Mouse CD5 FITC (clone 53-7.3)	Tonbo Biosciences	Cat# 35-0051
Anti-mouse CD16/32 FITC (clone 2.4G2)	Tonbo Biosciences	Cat# 35-0161
Anti-mouse/human CD11b FITC (clone M1/70)	Tonbo Biosciences	Cat# 35-0112
Anti-mouse CD11c FITC (clone N418)	Tonbo Biosciences	Cat# 35-0114
Anti-mouse CD19 FITC (clone 1D3)	Tonbo Biosciences	Cat# 35-0193
Anti-mouse/human CD45R/B220 FITC (clone RA3-6B2)	Tonbo Biosciences	Cat# 35-0452
Anti-mouse TER119 FITC (clone TER-119)	Tonbo Biosciences	Cat# 35-5921
Anti-mouse Ly-6G FITC (clone 1A8)	Tonbo Biosciences	Cat# 35-1276
Anti-mouse FcεRIα FITC (clone MAR-1)	Tonbo Biosciences	Cat# 35-5898
Anti-mouse IL-13 Alexa Fluor 488 (clone eBio13A)	eBioscience	Cat# 53-7133-82
Anti-mouse/human IL-5 APC (clone TRFK5)	Biolegend	Cat# 504306
Anti-mouse/human IL-5 PE (clone TRFK5)	BD Pharmingen	Cat# 554395
Anti-mouse GATA3 BV421 (clone A20)	BD Horizon	Cat# 563349
Anti-mouse KLRG1 PerCP/Cy5.5 (clone 2F1)	eBioscience	Cat# 46-5893-82
Anti-mouse CD127(IL-17Ra) APC (clone A7R34)	Biolegend	Cat# 135012
Anti-mouse Amphiregulin biotin	R&D Systems	Cat# BAF989
Anti-human CD303α FITC (clone 201A)	eBioscience	Cat# 11-9818-42
Anti-human CD94 FITC (clone DX22)	eBioscience	Cat# 11-0949-42
Anti-human CD19 FITC (clone SJ25C1)	Tonbo Biosciences	Cat# 35-0198-T025
Anti-human CD123 FITC (clone 6H6)	eBioscience	Cat# 11-1239-42
Anti-human CD3 FITC (clone SK7)	eBioscience	Cat# 11-0036-42
Anti-human CD16 FITC (clone CB16)	eBioscience	Cat# 11-0168-42
Anti-human CD14 FITC (clone 61D3)	Tonbo Biosciences	Cat# 35-0149-T025
Anti-human CD45 PE-Cyanine7 (H130)	Biolegend	Cat# 304016
Anti-human CD127(IL-17Ra) APC (clone A019D5)	Biolegend	Cat# 351310
Anti-human CD294 (CRTH2) APC/Cyanine7 (clone BM16)	Biolegend	Cat# 350114
PE-Cyanine7 Streptavidin	eBioscience	Cat# 25-4317-82
APC Streptavidin	Biolegend	Cat# 405207

Table S6 Primer sequences used for qRT-PCR

Gene	Forward primer	Reverse primer
Gapdh	CATGGCCTCCAAGGAGTAAG	CCTAGGCCCCTCCTGTTATT

Il1b	GAAATGCCACCTTTTGACAGTG	TGGATGCTCTCATCAGGACAG
Il6	TACCACTTCACAAGTCGGAGGC	CTGCAAGTGCATCATCGTTGTTC
Tnfa	CAGGCGGTGCCTATGTCTC	CGATCACCCCGAAGTTCAGTAG
Tdo2	TGGCAATTACTTGCAGTTGGA	GTGCTCGTCATGGATTTTGTTC
Ido2	TGGGGAGATACCACATTTCTGA	TGAGGAAGTCTGAGGGCAATTT
Kmo	ATGGCATCGTCTGATACTCAGG	AGCTTCGTACACATCAACTTGAA
Kynu	CCTTCGCCTCTTGAGCTTCC	AGAGCAACCCTCTCATCTGTT
Qprt	CATCCTTGGTTACCGGGTCG	GCCAGGGTGTTAAGAGCCA
Nmnat1	TGGCTCTTTTAACCCCATCAC	TCTTCTTGTACGCATCACCGA
Nmnat2	ATGACCGAGACCACAAAGACC	ATCCCGCCAATCACAATAAATCT
Nmnat3	ATCACGAATATGCACCTGCG	ATTGACGGGTGAGATGATGCC
Nampt	GCAGAAGCCGAGTTCAACATC	TTTTCACGGCATTCAAAGTAGGA
